# Structural Snapshots in Reversible Phosphinidene Transfer: Synthetic, Structural, and Reaction Chemistry of a Sn=P Double Bond

**DOI:** 10.1021/jacs.2c03302

**Published:** 2022-05-10

**Authors:** Malte Fischer, Matthew M. D. Roy, Lewis L. Wales, Mathias A. Ellwanger, Andreas Heilmann, Simon Aldridge

**Affiliations:** † Inorganic Chemistry Laboratory, Department of Chemistry, University of Oxford, South Parks Road, Oxford OX1 3QR, U.K.; ‡ Department of Chemistry, Catalysis Research Center and Institute for Silicon Chemistry, Technische Universität München, 85748 Garching bei München, Germany

## Abstract

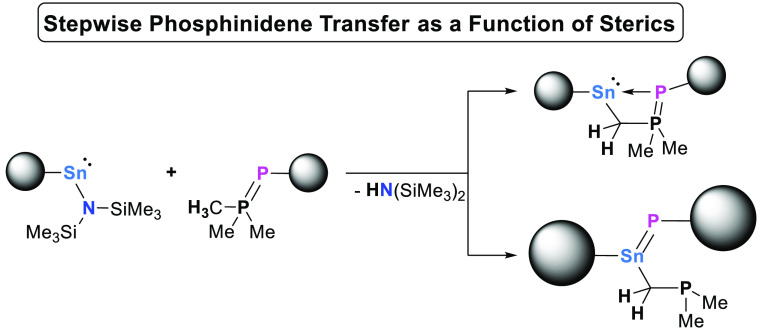

The reaction of amido-substituted stannylenes with phospha-Wittig reagents (Me_3_PPR) results in release of hexamethyldisilazane and tethering of the resulting −CH_2_PMe_2_PR fragment to the tin center to give P-donor stabilized stannylenes featuring four-membered *Sn*,*C*,*P*,*P* heterocycles. Through systematic increases in steric loading, the structures of these systems in the solid state can be tuned, leading to successive P–P bond lengthening and Sn–P contraction and, in the most encumbered case, to complete P-to-Sn transfer of the phosphinidene fragment. The resulting stannaphosphene features a polar Sn=P double bond as determined by structural and computational studies. The reversibility of phosphinidene transfer can be established by solution phase measurements and reactivity studies.

Yoshifuji’s synthesis of a stable diphosphene and West’s synthesis of tetramesityldisilene, both in 1981, did much to repudiate the so-called “double bond rule” and the implied limit on multiple bonding between heavier main group elements.^1^ Since these landmark reports, both homo- and heteronuclear multiple bonds featuring elements from groups 14–16 have attracted enormous interest.^2^ With respect to E^14^–E^15^ multiple bonds, the initial report of a phosphaalkyne RC≡P, was followed by the isolation of a range of phosphaalkenes.^3^ Sila-imines and heavier imine counterparts of germanium and tin have also been reported.^4^ In the realm of E^14^–P multiple bonding, silaphosphenes are well established, and their chemistry has recently been reviewed.^5^ However, examples of systems featuring Ge–P or Sn–P multiple bonding are scarce, with stannaphosphenes of type **I** being advanced in the literature, but with no structural data having been forthcoming (Scheme 1).^6^

**Scheme 1:**
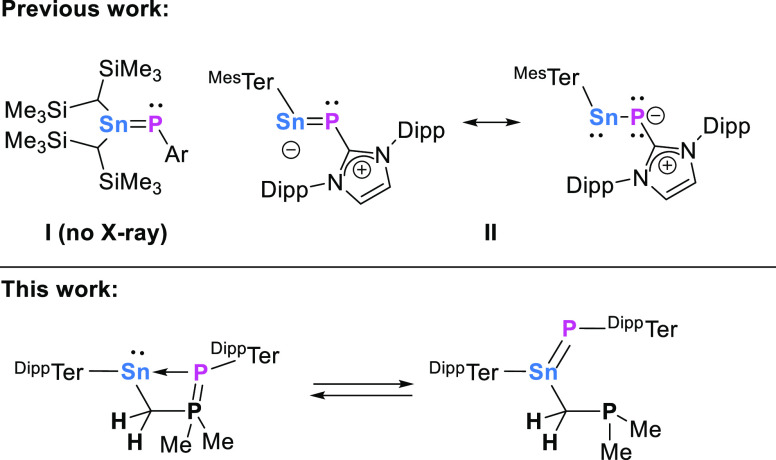
Compounds with Multiple Bonding between Tin and Phosphorus Including This Work

Inoue and Tan have recently reported the syntheses of germanium and tin compounds (e.g., **II**) that show short E^14^–P bonds and can be described in terms of a resonance contribution involving an E=P double bond.^7^ In this context, Inoue and co-workers were able to characterize a zwitterionic stannaphosphene by reacting compound **II** with B(C_6_F_5_)_3_.^7a^ Other notable achievements in heavier E^14^–E^15^ multiple bonding include a stibasilene and an arsagermene from Sekiguchi et al., although little reactivity was reported for either compound.^8^

Against this limited background of studies in tin phosphorus multiple bonding, we report the isolation of a crystalline stannaphosphene in the solid state. This compound additionally bears an intramolecular pendant phosphine donor and exists in solution as the corresponding four-membered *Sn*,*C*,*P*,*P* system featuring dative bonding between phosphorus and tin. Steric bulk at both the tin and phosphorus centers is found to be critical in terms of both structure and reactivity. As such, a series of solid-state structures is presented illustrating the stepwise phosphinidene transfer from P to Sn as a function of steric bulk, together with first insights into the reactivity of these compounds. The latter emphasizes the role of reversibility in phosphinidene transfer in the reactivity of the stannaphosphene.

Reactions of the heteroleptic terphenyl-/amido-stannylenes, ^R^TerSn{N(SiMe_3_)_2_} (**Sn1a,b**),^9,10^ with the phospha-Wittig reagents Me_3_PPR (**P1a**–**c**)^11^ in the temperature range from room temperature to 80 °C lead to the formation of the base-stabilized stannylenes (**Sn2a**–**d**), each of which features a four-membered *Sn*,*C*,*P*,*P* heterocycle in solution and can be isolated in good crystalline yield from aliphatic hydrocarbons (44–63%) (Scheme 2A).^12^ This mode of reactivity of phospha-Wittig reagents (formal methyl C–H activation) is to our knowledge unprecedented, with these reagents commonly acting as phosphinidene transfer reagents via release of PMe_3_.^11,13^ In this case, formation of the HN(SiMe_3_)_2_ coproduct is evident from its characteristic ^1^H NMR signal (δ_H_ = 0.10 ppm), and the accompanying tin-bound methylene group is revealed by a broad multiplet in the region 0–1 ppm. Retention of the P=P double bond from the phospha-Wittig precursor is also reflected in ^1^*J*_PP_ coupling constants for **Sn2a**–**d** in the range 300–350 Hz.

**Scheme 2:**
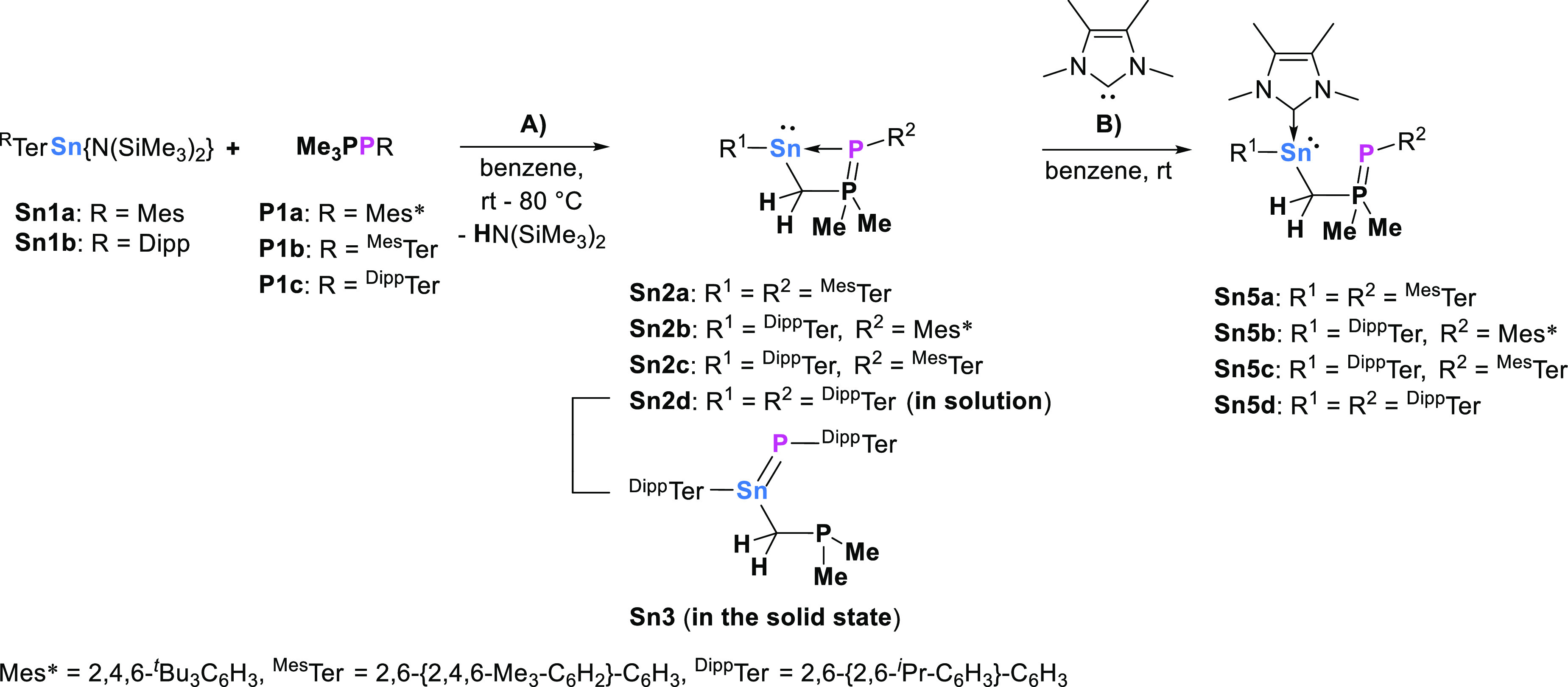
(A) Synthesis of ^R^TerSnCH_2_P(CH_3_)_2_=PR (**Sn2a**–**c**) and ^Dipp^TerSnCH_2_P(CH_3_)_2_=P^Dipp^Ter/^Dipp^TerSn[CH_2_P(CH_3_)_2_]=P^Dipp^Ter (**Sn2d**/**Sn3**) and (B) Synthesis of the NHC-Stabilized Stannylenes ^R^TerSn(IMe_4_)CH_2_P(CH_3_)_2_=PR (**Sn5a**–**d**)

Reaction monitoring by ^31^P{^1^H} NMR spectroscopy shows in each case the formation of two new doublet signals with tin satellites, shifted to lower-field compared to the free phospha-Wittig reagents (cf. Figure S15).^11^ In a comparative sense, multinuclear NMR data for **Sn2a**–**d** (Table S1) imply that the four systems possess near identical structures in solution, with the two ^31^P signals being found in the ranges −91.4 to −98.6 and 12.3 to 19.1 ppm (*J*_PP_ = 312.1–343.2 Hz), and the associated ^*n*^*J*_SnP_ couplings being measured at 539.5–656.8 (*n* = 1) and 231.3–307.3 Hz (*n* = 2). Crystalline material for each of **Sn2a**–**c** was obtained from aliphatic hydrocarbons, with crystallographic study in the cases of **Sn2b** (Figure S21) and **Sn2c** (Figure 1A) confirming the connectivity implied by solution-phase measurements.^14^

**Figure 1:**
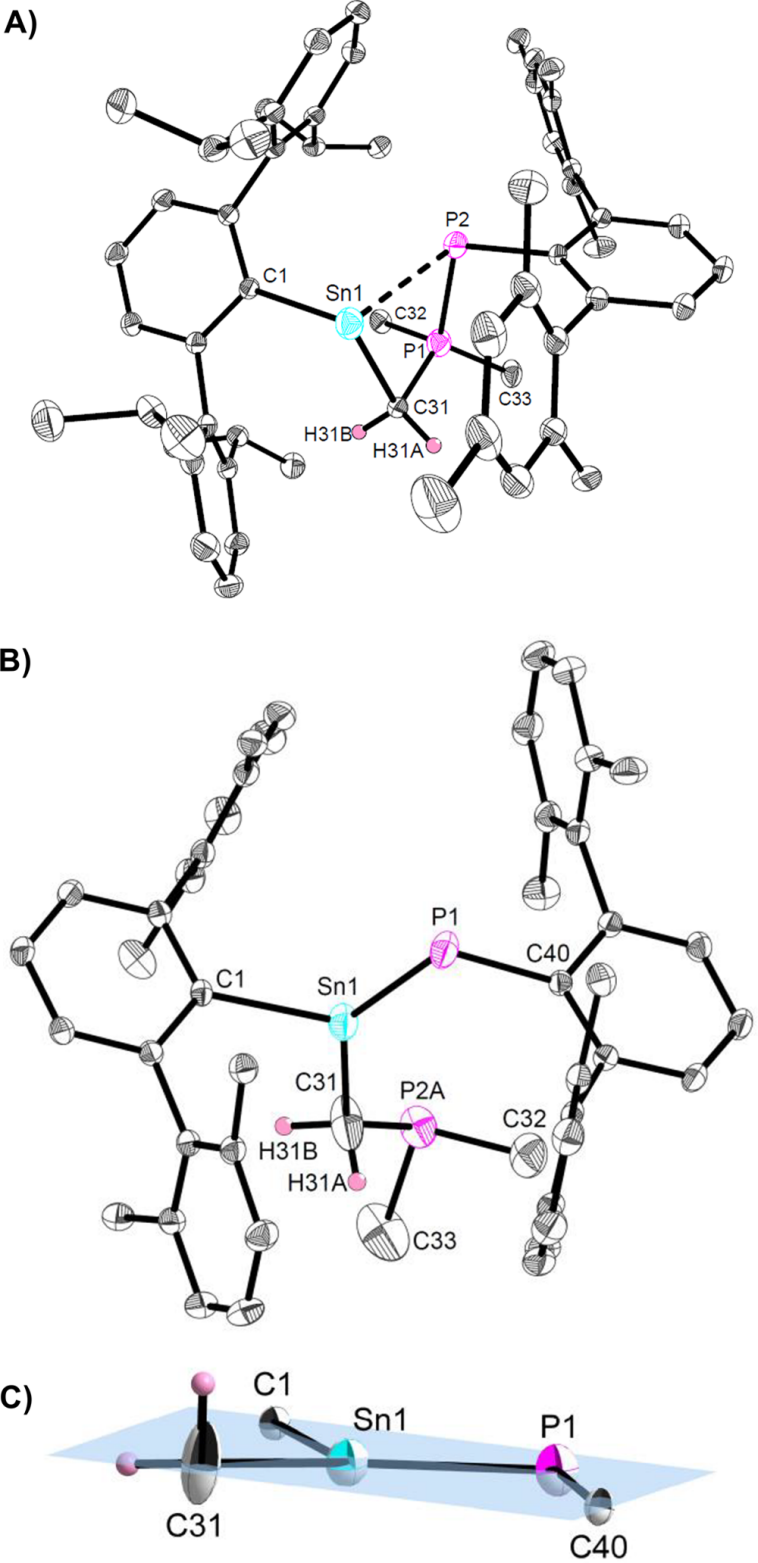
Molecular structures of (A) ^Dipp^TerSnCH_2_P(CH_3_)_2_=P^Mes^Ter (**Sn2c**) and (B) ^Dipp^TerSn[CH_2_P(CH_3_)_2_]=P^Dipp^Ter (**Sn3**) in the crystal. Thermal ellipsoids drawn at the 50% probability level and most hydrogen atoms omitted for clarity. Selected bond lengths (Å) and angles (deg): **Sn2c**, Sn1···P2 2.7727(7), Sn1–C1 2.275(3), Sn1–C31 2.307(3), P1–P2 2.1495(10), P1–C34 1.759(3), C1–Sn1–C31 97.90(10); **Sn3**, Sn1–P1 2.3425(4), Sn1–C1 2.1677(16), Sn1–C31 2.145(2), P2A–C31 1.771(3), C1–Sn1–P1 113.75(4), C1–Sn1–C31 107.45(8), C31–Sn1–P1 138.33(8). (C) Excerpt of the molecular structure of **Sn3** in the crystal showing the planarity of the central unit.

In the case of **Sn2c**, a dative interaction between P2 and Sn1 is suggested by a relatively long bond (2.7727(7) Å), which exceeds the molecular single bond radii of the respective atoms (2.51 Å)^15^ and which is aligned approximately perpendicular to the C1–Sn1–C31 stannylene plane. This dative interaction, and the associated four-membered ring, enforces a slightly obtuse Sn1–C31–P1 bond angle of 96.8(1)°. The P1–P2 bond length of 2.1495(10) Å is only slightly elongated compared to phospha-Wittig reagents (cf. Me_3_PP^Dipp^Ter, 2.0955(7) Å^11^), thus being characteristic of a double bond. Interestingly, the corresponding data for the slightly less sterically demanding PMes* system **Sn2b** (notably the Sn1···P2 separation of 2.8345(8) Å and Sn1–C31–P1 angle of 100.2(5)°) imply that the PAr unit becomes more closely linked to the tin center as the Ar group becomes more encumbered. Consistently, the single crystalline material obtained from the reaction of the most sterically demanding stannylene ^Dipp^TerSn{N(SiMe_3_)_2_} (**Sn1b**) with Me_3_PP^Dipp^Ter (**P1c**) is revealed to consist of the stannaphosphene ^Dipp^Ter[Me_2_PCH_2_]Sn=P^Dipp^Ter (**Sn3**; Figure 1, B)), rather than the base-stabilized stannylene **Sn2d** found in solution.

The Sn1–P1 bond in **Sn3** (at 2.3425(4) Å) is the shortest tin–phosphorus bond reported to date and in line with the respective covalent double bond radii (2.32 Å,^15^ cf. 2.3450(10) Å in ^Mes^TerSn(C_6_F_4_B(F)(C_6_F_5_)_2_)=P(IDipp)^7a^). Double bond character is further underlined by the sum of angles around the tin center (359.5°) rendering it trigonal planar (cf. Figure 1C). That there is no phosphorus–phosphorus bonding retained in **Sn3** is evident by the separation of >5.1 Å, which is markedly wider than both those in **Sn2b**/**Sn2c** and the sum of the single bond covalent radii of two phosphorus atoms (2.22 Å^15^).

Based on these structural data, the series of the structurally characterized compounds **Sn2b** (PMes*), **Sn2c** (P^Mes^Ter), and **Sn3** (P^Dipp^Ter), all bearing the ^Dipp^Ter moiety at tin, can be viewed as offering structural snapshots of phosphinidene transfer as a function of increasing steric bulk. The Sn–P distances contract from 2.8345(8) Å (**Sn2b**) to 2.7727(7) Å (**Sn2c**) to 2.3425(4) Å (**Sn3**), while the associated P–P distances change from 2.1323(12) Å (**Sn2b**) to 2.1495(10) Å (**Sn2c**) and >5.1 Å (**Sn3**) along the series. This seemingly counterintuitive contraction of the Sn–P(Ar) separation as the bulk of Ar increases appears to be related to the torsional alignment of the bulky P- and Sn-bound substituents. In **Sn3**, the positioning of the two ^Dipp^Ter groups is not constrained by the *Sn,C,P,P* heterocycle, and the central aryl rings of the two terphenyl ligands can align essentially coplanar to one another (torsion angle = 4.0°), positioned on either side of the Sn=P double bond (and orthogonal to it). By contrast, in both **Sn2b** and **Sn2c**, the analogous conformation is prevented due to the presence of the PMe_2_ group adjacent to the PAr unit within the four-membered ring, and the corresponding torsion angles are 74.5° (**Sn2b**) and 49.3° (**Sn2c**). As such, we hypothesize that (in the solid state at least) steric overloading in **Sn2d**/**Sn3** prompts P–P bond cleavage.

To obtain insight into the bonding in **Sn3** and the thermodynamics of its formation, quantum chemical calculations were performed at the M06-2X/def2-TZVP level. The HOMO and LUMO are best described as Sn=P π-bonding and π*-antibonding orbitals, respectively (Figure S70). Natural bonding orbital (NBO) analysis concurs, revealing the presence of both tin–phosphorus σ- and π-interactions (with occupancies of 1.80e and 1.91e, respectively). The atomic orbital contributions to the π bond have nearly pure p-orbital character with 78% being phosphorus-based. Consistent with this polarized π-bonding description, the Sn–P Wiberg bond index is calculated to be 1.63 and the natural charges are +1.89 (Sn) and −0.42 (P). The formation of the four-membered ring systems **Sn2b**–**Sn2d**, bearing ^Dipp^Ter moieties at tin was found to be exothermic and exergonic in all cases, with the most sterically demanding system being the least favorable (Δ*G* = −7.5 (**Sn2b**), −10.9 (**Sn2c**), −7.0 (**Sn2d**/**Sn3**) kcal mol^–1^). In the gas phase, the Sn=P form (**Sn3**) is calculated to be only slightly higher in energy (+5 kcal mol^–1^) than the corresponding four-membered ring system (**Sn2d**), in accordance with the phase-dependent structural properties observed experimentally for this system.

To probe the chemical reversibility of phosphinidene transfer, we examined the reactivity of **Sn2a**–**c** and **Sn2d**/**Sn3**, toward simple Lewis base coordination and E–H bond activation. All four systems were reacted with the *N*-heterocyclic carbene (NHC) 1,3,4,5-tetramethyl-2-imidazol-2-ylidene (IMe_4_) resulting in immediate (clean) formation of the NHC-stabilized stannylenes, ^R^TerSn(IMe_4_)CH_2_P(CH_3_)_2_=PR (**Sn5a**–**d**), thus effectively confirming reversibility of the phosphinidene transfer in the case of **Sn2d**/**Sn3** (Scheme 2B). In the case of **Sn5c**, the molecular structure in the solid state was verified crystallographically (Figure S51).^10,16^ As expected, due to the stronger donor capabilities of the IMe_4_ ligand, no significant tin–phosphorus interaction is retained in **Sn5c** (Sn1···P2 > 4.7 Å). The tin–carbene separation (Sn1–C31, 2.254(3) Å) is consistent with other stannylene carbene adducts (e.g., 2.287(3) Å in ((Me_3_Si)_3_Si)_2_Sn(IMe_4_)^17^), and the P1–P2 bond length (2.1022(13) Å) is indicative of a double bond, consistent with complete dissociation of the tethered phospha-Wittig functionality.

While this simple substitution chemistry is common to all four systems, the reactivity toward H_2_ and PhCCH reveals behavior that is unique to **Sn2d**/**Sn3**. **Sn2d**/**Sn3** reacts with H_2_ (>50 °C) to generate H_2_P^Dipp^Ter, with approximately 90% conversion being shown by ^31^P and ^31^P{^1^H} NMR spectroscopy over a period of 45 h (Scheme 3 and Figure S56).^10,18,19^

**Scheme 3:**
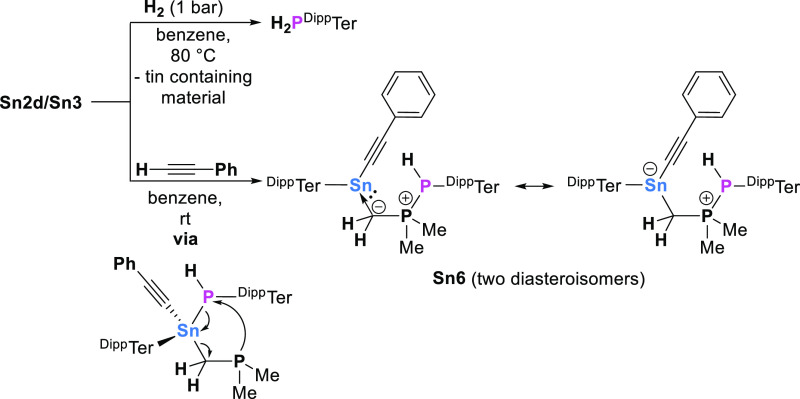
Reactivity of **Sn2d**/**Sn3** towards Dihydrogen and Phenylacetylene

In similar fashion, **Sn2d**/**Sn3** is uniquely reactive toward phenylacetylene. A rapid reaction is observed in benzene solution at room temperature, with conversion to two closely related species, each characterized by a pair of mutually coupled doublet signals in the ^31^P{^1^H} NMR spectrum (Figure S64). The associated ^119^Sn NMR signals are in the same region as the IMe_4_ stabilized stannylenes **Sn5a**–**d** (δ_Sn_ = −202.0 and −206.5 ppm, respectively). The structure determined crystallographically (Figure 2) shows that the product (^Dipp^TerSn(CCPh)CH_2_PP(H)^Dipp^Ter, **Sn6**) features a tin-bound acetylide moiety and that the pendant phospha-Wittig unit is protonated at P2. Elongation of the P1–P2 bond (2.1997(13) Å, cf. 2.1022(13) Å for **Sn5c**) is indicative of the reduction in bond order accompanying protonation. Moreover, the Sn1–C39 bond length (2.301(4) Å) is relatively long, being longer even than that involving the carbene donor in **Sn5c** (2.274(3) Å). This suggests a description as a dative interaction and that **Sn6** is best described as an acetylide-substituted stannylene, stabilized by an ylide-type L ligand. We assign the occurrence of two sets of diastereoisomers due to both **Sn1** and **P2** being stereogenic centers.^20^

**Figure 2:**
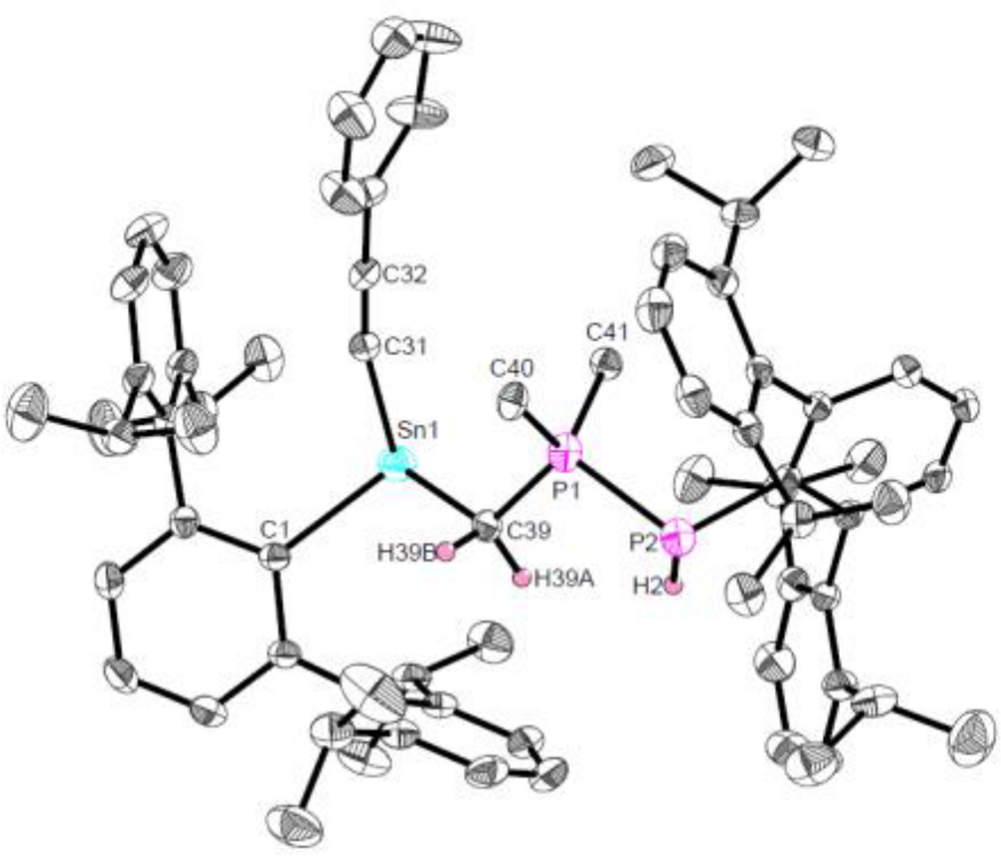
Molecular structure of ^Dipp^TerSn(CCPh)CH_2_P(CH_3_)_2_P(H)^Dipp^Ter (**Sn6**) in the crystal. Thermal ellipsoids are drawn at the 50% probability level (hydrogen atoms except H2, H39A, and H39B have been omitted for clarity). Selected bond lengths (Å) and angles (deg): Sn1–C1 2.250(4), Sn1–C31 2.203(4), Sn1–C39 2.301(4), P1–C39 1.756(4), P1–P2 2.1997(13), C1–Sn1–C31 103.39(14), C1–Sn1–C39 97.52(14).

By contrast, phenylacetylene does not react with the “simple” stannylene/phospha-Wittig adduct **Sn2b** under comparable conditions, implying that access to the stannaphosphine isomer (as in **Sn2d**/**Sn3**) is important in the cleavage of the C–H bond in PhCCH. By analogy with the (intramolecular) activation of C–H bonds by a stanna-imine,^21^ we propose that the first step in this chemistry involves cleavage of PhCCH into acetylide and protic components across the polarized Sn=P double bond in **Sn3**, with subsequent capture of the [PH^Dipp^Ter] fragment by the pendant phosphine arm (to generate the product **Sn6**) further emphasizing the reversible nature of tin–phosphorus interactions in this system.

In conclusion, we show (i) unusual reactivity of mixed *meta*-terphenyl- and amido-substituted stannylenes with phospha-Wittig reagents to give four-membered *Sn*,*C*,*P*,*P* systems, (ii) that phosphinidene transfer can be achieved through steric overloading, such that the doubly ^Dipp^Ter substituted system exists as the corresponding stannaphosphene in the solid state, (iii) reversibility in phosphinidene transfer for **Sn2d/Sn3** in reaction with IMe_4_, and (iv) reactivity studies toward H_2_ and HCCPh that imply the importance of access to the Sn=P bond for enabling small molecule activation.

## Supporting Information Available

Synthesis details, NMR spectra, and crystallographic and computational details (PDF)
Optimized Cartesian coordinates (XYZ)

## Supplementary Material

ja2c03302_si_001.pdf

ja2c03302_si_002.xyz

## Accession Codes

CCDC 2157153–2157161 contain the supplementary crystallographic data for this paper. These data can be obtained free of charge via www.ccdc.cam.ac.uk/data_request/cif, or by emailing data_request@ccdc.cam.ac.uk, or by contacting The Cambridge Crystallographic Data Centre, 12 Union Road, Cambridge CB2 1EZ, UK; fax: +44 1223 336033.
