# Supplementary material for: Structural Snapshots in Reversible Phosphinidene Transfer: Synthetic, Structural, and Reaction Chemistry of a Sn=P Double Bond
Source: J Am Chem Soc. 2022 May 10;144(20):8908–13. doi: 10.1021/jacs.2c03302 (PMC9136930; doi:10.1021/jacs.2c03302)
Supplement: Supplementary file 1 — ja2c03302_si_001.pdf [file ja2c03302_si_001.pdf]

Supporting Information

Structural Snapshots in Reversible Phosphinidene Transfer: Synthetic, Structural and Reaction Chemistry of a Sn=P Double Bond

Malte Fischer,*[a] Matthew M. D. Roy,[b] Lewis L. Wales,[a] Mathias A. Ellwanger,[a] Andreas Heilmann,[a] Simon Aldridge*[a]

[a] Dr. M. Fischer, Mr. L. L. Wales, Dr. M. A. Ellwanger, Mr. A. Heilmann, Prof. S. Aldridge
Inorganic Chemistry Laboratory, Department of Chemistry, University of Oxford, South Parks Road, Oxford, OX1 3QR
e-mail: malte.fischer@chem.ox.ac.uk; simon.aldridge@chem.ox.ac.uk

[b] Dr. M. M. D. Roy
Department of Chemistry, Catalysis Research Center and Institute for Silicon Chemistry, Technische Universität München, 85748 Garching bei München (Germany)

Table of Contents

General Considerations S2

Synthesis and Characterization of Compounds S3

Crystallographic Details S57

Computational Details S60

References S63

General Considerations

All manipulations were carried out using standard Schlenk line and glove box techniques under an atmosphere of dry argon or dinitrogen. Solvents were degassed by sparging with argon and dried by passing through a column of appropriate drying agent using a commercially available Braun SPS and stored over potassium mirror. Mes*PP$(CH_3)_3$ (**P1a**),[S1] ^{Mes}TerPP$(CH_3)_3$ (**P1b**),[S1] ^{Dipp}TerPP$(CH_3)_3$ (**P1c**),[S1] ^{Dipp}TerLi,[S2] and ^{Mes}TerLi (same procedure used as for ^{Dipp}TerLi starting with ^{Mes}TerI[S2]) were synthesized according to literature procedures. $SnCl_2$ and lithium hexamethyldisilazide [Li(hmds)] were commercially available, transferred into the glove box and used as received. IMe_4 was already present in the research group and used as available from the glove box. Phenylacetylene (HCCPh) was distilled over $CaCl_2$ and freeze-pump-thaw degassed three times prior to use. NMR spectra were measured in benzene-d_6 (C_6D_6) or toluene-d_8 (C_7D_8) which were dried over CaH_2, with the solvents being distilled under reduced pressure, degassed by three freeze-pump-thaw-cycles and stored under argon in Teflon valve ampoules. NMR samples were prepared under argon in 5 mm Wilmad 507-PP tubes fitted with J. Young Telfon valves. NMR spectra were measured on a Bruker Avance III HD Nanobay 400 MHz NMR spectrometer equipped with a 9.4 T magnet, Bruker Avance III 500 MHz NMR spectrometer equipped with a 11.75 T magnet or a Bruker Avance III NMR 500 MHz NMR spectrometer equipped with a 11.75 T magnet and a ^{13}C detect cryoprobe. ^{1}H and ^{13}C NMR spectra were referenced internally to residual protio-solvent (^{1}H) or solvent (^{13}C) resonances and are reported relative to tetramethylsilane (δ = 0 ppm). ^{31}P NMR spectra were referenced externally to an 85% solution of H_3PO_4 in water (δ = 0 ppm). ^{119}Sn NMR spectra were referenced with respect to $SnMe_4$ in C_6D_6. Chemical shifts are quoted in δ (ppm) and coupling constants in Hz. Elemental analyses were carried out by Elemental Microanalysis Ltd.

Synthesis and Characterization of Compounds

Synthesis of Sn(hmds)$_2$

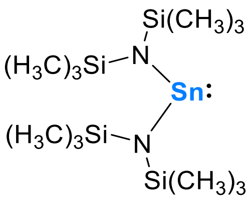

Sn(hmds)$_2$ was synthesized according to a modified literature procedure.[S3]
A Schlenk flask containing $SnCl_2$ (2.280 g, 12.03 mmol) and Li(hmds) (4.025 g, 24.05 mmol) was cooled to -78 °C followed by addition of ca. 50 mL of tetrahydrofuran. The resulting colourless solution was slowly warmed to room temperature which results in a colour change of the reaction mixture to a sli ght yellow. All volatiles were removed under vacuum and the residue was suspended in ca. 100 mL of toluene. Cannula filtration and removal of all volatiles yielded Sn(hmds) as a yellow solid. The following Figure S1 shows an exemplary ^{1}H NMR spectrum in C_6D_6 obtained following this procedure.

Yield: 4.453 g (10.13 mmol; 84%).
^{1}H NMR (400 MHz, C_6D_6, 298 K): δ = 0.29 (s, 36H, $Si(CH_3)_3$) ppm.

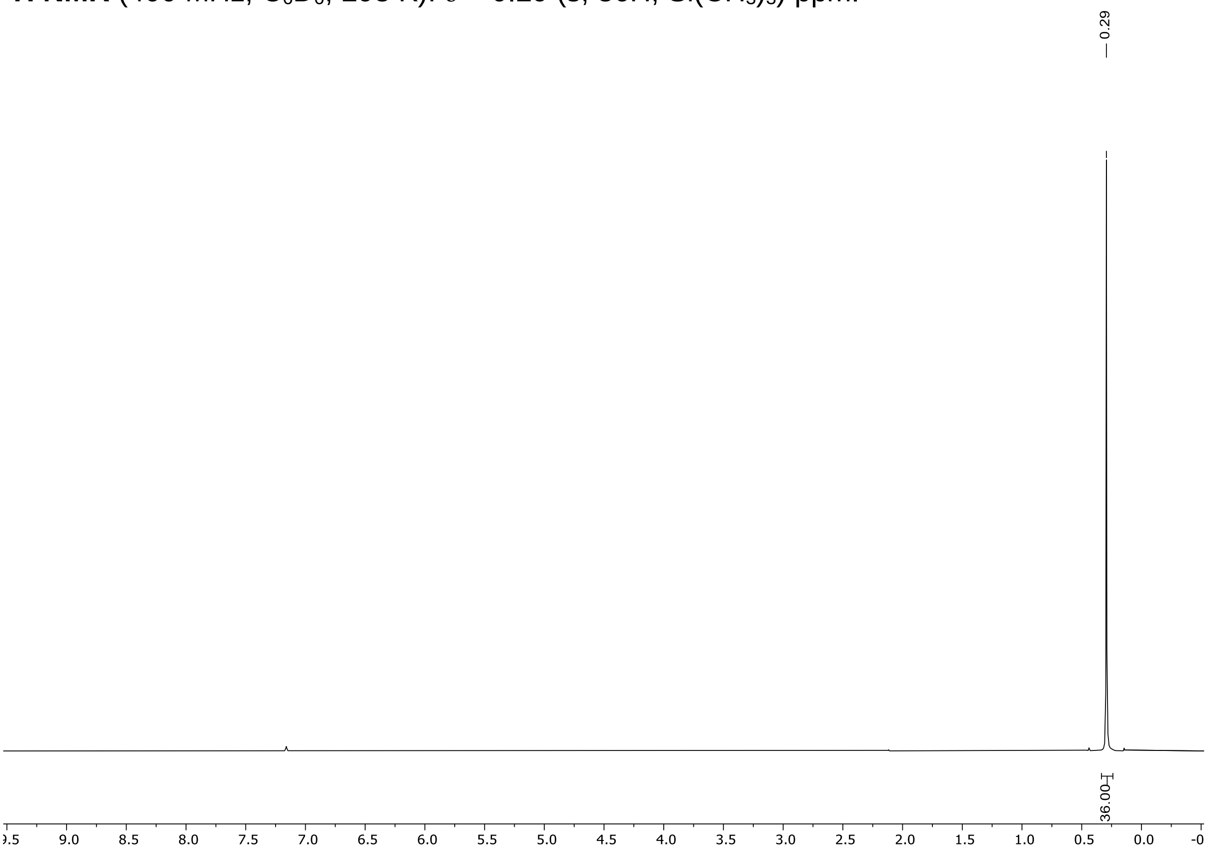

Figure S1. ^{1}H NMR spectrum of Sn(hmds)$_2$ (400 MHz, C_6D_6, 298 K).

Synthesis of new compounds

Synthesis of ^{Mes}TerSn(hmds) (Sn1a)

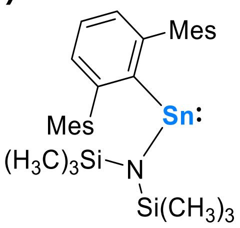

In a typical experiment ^{Mes}TerLi (1.000 g, 3.121 mmol) and $Sn(hmds)_2$ (1.372 g, 3.121 mmol) were suspended in 30 mL of toluene which results in a red suspension with a yellowish precipitate forming. The reaction mixture was stirred for 16 h at room temperature followed by cannula filtration. The solution was concentrated to incipient crystallization and stored at -30 °C to obtain ^{Mes}TerSn(hmds) (**Sn1a**) as a red crystalline solid. A second crop of crystals was obtained from the concentrated mother liquor at -30 °C. These crystals were suitable for single crystal X-ray diffraction.

Yield: 1.422 g (2.340 mmol; 75%).
^{1}H NMR (400 MHz, C_6D_6, 298 K): δ = -0.02 (s, 18H, $N(Si(CH_3)_3)_2$), 2.15 (s, 6H, CH_3), 2.32 (s, 12H, CH_3), 6.83 (s, 4H, CH_{Aryl}), 7.03-7.05 (m, 2H, CH_{Aryl}), 7.23-7.26 (m, 1H, CH_{Aryl}) ppm.
^{13}C{^{1}H} NMR (101 MHz, C_6D_6, 298 K): δ = 6.08 ($N(Si(CH_3)_3)_2$), 21.0 (CH_3), 22.1 (CH_3), 127.8 (CH_{Aryl})*, 129.9 (CH_{Aryl}), 130.4 (CH_{Aryl}), 136.8 ($C_{q,Aryl}$), 137.2 ($C_{q,Aryl}$), 137.8 ($C_{q,Aryl}$), 146.1 ($C_{q,Aryl}$), 184.5 ($C_{q,Aryl}Sn$) ppm.
* = overlap with C_6D_6 signal and assigned by ^{1}H/^{13}C HSQC/HMBC
EA: Anal. calcd. for $C_{30}H_{43}NSi_2Sn$: C, 60.81; H, 7.31; N, 2.36; Found: C, 60.91; H, 7.27; N, 2.34.

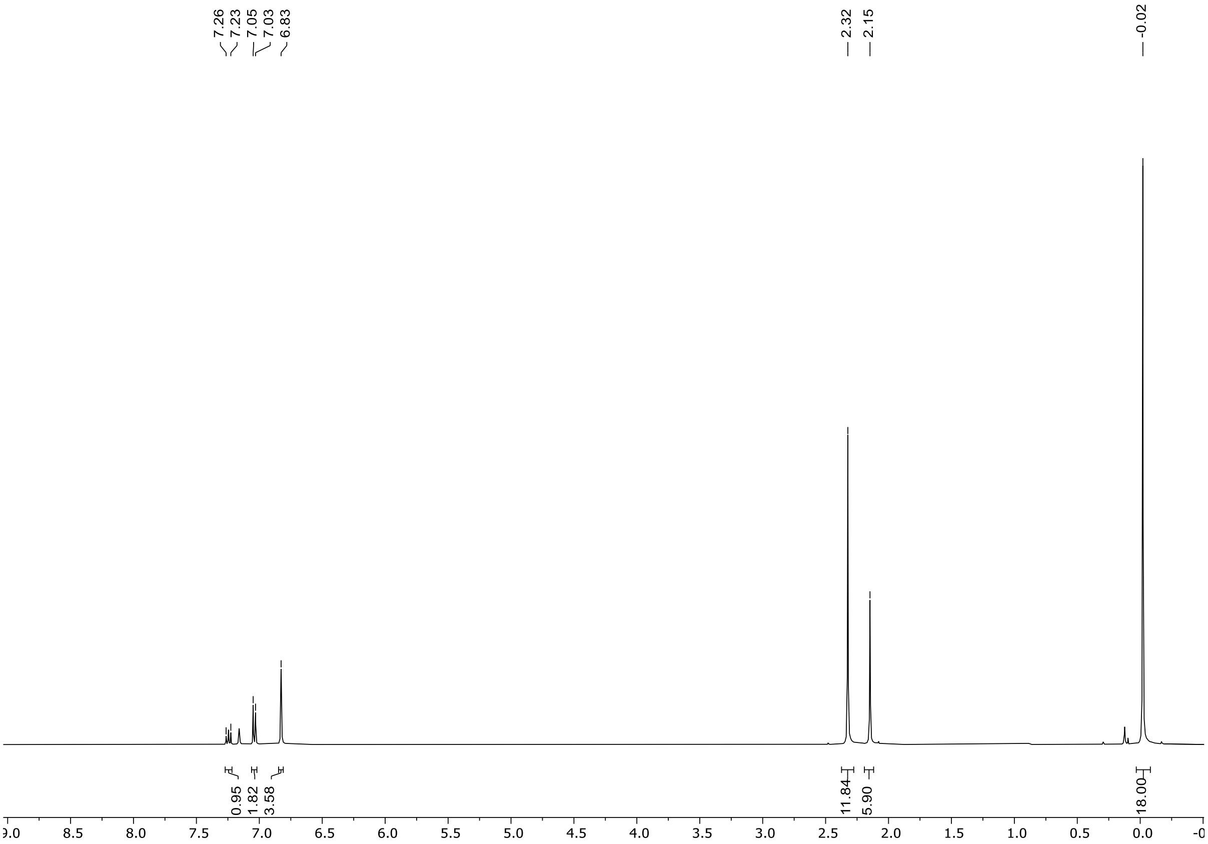

Figure S2: ^{1}H NMR spectrum of ^{Mes}TerSn(hmds) (**Sn1a**) (400 MHz, C_6D_6, 298 K).

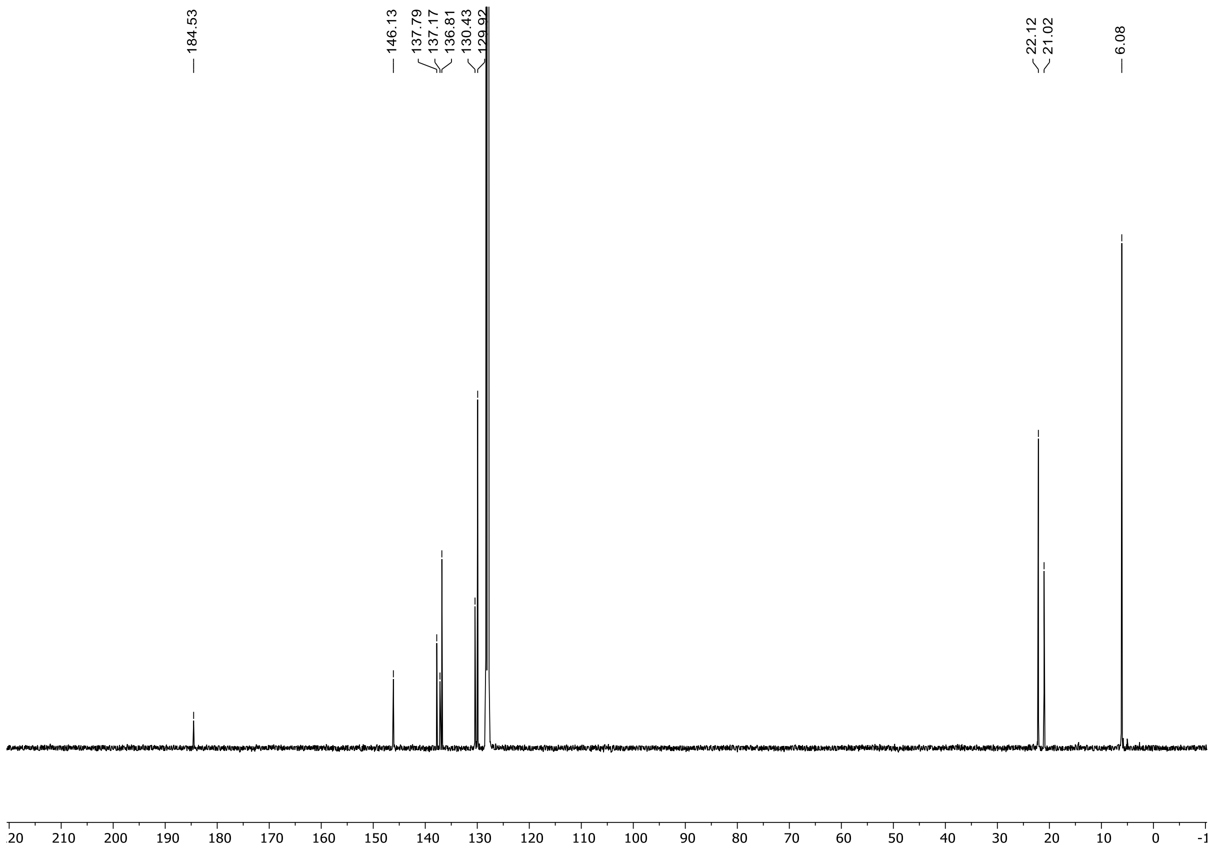

Figure S3: $^{13}C\{^{1}H\}$ NMR spectrum of ^{Mes}TerSn(hmds) (**Sn1a**) (101 MHz, C_6D_6, 298 K).

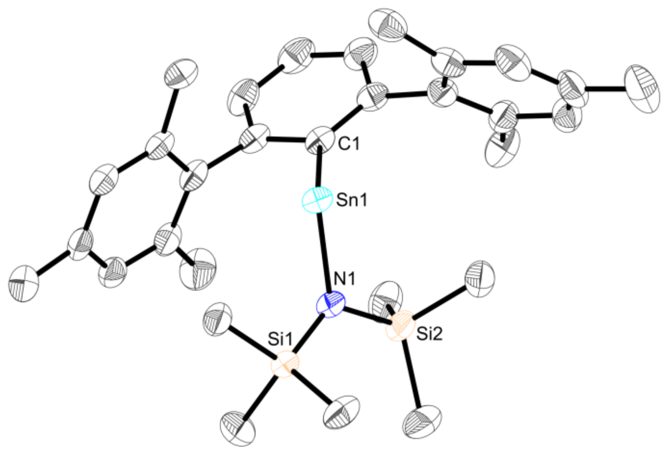

Figure S4. Molecular structure of ^{Mes}TerSn(hmds) (**Sn1a**) in the crystal. Thermal ellipsoids are drawn at the 50% probability level (hydrogen atoms have been omitted for clarity). Selected bond lengths (Å) and angles (deg): Sn1–N1 2.097(3), Sn1–C1 2.246(3), C1–Sn1–N2 104.13(13).

Synthesis of ^{Dipp}TerSn(hmds) (Sn1b)

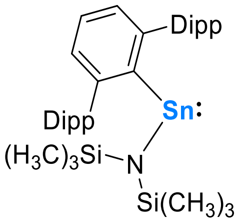

In a typical experiment ^{Dipp}TerLi (1.000 g, 2.472 mmol) and $Sn(hmds)_2$ (1.086 g, 2.472 mmol) were dissolved in ca. 50 mL of toluene. The resulting red suspension was stirred over night at room temperature followed by cannula filtration of the supernatant. All volatile components were removed under vacuum and the residue was suspended in ca. 30 mL of *n*-pentane followed by cannula filtration. The solution was concentrated to incipient crystallization and stored at -30 °C over night to give ^{Dipp}TerSn(hmds) (**Sn1b**) as a red solid. A second crop of crystals was obtained from the concentrated mother liquor at -30 °C.
Crystals suitable for single crystal X-ray diffraction were obtained from a saturated toluene solution of **Sn1b** at -30 °C.

Yield: 1.301 g (1.923 mmol; 78%).
^{1}H NMR (400 MHz, C_6D_6, 298 K): δ = 0.02 (s, 18H, $N(Si(CH_3)_3)_2$), 1.06 (d, $^3J_{H,H}$ = 6.7 Hz, 12H, $CH(CH_3)_2$), 1.38 (d, $^3J_{H,H}$ = 6.8 Hz, 12H, $CH(CH_3)_2$), 3.28 (hept, $^3J_{H,H}$ = 6.8 Hz, 4H, $CH(CH_3)_2$), 7.18-7.20 (m, 4H, CH_{Aryl}), 7.23-7.26 (m, 3H, CH_{Aryl}), 7.32-7.34 (m, 2H, CH_{Aryl}) ppm.
^{13}C{^{1}H} NMR (126 MHz, C_6D_6, 298 K): δ = 6.2 ($N(Si(CH_3)_3)_2$), 23.0 ($CH(\underline{C}H_3)_2$), 27.1 ($CH(\underline{C}H_3)_2$), 31.3 ($\underline{C}H(CH_3)_2$), 124.2 (CH_{Aryl}), 126.8 (CH_{Aryl}) 129.5 (CH_{Aryl}), 131.6 (CH_{Aryl}), 137.4 ($C_{q,Aryl}$), 144.8 ($C_{q,Aryl}$), 147.6 ($C_{q,Aryl}$), 185.3 ($C_{q,Aryl}$) ppm.
EA: Anal. calcd. for $C_{30}H_{43}NSi_2Sn$: C, 63.90; H, 8.19; N, 2.07; Found: C, 61.06; H, 8.23; N, 2.56.

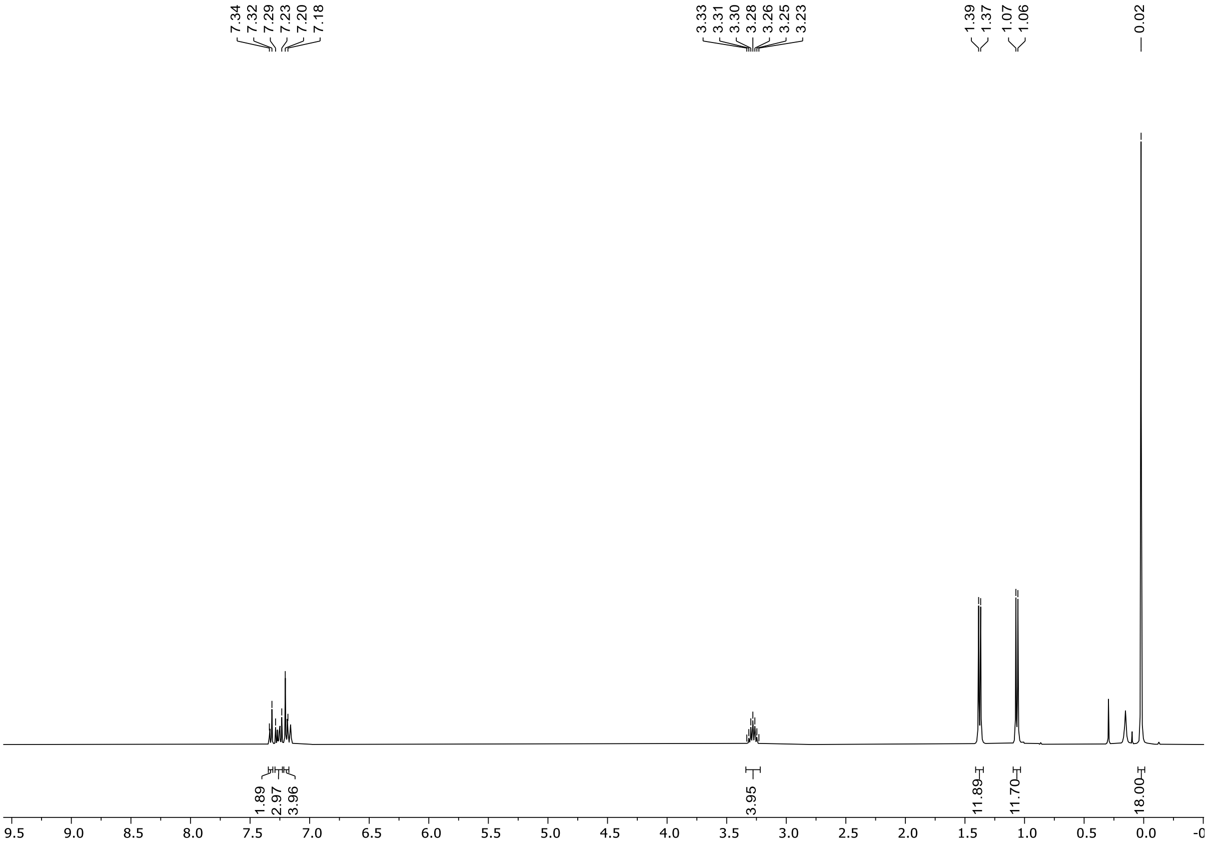

Figure S5. ^{1}H NMR spectrum of ^{Dipp}TerSn(hmds) (**Sn1b**) (400 MHz, C_6D_6, 298 K).

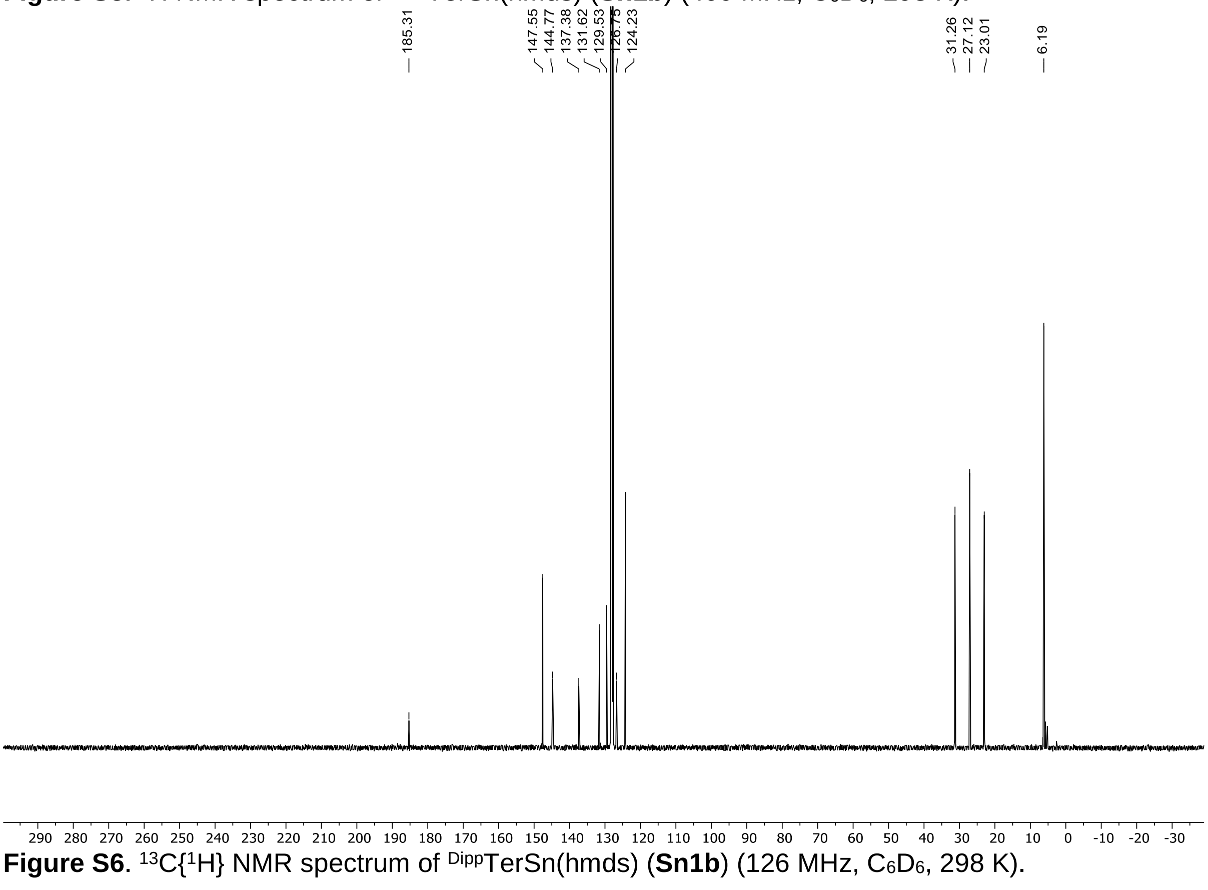

Figure S6. $^{13}C\{^{1}H\}$ NMR spectrum of ^{Dipp}TerSn(hmds) (**Sn1b**) (126 MHz, C_6D_6, 298 K).

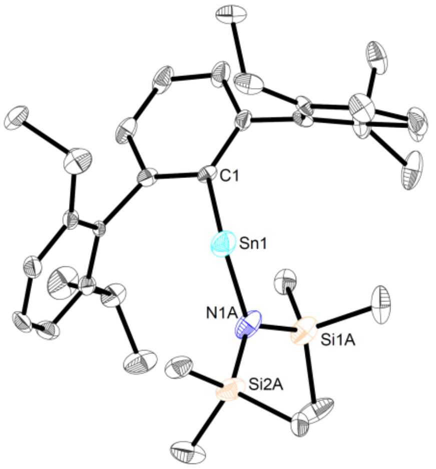

Figure S7. Molecular structure of DippTerSn(hmds) (**Sn1b**) in the crystal. Thermal ellipsoids are drawn at the 50% probability level (hydrogen atoms have been omitted for clarity). Selected bond lengths (Å) and angles (deg): Sn1–N1A 2.197(7), Sn1–C1 2.222(3), N1A–Sn1–C1 102.8(2).

Attempted reaction of ^{Mes}TerSn(hmds) (Sn1a) with Mes*PP(CH_3)$_3$ (P1a)

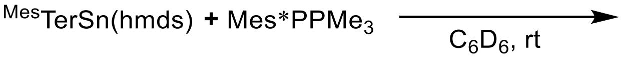

^{Mes}TerSn(hmds) (**Sn1a**) (0.050 g, 0.084 mmol) and Mes*PP(CH_3)$_3$ (**P1a**) (0.030 g, 0.084 mmol) were dissolved in 0.6 mL of C_6D_6 and the reaction mixture was monitored over time by $^{31}P\{^1H\}$ NMR spectroscopy (Figure S8). Analysis of the data revealed that after 16 h at room temperature no reaction could be observed. After heating the reaction mixture to 80 °C for a prolonged time mainly the known byproducts of heating Mes*PP(CH_3)$_3$ were observed (Mes*PPMes* : δ = 495.2 ppm, P(CH3)3: δ = -62.3 ppm, 3,3-dimethyl-5,7-di-^{t}butyl-phosphaindane: δ = -79.7 ppm). Additional heating at 80 °C over the weekend lead to no phospha-Wittig reagent being left.

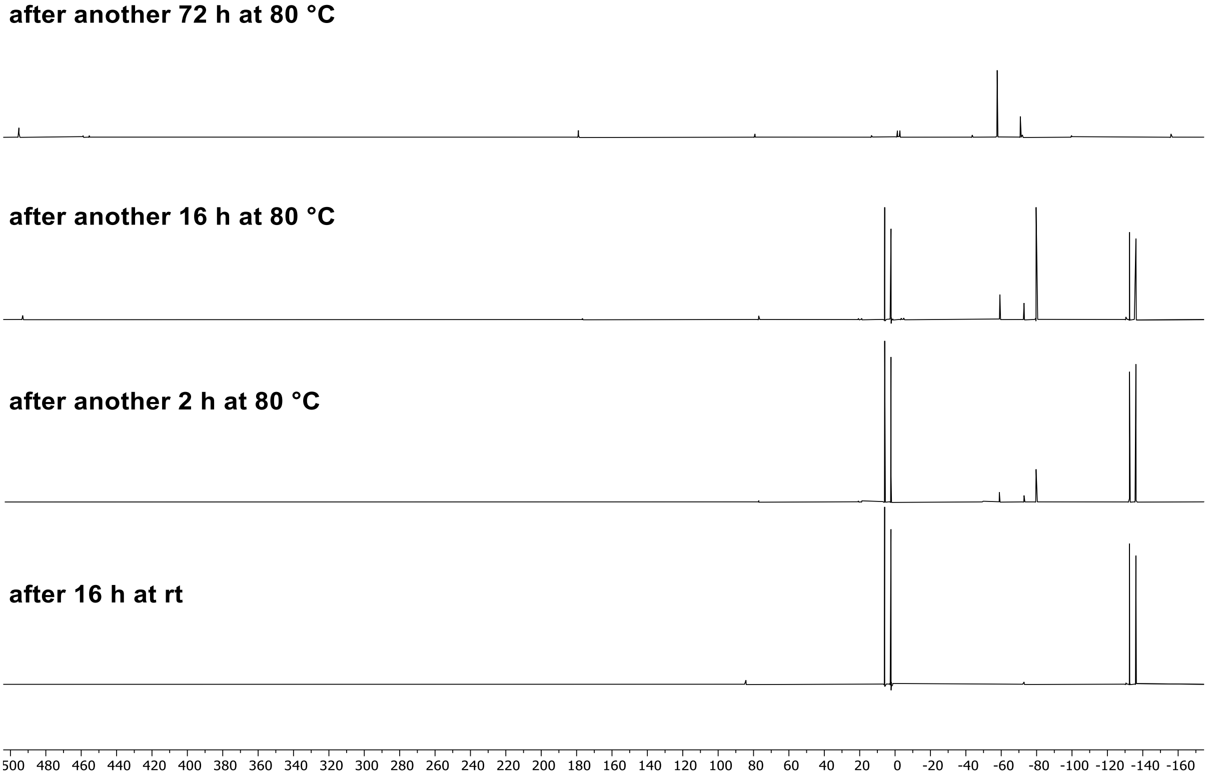

Figure S8. Monitoring of the reaction of ^{Mes}TerSn(hmds) (**Sn1a**) with Mes*PP(CH_3)$_3$ (**P1a**) via $^{31}P\{^1H\}$ NMR spectroscopy (161 MHz, C_6D_6, 298 K).

Attempted reaction of ^{Mes}TerSn(hmds) (Sn1a) with ^{Dipp}TerPP(CH_3)$_3$ (P1c)

$$^{Mes}\text{TerSn(hmds)} + {}^{Dipp}\text{TerPPMe}_3 \xrightarrow[C_6D_6,\ rt]{}$$

^{Mes}TerSn(hmds) (**Sn1a**) (0.030 g, 0.051 mmol) and ^{Dipp}TerPP(CH_3)$_3$ (**P1c**) (0.026 g, 0.051 mmol) were dissolved in 0.6 mL of C_6D_6 and the reaction mixture was monitored over time by ^{31}P{^{1}H} NMR spectroscopy (Figure S9). Analysis of the data revealed that even after 72 h at 80 °C no reaction could be observed.

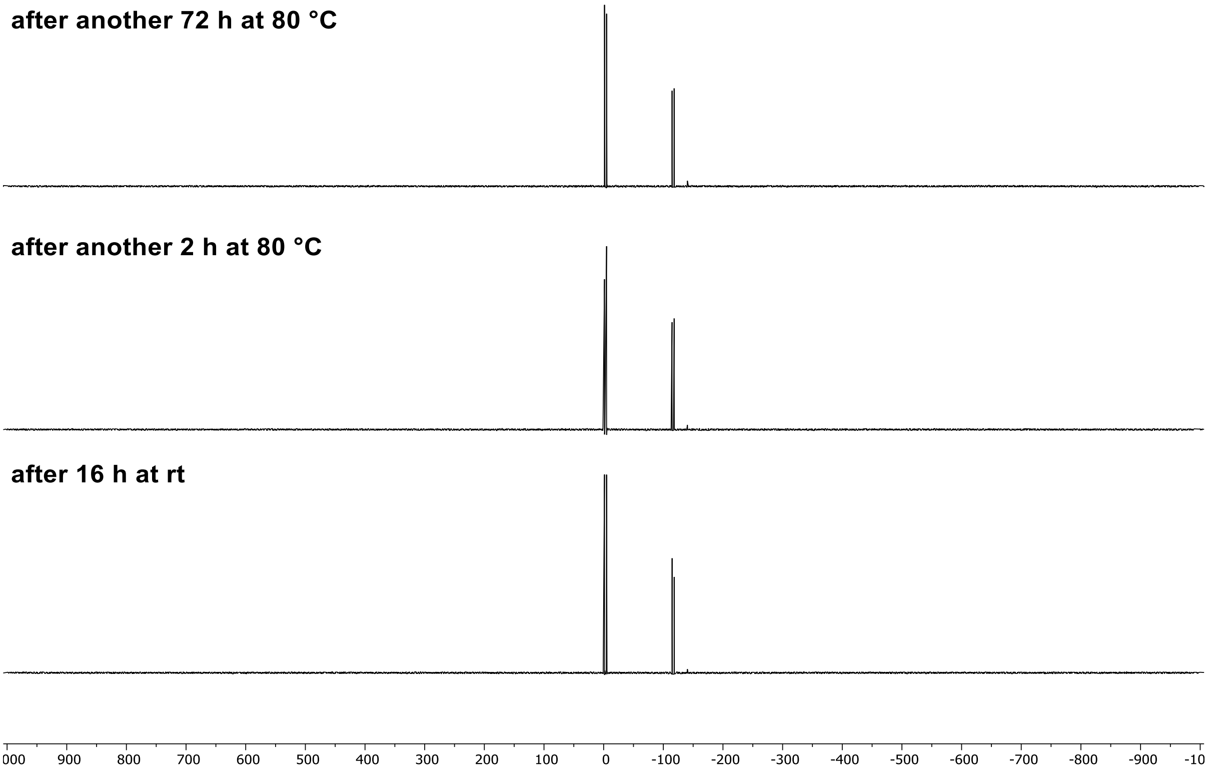

Figure S9. Monitoring of the reaction of ^{Mes}TerSn(hmds) (**Sn1a**) with ^{Dipp}TerPP(CH_3)$_3$ (**P1c**) via ^{31}P{^{1}H} NMR spectroscopy (161 MHz, C_6D_6, 298 K).

Synthesis of $^{Mes}TerSnCH_2P(CH_3)_2{=}P^{Mes}Ter$ (Sn2a)

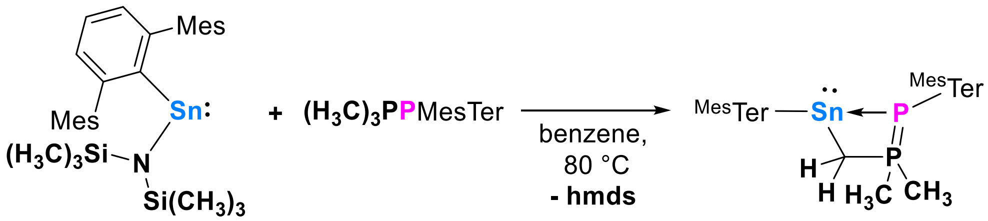

$^{Mes}TerSn(hmds)$ (**Sn1a**) (0.030 g, 0.071 mmol) and $^{Mes}TerPP(CH_3)_3$ (**P1b**) (0.042 g, 0.071 mmol) were dissolved in 0.5 ml of C_6D_6 and the reaction mixture was heated to 80 °C for 12 h, resulting in clean formation of $^{Mes}TerSnCH_2P(CH_3)_2{=}P^{Mes}Ter$ (**Sn2a**) and hmds. All volatile components were removed under vacuum and the remaining yellow solid was recrystallized from a saturated *n*-hexane solution at -30 °C to give $^{Mes}TerSnCH_2P(CH_3)_2{=}P^{Mes}Ter$ (**Sn2a**) as a microcrystalline yellow solid after removal of the supernatant and subsequent drying under vacuum.

Yield: 0.026 g (0.031 mmol; 44%).
^{1}H NMR (400 MHz, C_6D_6, 298 K): δ = 0.78-092 (m(br), 2H, CH_2), 1.76 (m(br), 6H, $P(CH_3)_2$), 2.09/2.34 (m, 36H, CH_3), 6.74-6.76 (m, 2H, CH_{Aryl}), 6.78-6.95 (m, 11H, CH_{Aryl}), 7.20-7.23 (m, 1H, CH_{Aryl}) ppm.
^{13}C{^{1}H} NMR (126 MHz, C_6D_6, 298 K): δ = 6.8 (dd, $^{1}J_{P,C}$ = 35.3 Hz, $^{2}J_{P,C}$ = 21.1 Hz, CH_2), 21.0 (CH_3), 21.1 (CH_3), 21.6 (CH_3), 21.7 (CH_3), 21.9 (br, $P(CH_3)_2$), 126.5 (CH_{Aryl}), 126.6 (d, $J_{P,C}$ = 2.7 Hz, CH_{Aryl}), 127.8 (CH_{Aryl})*, 128.1 (CH_{Aryl}), 128.5 (CH_{Aryl}), 129.8 (d, $J_{P,C}$ = 2.7 Hz, CH_{Aryl}), 135.4 (dd, $J_{P,C}$ = 56.6 Hz, $J_{P,C}$ = 9.3 Hz, $C_{q,Aryl}$), 135.3 ($C_{q,Aryl}$), 136.0 ($C_{q,Aryl}$), 136.1 (br, $C_{q,Aryl}$), 136.3 (br, $C_{q,Aryl}$), 140.4 ($C_{q,Aryl}$), 141.3 ($C_{q,Aryl}$), 147.5-147.6 (m, $C_{q,Aryl}$), 147.9 ($C_{q,Aryl}$), 169.0 (dd, $J_{P,C}$ = 8.7 Hz, $J_{P,C}$ = 4.4 Hz, $C_{q,Aryl}$) ppm.
* = overlap with C_6D_6 signal and assigned by ^{1}H/^{13}C HSQC/HMBC
^{31}P{^{1}H} NMR (161 MHz, C_6D_6, 298 K): δ = -91.4 (d, $^{1}J_{P,P}$ = 338.3 Hz, Sn satellites: $J_{119Sn,P}$ = 616.8 Hz, $J_{117Sn,P}$ = 589.1 Hz, $P^{Mes}Ter$), 19.1 (d, $^{1}J_{P,P}$ = 338.2 Hz, $J_{119Sn,P}$ = 230.2 Hz, $J_{117Sn,P}$ = 220.9 Hz, $H_2CP(CH_3)_2$) ppm.
^{31}P NMR (161 MHz, C_6D_6, 298 K): δ = -91.4 (dm, $^{1}J_{P,P}$ = 338.3 Hz, $P^{Mes}Ter$), 19.1 (dhept, $^{1}J_{P,P}$ = 338.2 Hz, $J_{P,H}$ = 12.1 Hz, $H_2CP(CH_3)_2$) ppm.
^{119}Sn{^{1}H} NMR (149 MHz, C_6D_6, 298 K): δ = 128.9 (dd, $J_{119Sn,P}$ = 617.8 Hz, $J_{119Sn,P}$ = 231.3 Hz) ppm.
EA: Anal. calcd. for $C_{51}H_{58}P_2Sn$: C, 71.92; H, 6.86; Found: C, 71.48; H, 7.50.

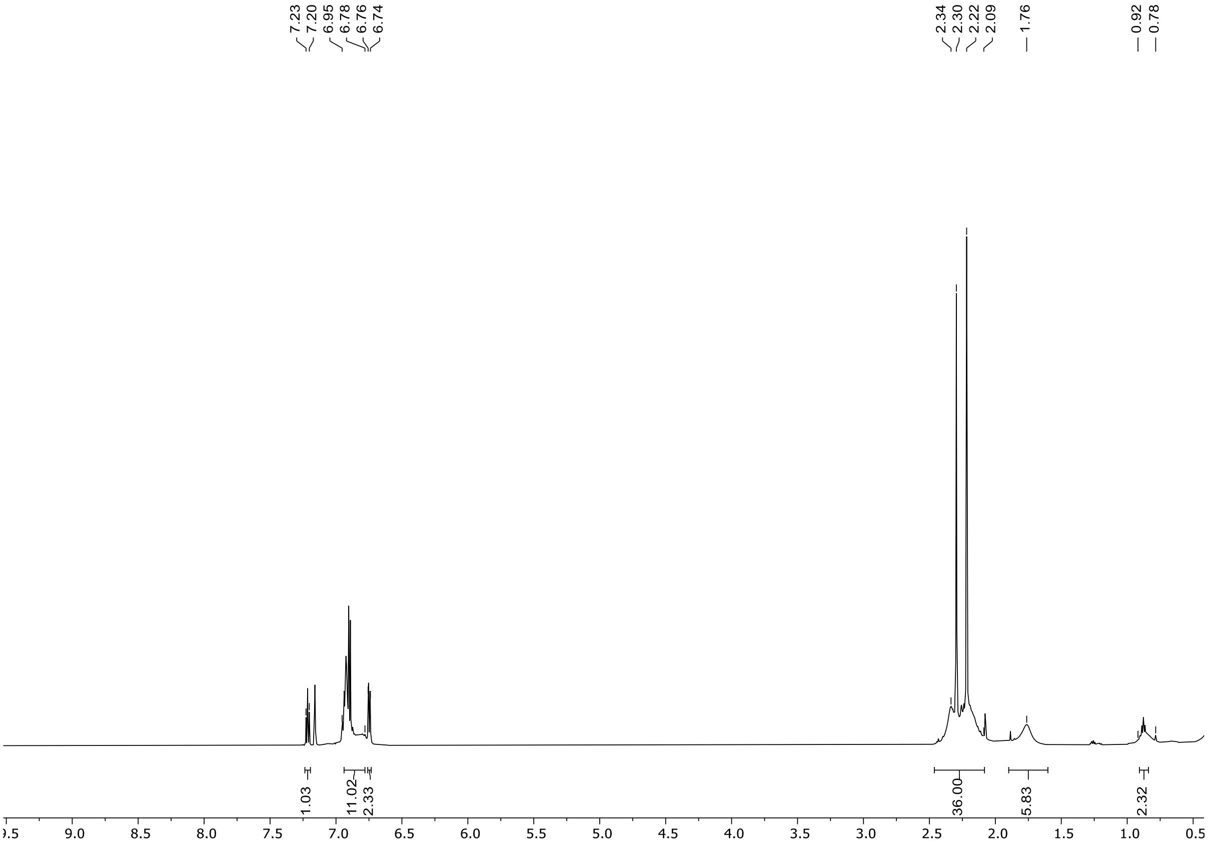

Figure S10. ^{1}H NMR spectrum of $^{Mes}TerSnCH_2P(CH_3)_2{=}P^{Mes}Ter$ (**Sn2a**) (400 MHz, C_6D_6, 298 K).

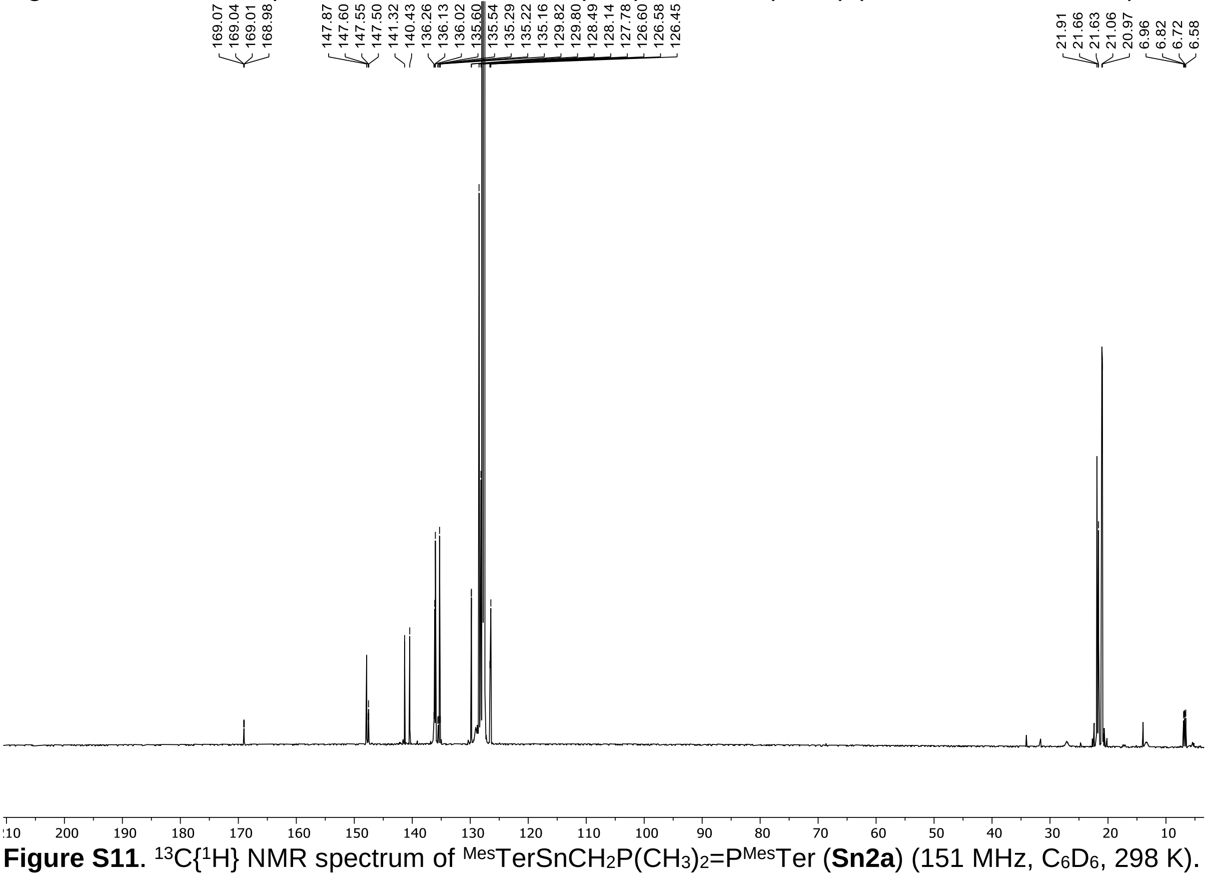

Figure S11. $^{13}C\{^{1}H\}$ NMR spectrum of $^{Mes}TerSnCH_2P(CH_3)_2{=}P^{Mes}Ter$ (**Sn2a**) (151 MHz, C_6D_6, 298 K).

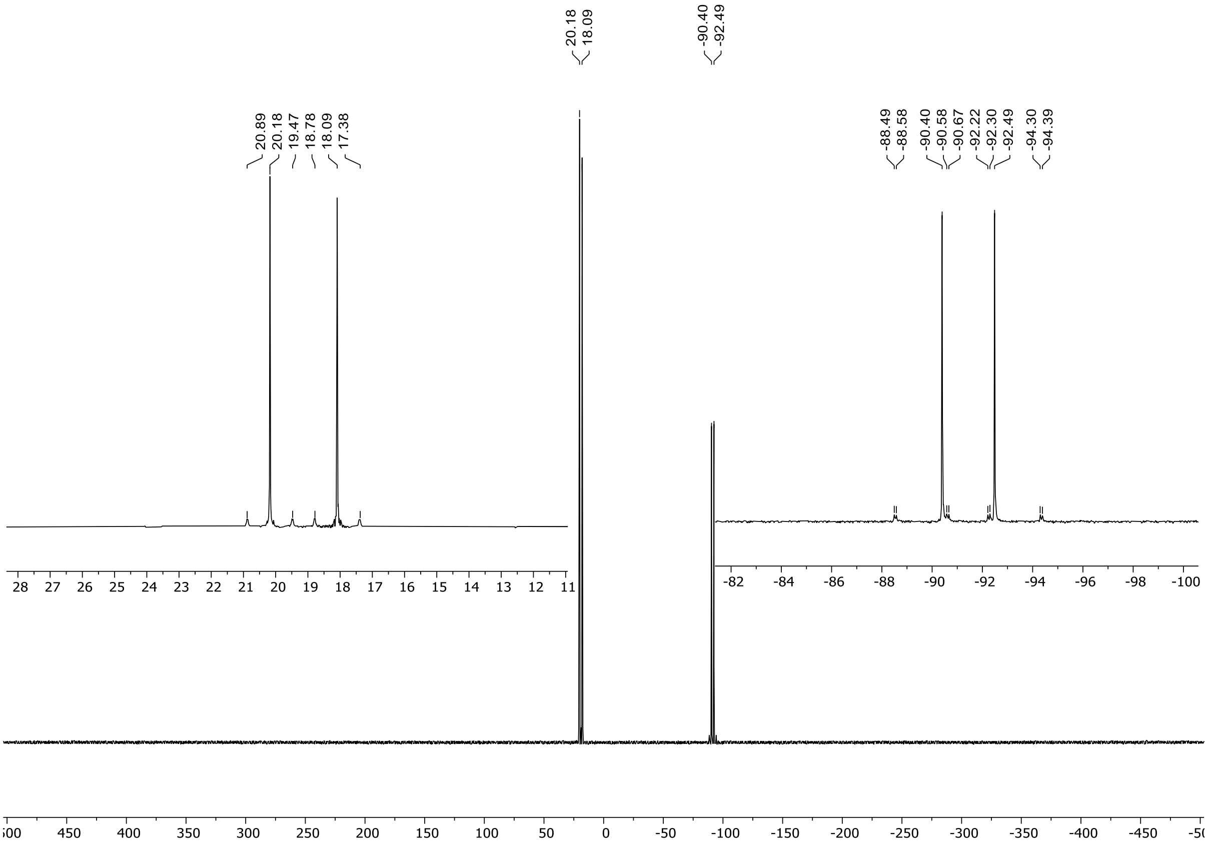

Figure S12. $^{31}P\{^{1}H\}$ NMR spectrum of ^{Mes}TerSnCH$_2$P(CH$_3$)$_2$=P^{Mes}Ter (**Sn2a**) (161 MHz, C_6D_6, 298 K).

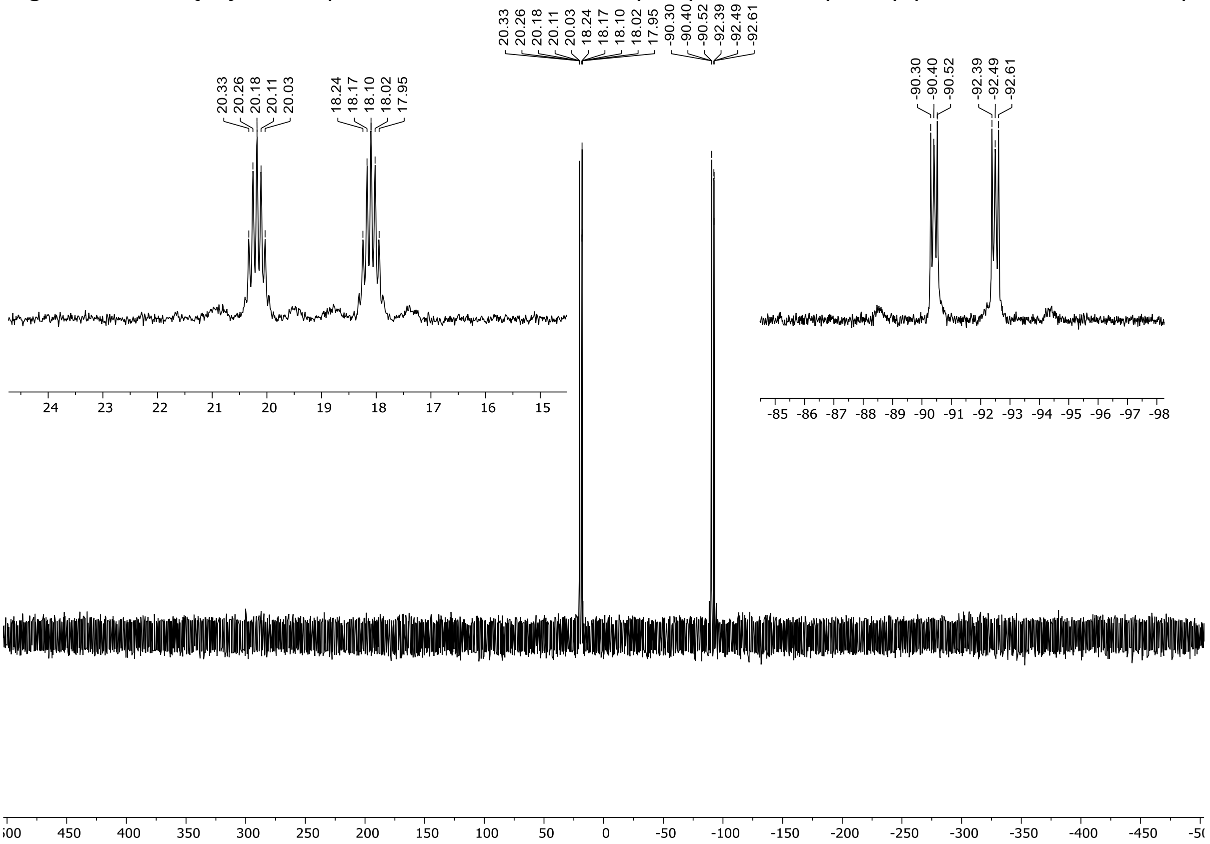

Figure S13. ^{31}P NMR spectrum of ^{Mes}TerSnCH$_2$P(CH$_3$)$_2$=P^{Mes}Ter (**Sn2a**) (161 MHz, C_6D_6, 298 K).

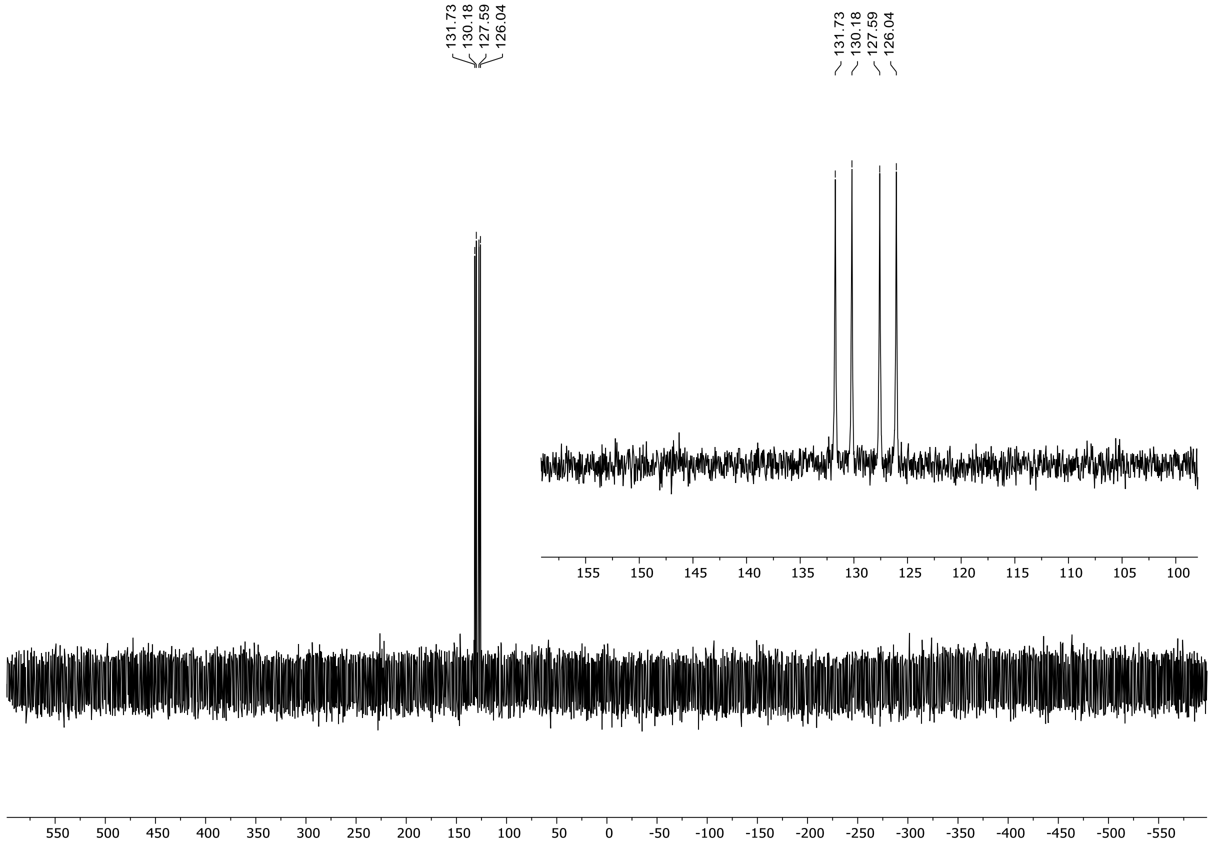

Figure S14. $^{119}Sn\{^1H\}$ NMR spectrum of $^{Mes}TerSnCH_2P(CH_3)_2{=}P^{Mes}Ter$ (**Sn2a**) (149 MHz, C_6D_6, 298 K).

Synthesis of $^{Dipp}TerSnCH_2P(CH_3)_2{=}PMes^*$ (Sn2b)

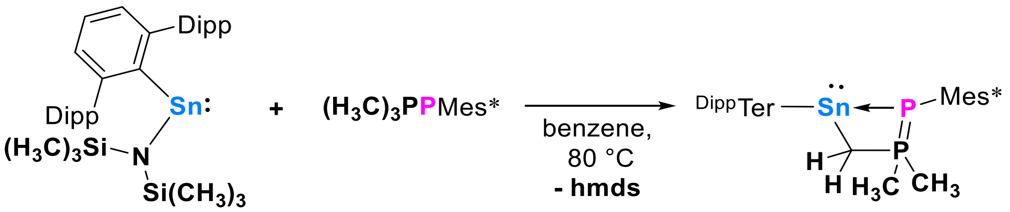

In an initial NMR scale experiment ^{Dipp}TerSn(hmds) (**Sn1b**) (0.050 g, 0.066 mmol) and Mes*PP$(CH_3)_3$ (**P1a**) (0.023 g, 0.066 mmol) were dissolved in 0.5 mL of C_6D_6 and the reaction progress was monitored by $^{31}P\{^1H\}$ NMR spectroscopy (Figure S15). No reaction could be observed after 2 h at room temperature. After heating the reaction mixture to 80 °C for 3 h two new doublet signals with tin satellites could be observed along with small amounts of the known byproducts of heating a solution of Mes*PP$(CH_3)_3$ (Mes*PPMes*, PMe_3, 3,3-dimethyl-5,7-di-^{t}butyl-phosphaindane). After additional 40 h at 80 °C Mes*PP$(CH_3)_3$ (**P1a**) was completely consumed. (**Note**: Although a 1:1 stoichiometry was applied, some unreacted ^{Dipp}TerSn(hmds) (**Sn1b**) remained in solution due to the formed byproducts. Therefore a 1:1.2 stoichiometry was used for all subsequent experiments.) Removal of all volatile components under vacuum, addition of ca. 1 mL of an aliphatic hydrocarbon (*n*-pentane, *n*-hexane or *n*-heptane were used during the course of this study), subsequent filtration and storage of the saturated solutions at -30 °C yielded $^{Dipp}TerSnCH_2P(CH_3)_2{=}PMes^*$ (**Sn2b**) as an orange crystalline solid.
Crystals suitable for single crystal X-ray diffraction were obtained from a saturated solution of **Sn2b** in *n*-pentane.

Yield: 0.034 g (0.039 mmol; 59%).
^{1}H NMR (400 MHz, C_6D_6, 298 K): δ = 0.25-0.31 (m(br), 2H, CH_2), 0.53 (d, $^2J_{P,H}$ = 12.3 Hz, 6H, $P(CH_3)_2$), 1.18-1.20 (m, 21H, $CH(CH_3)_2$, $C_q(CH_3)_3$), 1.52 (s, 18H, $C_q(CH_3)_3$), 1.57 (d, $^3J_{H,H}$ = 6.8 Hz, 12H, $CH(CH_3)_2$), 3.31 (hept, $^3J_{H,H}$ = 7.0 Hz, 4H, $CH(CH_3)_2$), 7.20-7.24 (m, 6H, CH_{Aryl}), 7.26-7.27 (m, 3H, CH_{Aryl}), 7.28-7.32 (m, 2H, CH_{Aryl}) ppm.
$^{13}C\{^1H\}$ NMR (126 MHz, C_6D_6, 298 K): δ = 10.2-10.6 (m, CH_2), 24.0 ($CH(\underline{C}H_3)_2$), 26.1 ($CH(\underline{C}H_3)_2$), 31.0 (d, $^1J_{P,C}$ = 46.3 Hz, $P(CH_3)_2$), 31.2 ($\underline{C}H(CH_3)_2$), 31.5 ($C_q(\underline{C}H_3)_3$), 34.5 ($\underline{C}_q(CH_3)_3$), 35.0 ($C_q(\underline{C}H_3)_3$), 39.1 ($\underline{C}_q(CH_3)_3$), 120.5 (d, $J_{P,C}$ = 4.4 Hz, CH_{Aryl}), 123.3 (CH_{Aryl}), 125.9 (CH_{Aryl}), 127.9 (CH_{Aryl})*, 129.6 (CH_{Aryl}), 131.2 (dd, $J_{P,C}$ = 63.3 Hz, $J_{P,C}$ = 9.8 Hz, $C_{q,Aryl}$), 142.8 ($C_{q,Aryl}$), 147.0 ($C_{q,Aryl}$), 147.7 ($C_{q,Aryl}$), 148.0 (d, $J_{P,C}$ = 63.3 Hz, $C_{q,Aryl}$), 159.0 (s(br), $C_{q,Aryl}$), 170.6 (d, $J_{P,C}$ = 10.3 Hz, $C_{q,Aryl}$) ppm.
* = overlap with C_6D_6 signal and assigned by $^1H/^{13}C$ HSQC/HMBC
$^{31}P\{^1H\}$ NMR (161 MHz, C_6D_6, 298 K): δ = -93.4 (d, $^1J_{P,P}$ = 318.7 Hz, Sn satellites: $J_{119Sn,P}$ = 655.7 Hz, $J_{117Sn,P}$ = overlapped, PMes*), 19.0 (d, $^1J_{P,P}$ = 318.7 Hz, $J_{119Sn,P}$ = 245.6 Hz, $J_{117Sn,P}$ = 233.8 Hz, $H_2CP(CH_3)_2$) ppm.
^{31}P NMR (161 MHz, C_6D_6, 298 K): δ = -93.4 (dt, $^1J_{P,P}$ = 318.0 Hz, $J_{P,H}$ = 25.4 Hz, PMes*), 19.0 (dhept, $^1J_{P,P}$ = 318.3 Hz, $J_{P,H}$ = 11.7 Hz, $H_2CP(CH_3)_2$) ppm.
$^{119}Sn\{^1H\}$ NMR (149 MHz, C_6D_6, 298 K): δ = 311.9 (dd, $J_{119Sn,P}$ = 656.8 Hz, $J_{119Sn,P}$ = 249.7 Hz) ppm.
EA: Anal. calcd. for $C_{51}H_{74}P_2Sn$: C, 70.59; H, 8.60; Found: C, 69.59; H, 8.78.

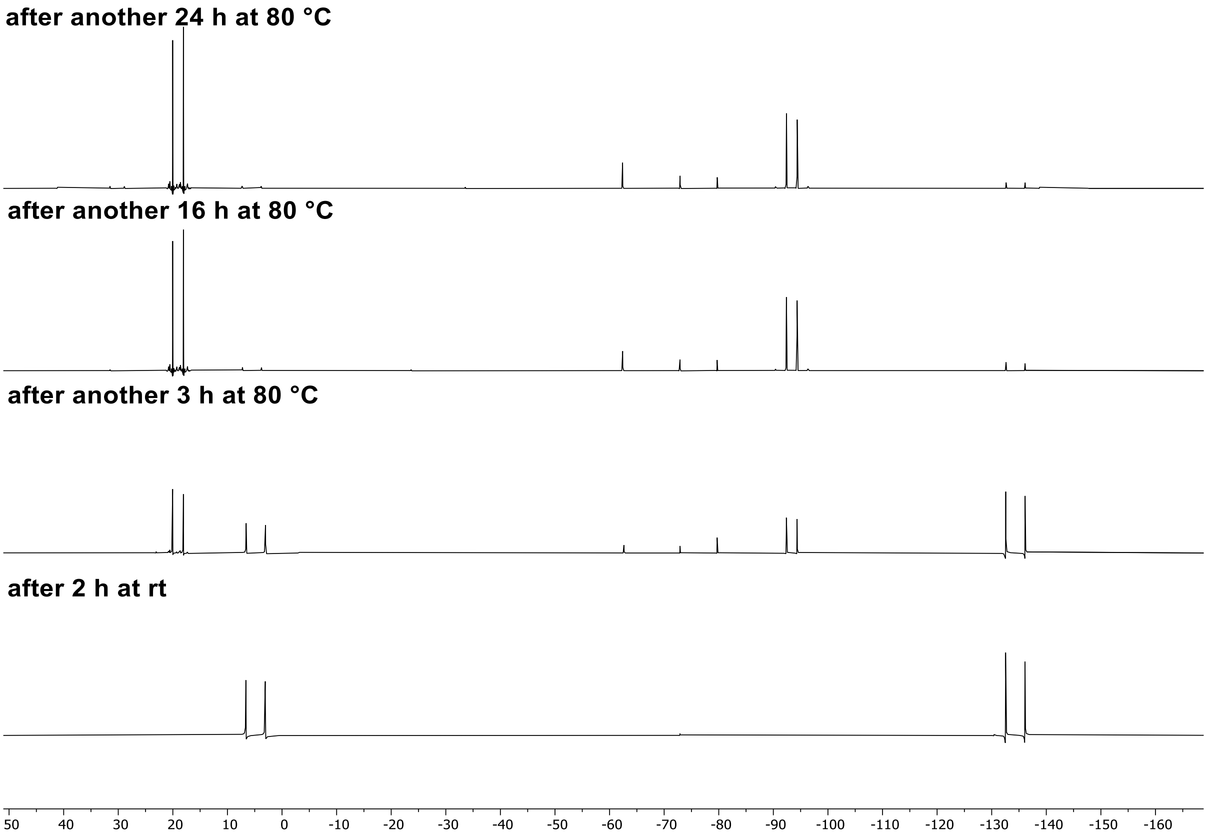

Figure S15. Monitoring of the reaction of ^{Dipp}TerSn(hmds) (**Sn1b**) with Mes*PP(CH_3)$_3$ (**P1a**) via $^{31}P\{^1H\}$ NMR spectroscopy (161 MHz, C_6D_6, 298 K).

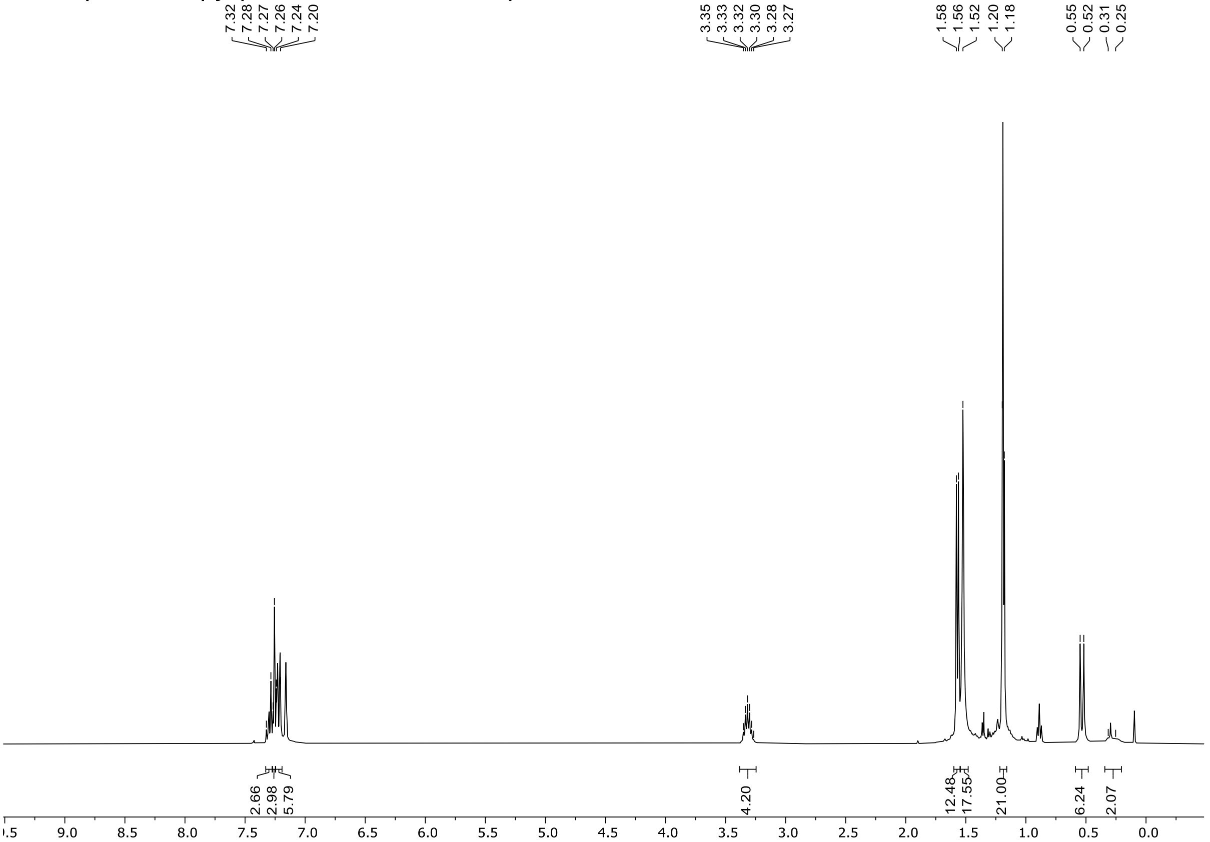

Figure S16. ^1H NMR spectrum of ^{Dipp}TerSnCH$_2$P(CH_3)$_2$=PMes* (**Sn2b**) (400 MHz, C_6D_6, 298 K); 0.89 and 1.24 ppm: *n*-hexane.

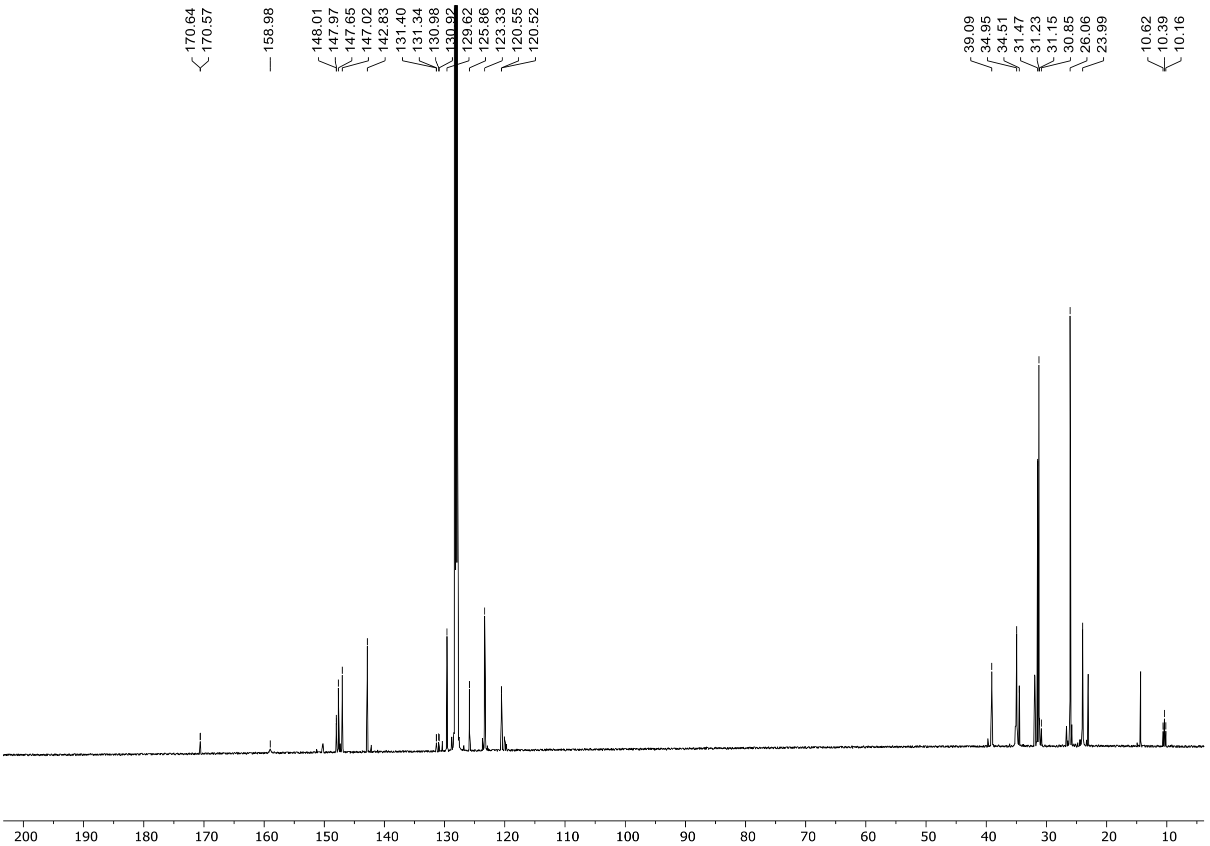

Figure S17. $^{13}C\{^1H\}$ NMR spectrum of $^{Dipp}TerSnCH_2P(CH_3)_2{=}PMes^*$ (**Sn2b**) (151 MHz, C_6D_6, 298 K); 14.3, 23.0, and 32.0 ppm: *n*-hexane.

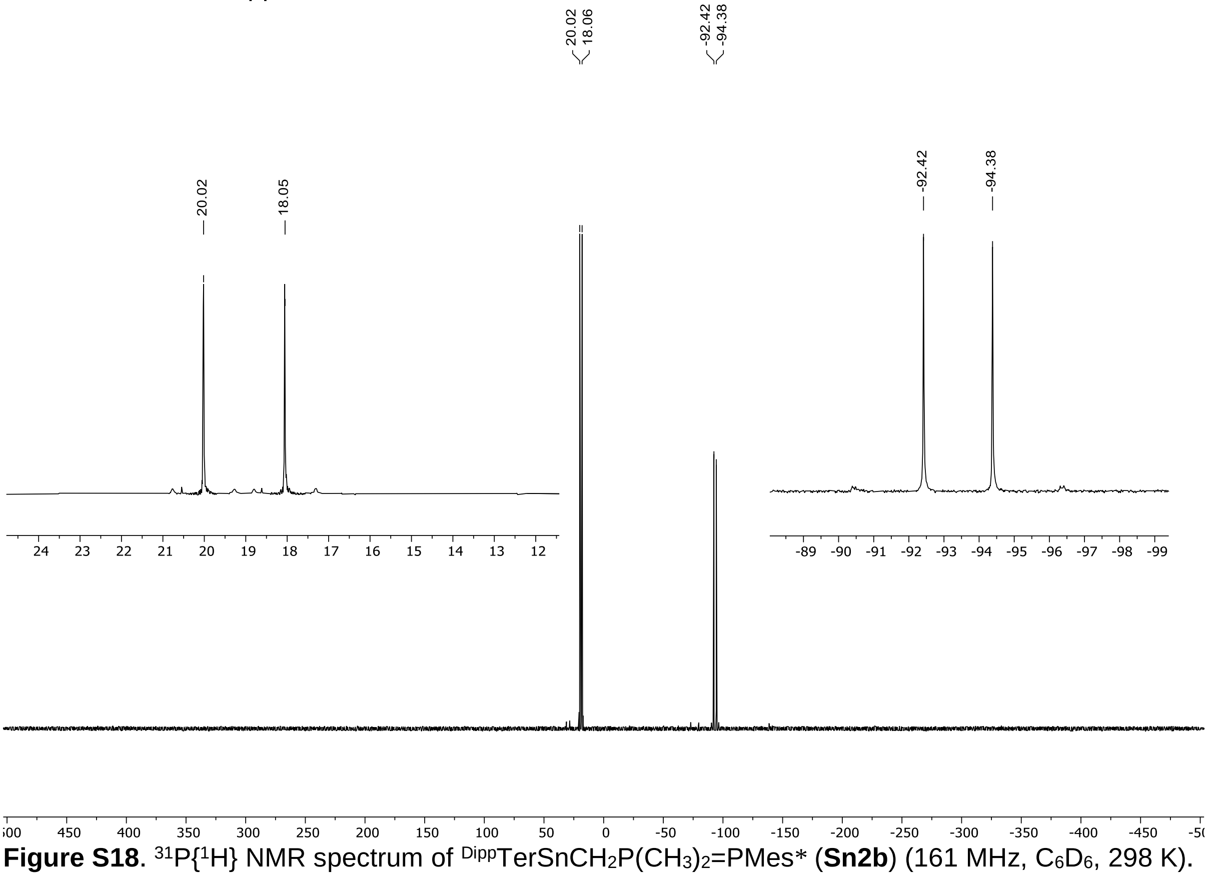

Figure S18. $^{31}P\{^1H\}$ NMR spectrum of $^{Dipp}TerSnCH_2P(CH_3)_2{=}PMes^*$ (**Sn2b**) (161 MHz, C_6D_6, 298 K).

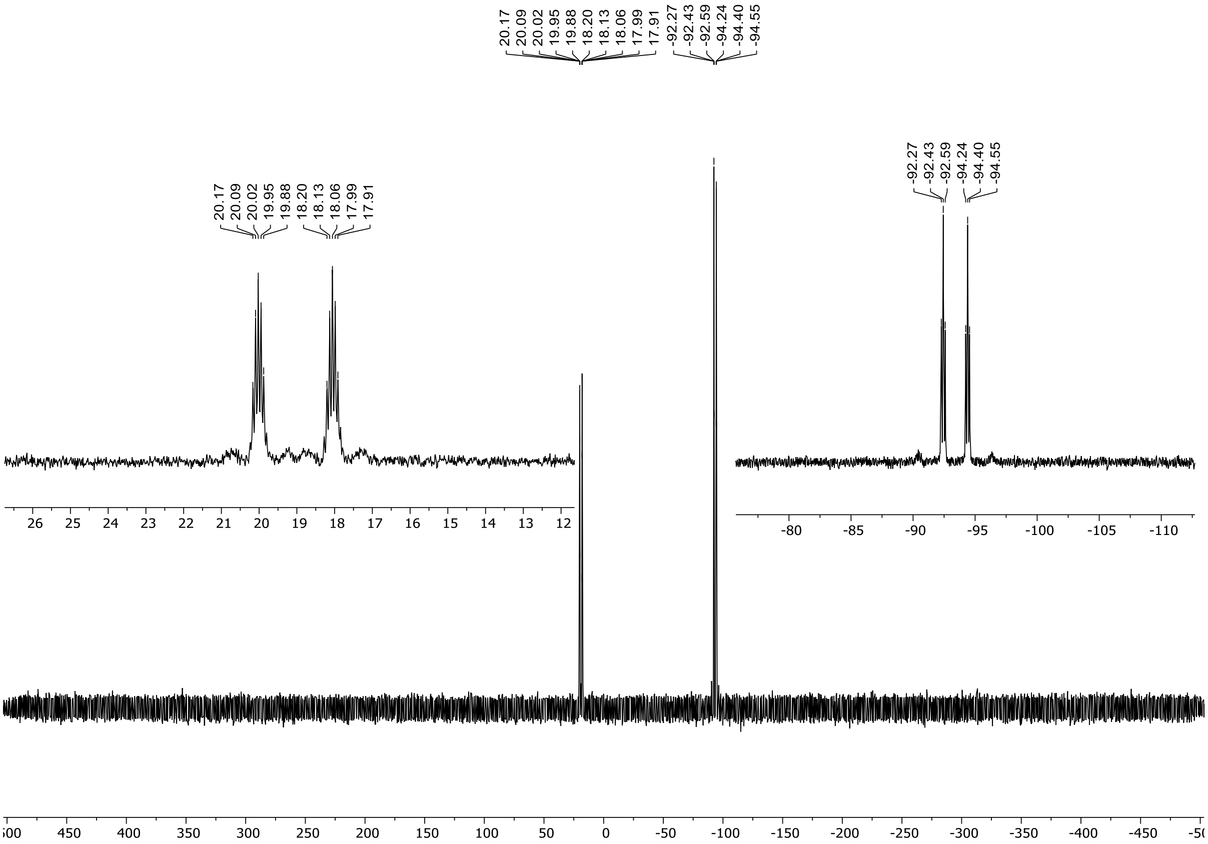

Figure S19. ^{31}P NMR spectrum of ^{Dipp}TerSnCH$_2$P(CH$_3$)$_2$=PMes* (**Sn2b**) (161 MHz, C_6D_6, 298 K).

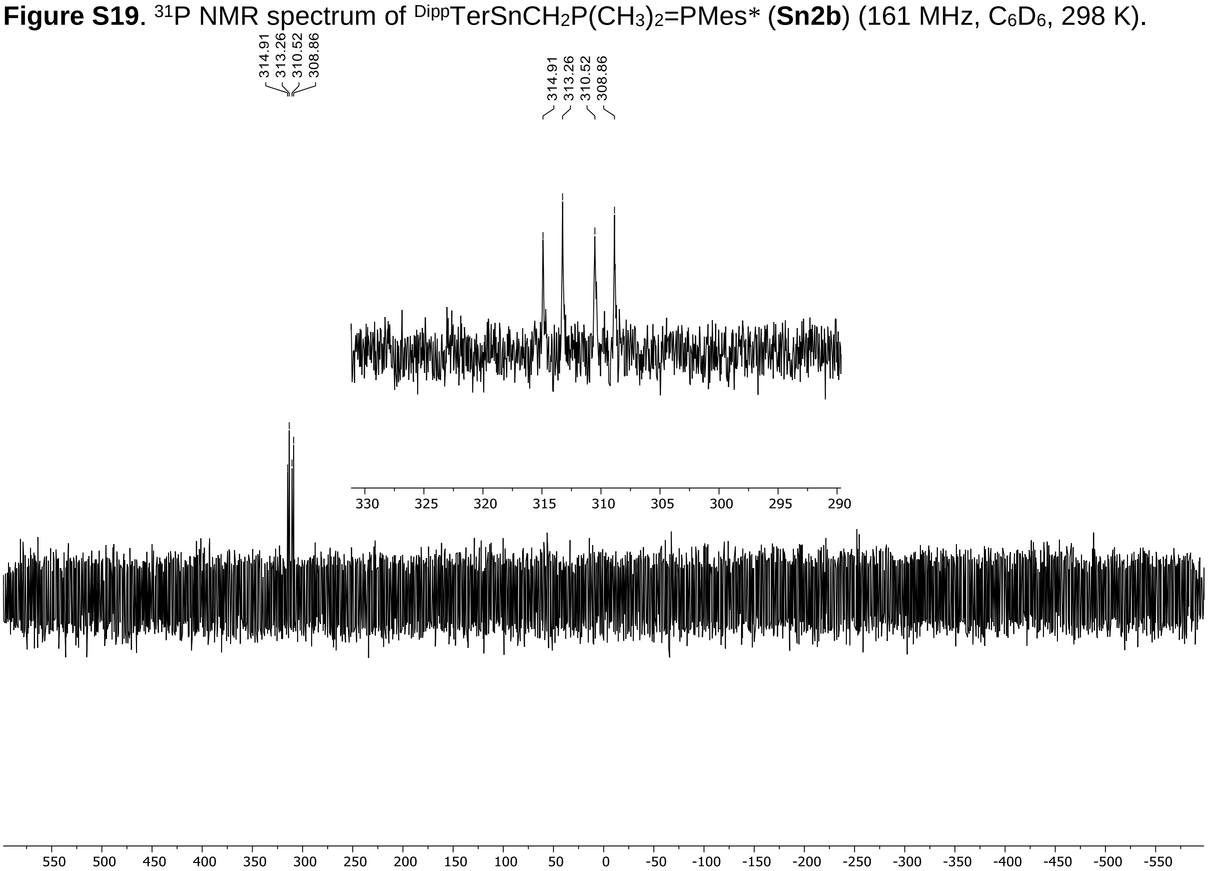

Figure S20. ^{119}Sn{^{1}H} NMR spectrum of ^{Dipp}TerSnCH$_2$P(CH$_3$)$_2$=PMes* (**Sn2b**) (149 MHz, C_6D_6, 298 K).

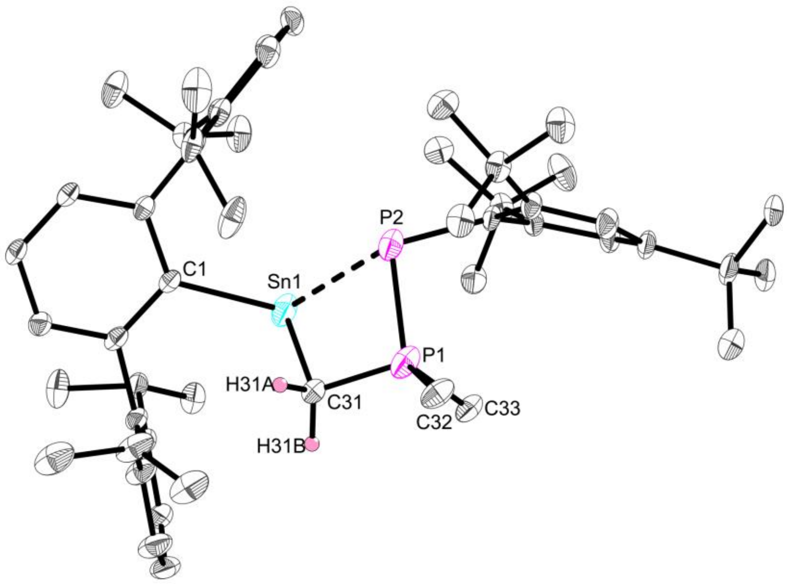

Figure S21. Molecular structure of $^{Dipp}TerSnCH_2P(CH_3)_2$=PMes* (**Sn2b**) in the crystal. Thermal ellipsoids are drawn at the 50% probability level (hydrogen atoms except H49A and H49B have been omitted for clarity). Selected bond lengths (Å) and angles (deg): Sn1•••P2 2.8345(8), Sn1–C1 2.258(3), Sn1–C31 2.270(4), P1–P2 2.1323(12), P1–C31 1.755(4), C1–Sn1–P2 107.13(8), C1–Sn1–C31 95.24(11).

Synthesis of ^{Dipp}TerSnCH$_2$P(CH$_3$)$_2$=P^{Mes}Ter (Sn2c)

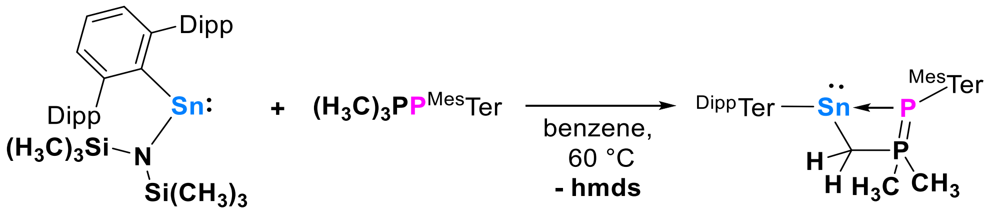

In an initial NMR scale experiment ^{Dipp}TerSn(hmds) (**Sn1b**) (0.054 g, 0.071 mmol) and ^{Mes}TerPP(CH$_3$)$_3$ (**P1b**) (0.030 g, 0.071 mmol) were dissolved in 0.5 mL of C_6D_6 and the reaction progress was monitored by ^{31}P{^{1}H} NMR spectroscopy (Figure S22). Directly after 10 minutes the formation of a new product can be observed. After additional 42 hours at room temperature the ratio of the respective ^{31}P{^{1}H} NMR signals of the starting material and the new compounds amounts to 1.0:0.6 (integration of the respective ^{31}P{^{1}H} NMR signals). Therefore, the reaction mixture was heated to 60 °C for 2 hours which resulted in clean formation of ^{Dipp}TerSnCH$_2$P(CH$_3$)$_2$=P^{Mes}Ter (**Sn2c**). Subsequent reactions were performed at 60 for 2 hours which also showed to lead to fully consumption of both starting materials and clean formation of **Sn2c**. All volatiles were removed under vacuum followed by addition of ca. 1 mL of *n*-pentane. The suspension was filtered and subsequently stored at 4 °C which results the formation of yellow crystals. These crystals were suitable for single crystal X-ray diffraction. The supernatant was removed and the remaining solid dried under vacuum to yield ^{Dipp}TerSnCH$_2$P(CH$_3$)$_2$=P^{Mes}Ter (**Sn2c**) as a yellow crystalline solid.

Yield: 0.042 g (0.045 mmol; 63%).
^{1}H NMR (400 MHz, C_6D_6, 298 K): δ = 0.30-0.40 (m(br), 2H, CH$_2$), 0.90-1.04 (m(br), 6H, P(CH$_3$)$_2$), 1.13 (d, $^3J_{H,H}$ = 6.6 Hz, 12H, CH(C*H*$_3$)$_2$), 1.31 (d, $^3J_{H,H}$ = 6.9 Hz, 12H, CH(C*H*$_3$)$_2$), 1.92 (s, 12H, CH$_3$), 2.34 (s, 6H, CH$_3$), 3.07 (hept, $^3J_{H,H}$ = 6.8 Hz, 4H, C*H*(CH$_3$)$_2$), 6.78-6.80 (m, 2H, CH$_{Aryl}$), 6.84-6.85 (m, 4H, CH$_{Aryl}$), 6.96-6.98 (m, 1H, CH$_{Aryl}$), 7.16-7.17 (m, 6H, CH$_{Aryl}$)*, 7.20-7.23 (m, 1H, CH$_{Aryl}$), 7.26-7.28 (m, 2H, CH$_{Aryl}$) ppm.
* = overlap with C_6D_5H signal
^{13}C{^{1}H} NMR (126 MHz, C_6D_6, 298 K): δ = 5.9 (dd, $^1J_{P,C}$ = 28.7 Hz, $^2J_{P,C}$ = 6.3 Hz, CH$_2$), 20.5 (br, P(CH$_3$)$_2$), 21.3 (CH$_3$), 22.4 (CH$_3$), 23.6 (CH($\underline{C}$H$_3$)$_2$), 26.9 (CH($\underline{C}$H$_3$)$_2$), 31.1 ($\underline{C}$H(CH$_3$)$_2$), 123.0 (CH$_{Aryl}$), 124.8 (CH$_{Aryl}$), 127.7 (CH$_{Aryl}$)**, 128.0 (CH$_{Aryl}$)**, 129.2 (CH$_{Aryl}$), 130.0 (CH$_{Aryl}$), 130.1 (CH$_{Aryl}$), 134.2 (dd, $J_{P,C}$ = 58.1 Hz, $J_{P,C}$ = 10.1 Hz, C$_{q,Aryl}$), 135.9 (br, C$_{q,Aryl}$), 136.1 (C$_{q,Aryl}$), 141.0 (C$_{q,Aryl}$), 141.8 (C$_{q,Aryl}$), 146.8 (C$_{q,Aryl}$), 147.5 (C$_{q,Aryl}$), 150.5 (br, C$_{q,Aryl}$), 171.3 (m, C$_{q,Aryl}$) ppm.
** = overlap with C_6D_6 signal and assigned by ^{1}H/^{13}C HSQC/HMBC
^{31}P{^{1}H} NMR (161 MHz, C_6D_6, 298 K): δ = -98.6 (d, $^1J_{P,P}$ = 312.1 Hz, Sn satellites: $J_{119Sn,P}$ = 579.1 Hz, $J_{117Sn,P}$ = 553.5 Hz, P^{Mes}Ter), 14.3 (d, $^1J_{P,P}$ = 312.1 Hz, $J_{119Sn,P}$ = 307.0 Hz, $J_{117Sn,P}$ = 293.9 Hz, H$_2$CP(CH$_3$)$_2$) ppm.
^{31}P NMR (161 MHz, C_6D_6, 298 K): δ = -98.6 (d, $^1J_{P,P}$ = 311.7 Hz, P^{Mes}Ter), 14.3 (dhept, $^1J_{P,P}$ = 312.0 Hz, $J_{P,H}$ = 11.5 Hz, H$_2$CP(CH$_3$)$_2$)
^{119}Sn{^{1}H} NMR (149 MHz, C_6D_6, 298 K): δ = 145.9 (dd, $J_{119Sn,P}$ = 580.7 Hz, $J_{119Sn,P}$ = 307.3 Hz) ppm.
EA: Anal. calcd. for $C_{57}H_{70}P_2Sn$: C, 73.16; H, 7.54; Found: C, 73.29; H, 7.76.

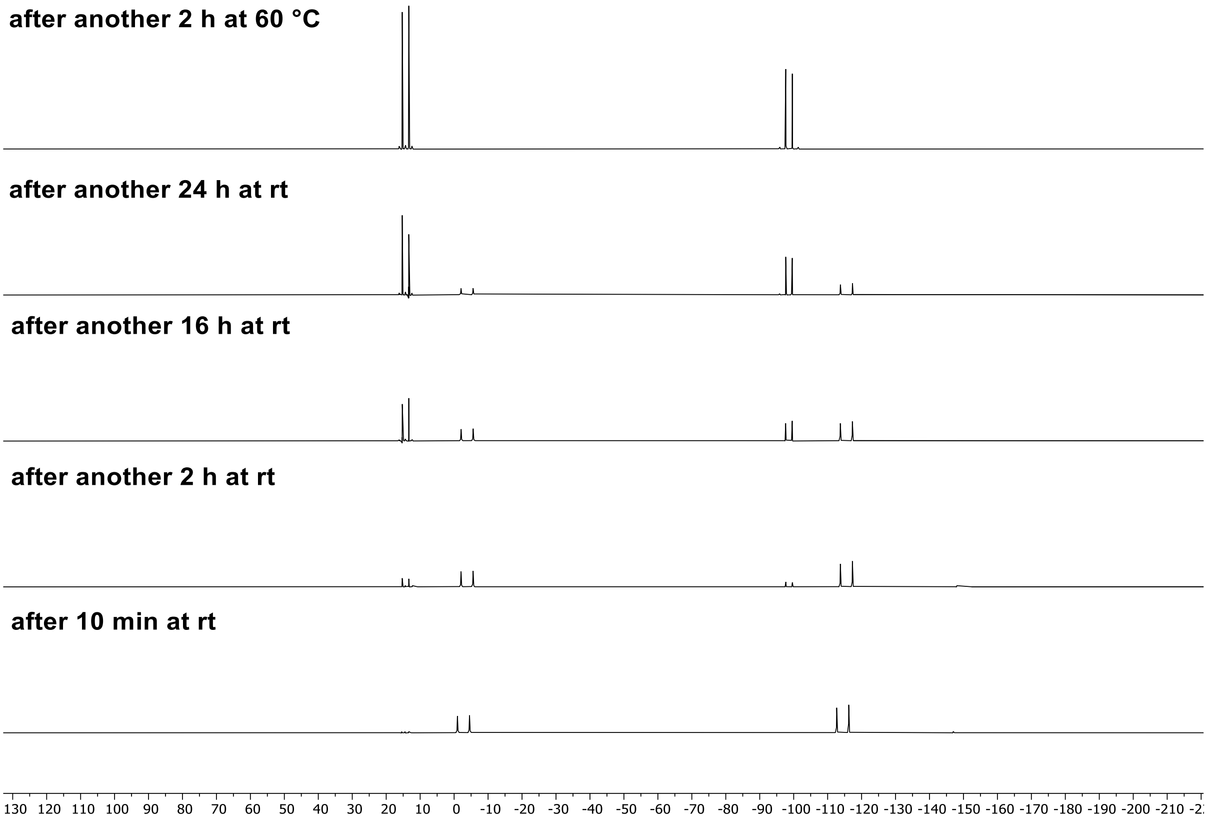

Figure S22. Monitoring of the reaction of ^{Dipp}TerSn(hmds) (**Sn1b**) with ^{Mes}TerPP(CH$_3$)$_3$ (**P1b**) via ^{31}P{^{1}H} NMR spectroscopy (161 MHz, C$_6$D$_6$, 298 K).

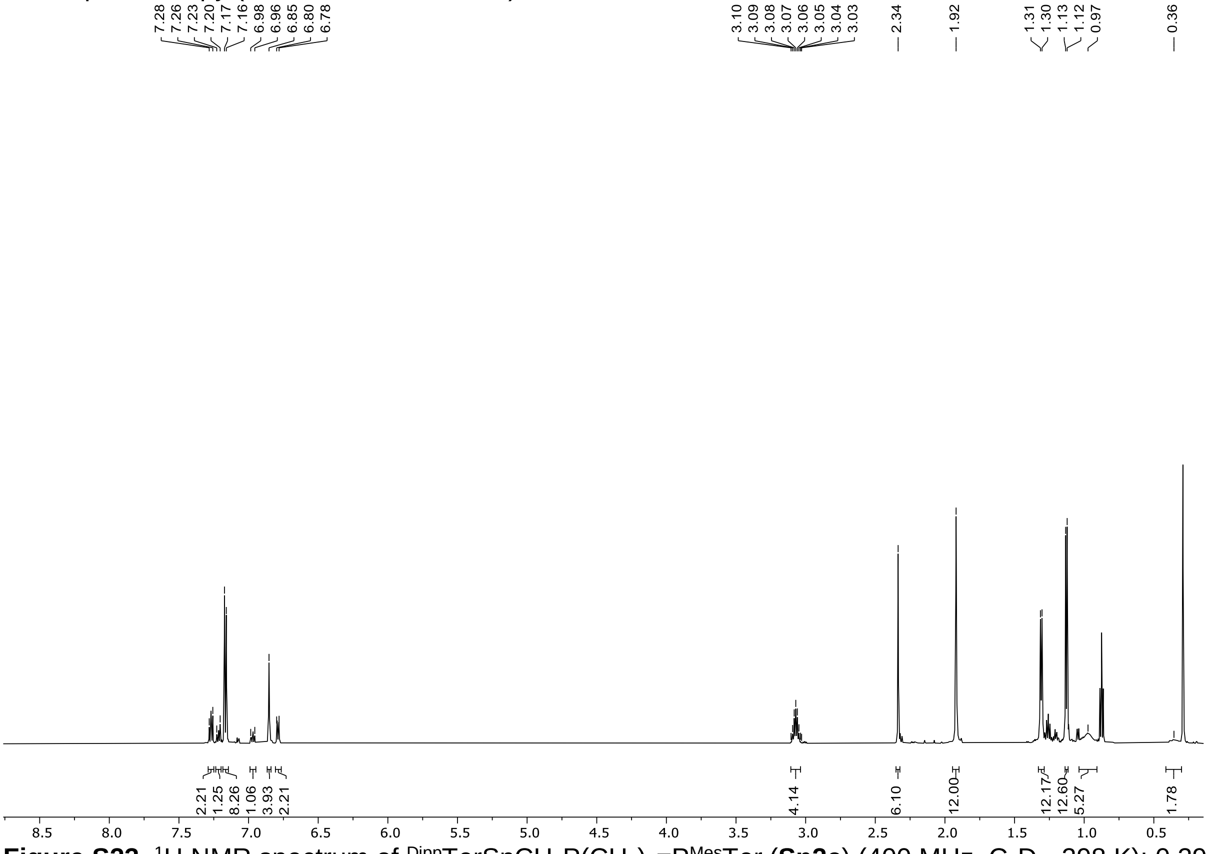

Figure S23. ^{1}H NMR spectrum of ^{Dipp}TerSnCH$_2$P(CH$_3$)$_2$=P^{Mes}Ter (**Sn2c**) (400 MHz, C$_6$D$_6$, 298 K); 0.29 ppm: silicon grease, 0.87 and 1.23 ppm: *n*-pentane.

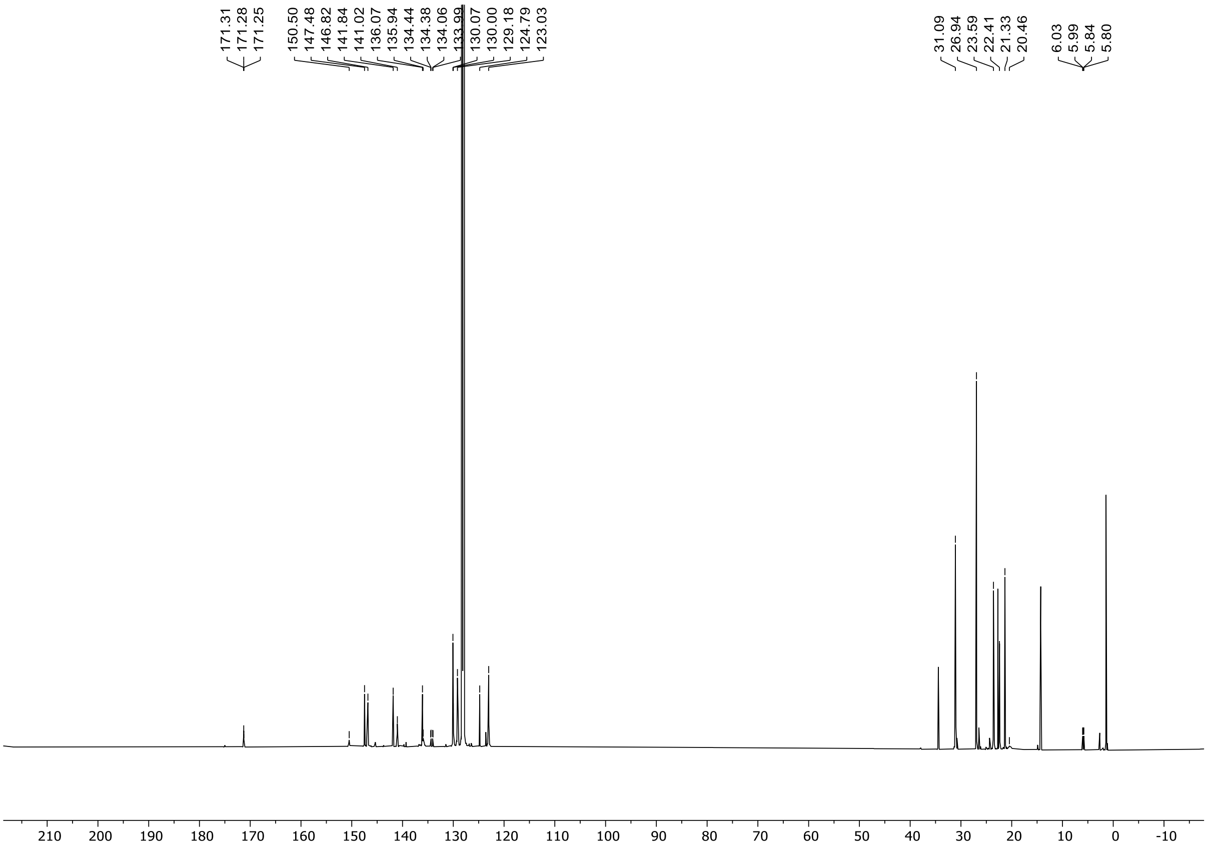

Figure S24. $^{13}C\{^{1}H\}$ NMR spectrum of ^{Dipp}TerSnCH$_2$P(CH$_3$)$_2$=P^{Mes}Ter (**Sn2c**) (151 MHz, C_6D_6, 298 K); 1.4 ppm: silicon grease, 14.4, 23.1, and 34.9 ppm: *n*-pentane.

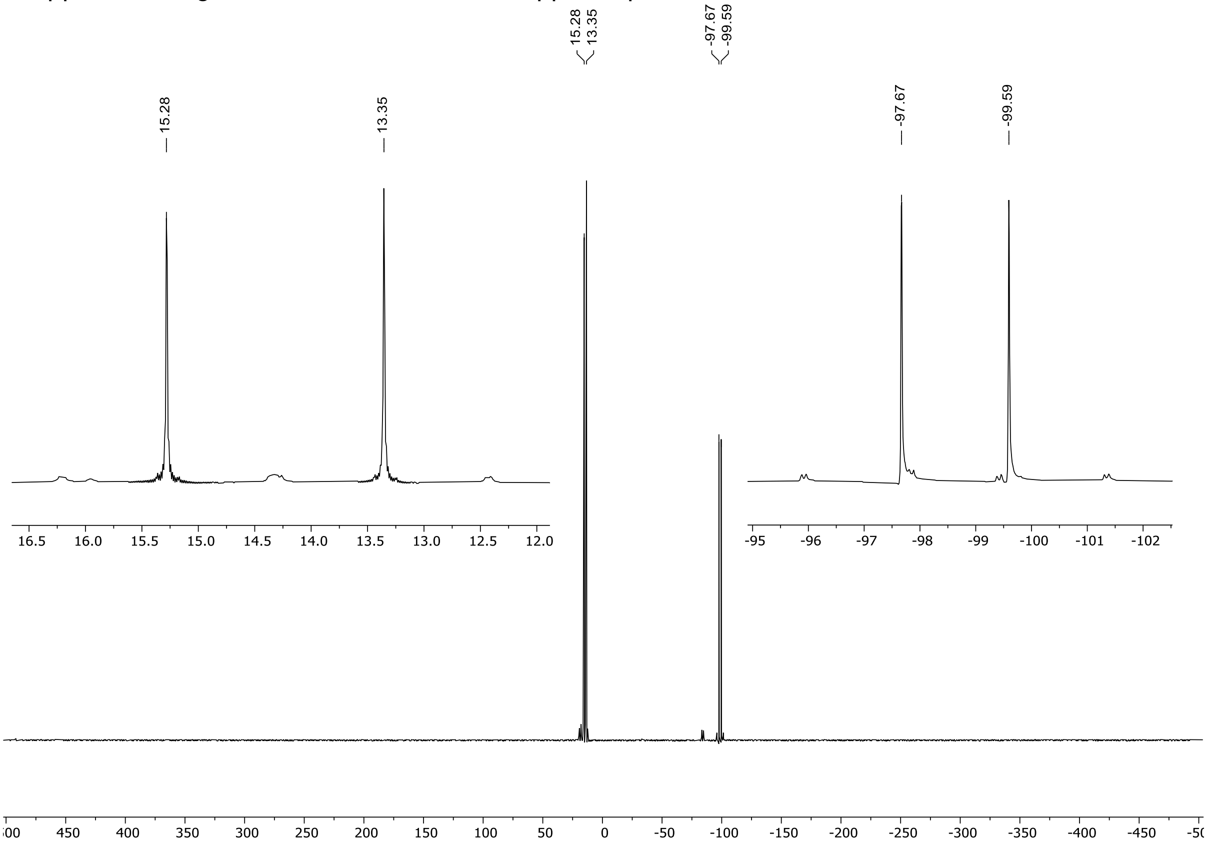

Figure S25. $^{31}P\{^{1}H\}$ NMR spectrum of ^{Dipp}TerSnCH$_2$P(CH$_3$)$_2$=P^{Mes}Ter (**Sn2c**) (161 MHz, C_6D_6, 298 K).

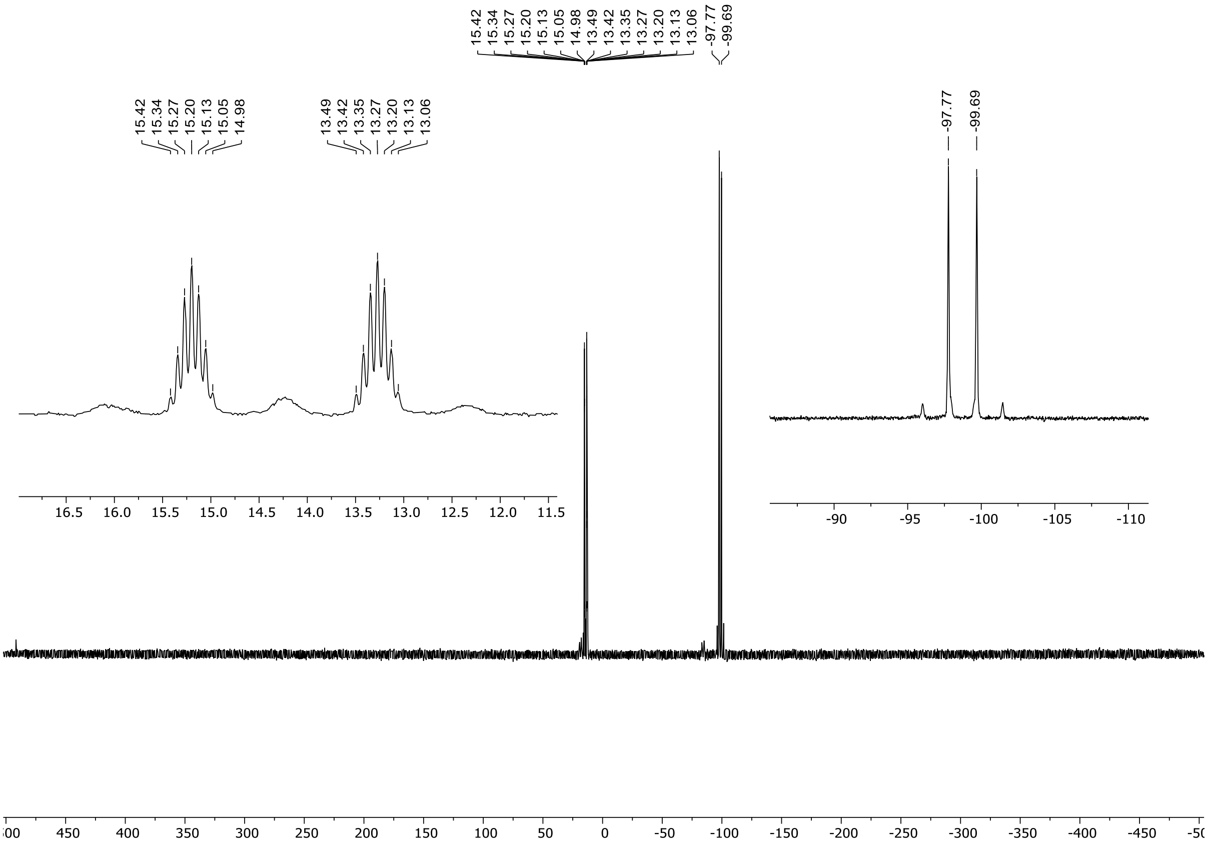

Figure S26. ^{31}P NMR spectrum of $^{Dipp}TerSnCH_2P(CH_3)_2{=}P^{Mes}Ter$ (**Sn2c**) (161 MHz, C_6D_6, 298 K).

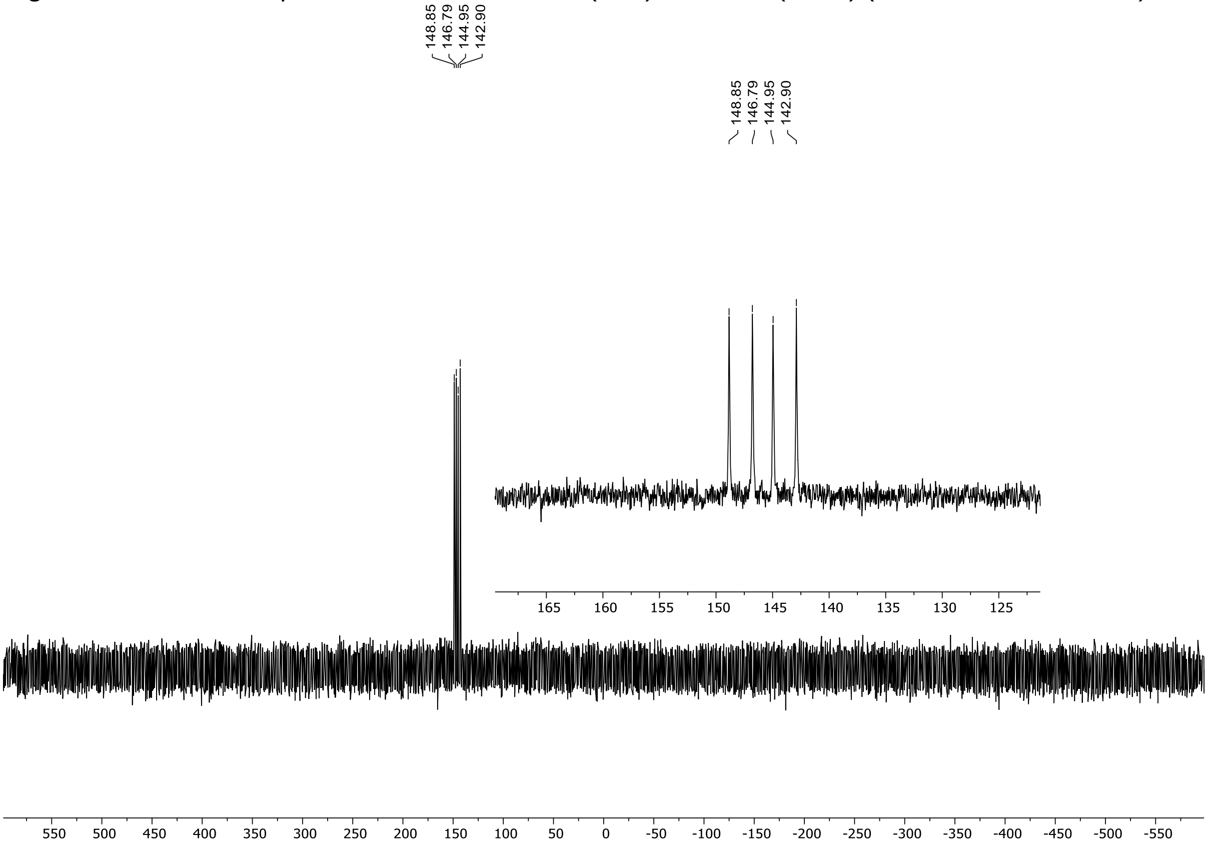

Figure S27. $^{119}Sn\{^1H\}$ NMR spectrum of $^{Dipp}TerSnCH_2P(CH_3)_2{=}P^{Mes}Ter$ (**Sn2c**) (149 MHz, C_6D_6, 298 K).

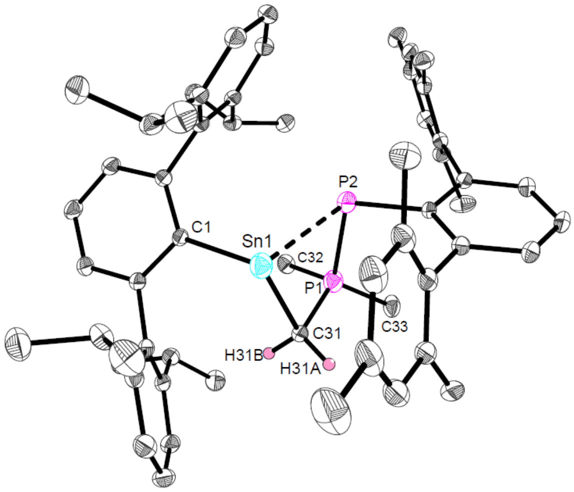

Figure S28. Molecular structure of $^{Dipp}TerSnCH_2P(CH_3)_2{=}P^{Mes}Ter$ (**Sn2c**) in the crystal. Thermal ellipsoids are drawn at the 50% probability level (hydrogen atoms except H31A and H31B have been omitted for clarity). Selected bond lengths (Å) and angles (deg): Sn1•••P2 2.7727(7), Sn1–C1 2.275(3), Sn1–C31 2.307(3), P1–P2 2.1495(10), P1–C34 1.759(3), C1–Sn1–P2 98.23(7), C1–Sn1–C31 97.90(10).

Synthesis of ^{Dipp}TerSn($CH_2P(CH_3)_2$)=P^{Dipp}Ter (Sn2d) / ^{Dipp}TerSn[$CH_2P(CH_3)_2$]=P^{Dipp}Ter (Sn3) and side product formation of [^{Dipp}TerPP$(CH_3)_2$CHSn]$_2$ (Sn4)

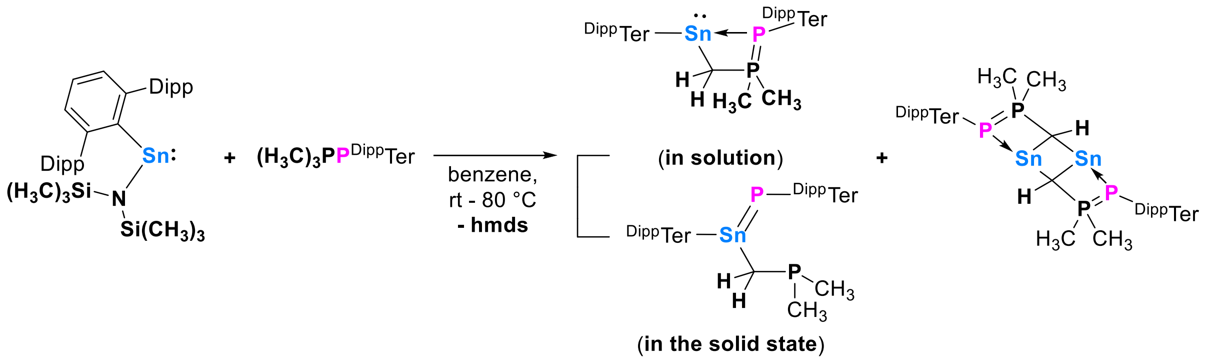

In an initial experiment ^{Dipp}TerSn(hmds) (**Sn1b**) (0.070 g, 0.103 mmol) and ^{Dipp}TerPP$(CH_3)_3$ (**P1c**) (0.052 g, 0.103 mmol) were dissolved in 0.6 mL of C_6D_6 and the reaction progress was monitored by ^{31}P{^{1}H} NMR spectroscopy (Figure S29). Even at room temperature a new set of doublet signals was observed which could later be assigned to [^{Dipp}TerPP$(CH_3)_2$CHSn]$_2$ (**Sn4**). After additional time at room temperature another set of doublet signals is observable. The reaction mixture was heated to 80 °C until one of the starting materials was completely consumed. Removal of all volatile components under vacuum followed by washing of the orange crude product with *n*-hexane yielded ^{Dipp}TerSn($CH_2P(CH_3)_2$)=P^{Dipp}Ter (**Sn2d**) / ^{Dipp}TerSn($CH_2P(CH_3)_2$)=P^{Dipp}Ter (**Sn3**) as an orange solid. For all further experiments the reaction mixture was heated to to 80 °C until one of the starting materials was completely consumed followed by the above-mentioned work-up procedure.
Crystals of **Sn3** suitable for single crystal X-ray diffraction were obtained from a saturated solution of **Sn3** in *n*-hexane at -30 °C.
Small amounts of yellow crystals of [^{Dipp}TerPP$(CH_3)_2$CHSn]$_2$ (**Sn4**) were obtained from a concentrated benzene solution of the reaction mixture at room temperature.

Yield: 0.034 g (0.039 mmol; 59%).
^{1}H NMR (400 MHz, C_6D_6, 298 K): δ = 0.10 (dd, $J_{P,H}$ = 18.0 Hz, $^2J_{P,H}$ = 10.4 Hz, 2H, CH_2), 0.86 (d, $^2J_{P,H}$ = 12.3 Hz, 6H, P$(CH_3)_2$), 1.01 (d, $^3J_{H,H}$ = 6.7 Hz, 12H, CH(C*H*$_3$)$_2$), 1.10 (d, $^3J_{H,H}$ = 6.8 Hz, 12H, CH(C*H*$_3$)$_2$), 1.13 (d, $^3J_{H,H}$ = 6.7 Hz, 12H, CH(C*H*$_3$)$_2$), 1.20 (d, $^3J_{H,H}$ = 6.9 Hz, 12H, CH(C*H*$_3$)$_2$), 2.70 (hept, $^3J_{H,H}$ = 6.8 Hz, 4H, C*H*$(CH_3)_2$), 3.11 (hept, $^3J_{H,H}$ = 6.8 Hz, 4H, C*H*$(CH_3)_2$), 6.87-6.90 (m, 1H, CH_{Aryl}), 6.98-6.99 (m, 2H, CH_{Aryl}), 7.07-7.08 (m, 2H, CH_{Aryl}), 7.11-7.14 (m, 1H, CH_{Aryl}), 7.15-7.17 (m, 8H, CH_{Aryl})*, 7.26-7.32 (m, 4H, CH_{Aryl}) ppm.
* = overlap with C_6D_5H signal
^{13}C{^{1}H} NMR (126 MHz, C_6D_6, 298 K): δ = 9.8 (dd, $J_{P,C}$ = 34.9 Hz, $J_{P,C}$ = 28.3 Hz, CH_2), 19.9 (s(br), P$(CH_3)_2$), 23.7 (CH(<u>C</u>H$_3$)$_2$), 23.8 (CH(<u>C</u>H$_3$)$_2$), 26.1 (CH(<u>C</u>H$_3$)$_2$), 26.5 (CH(<u>C</u>H$_3$)$_2$), 30.9 (<u>C</u>H$(CH_3)_2$), 31.0 (<u>C</u>H$(CH_3)_2$), 123.2 (CH_{Aryl}), 123.7 (CH_{Aryl}), 125.5 (CH_{Aryl}), 125.9 (CH_{Aryl}), 127.9 (CH_{Aryl})**, 128.0 (CH_{Aryl})**, 130.4 (CH_{Aryl}), 131.9 (CH_{Aryl}), 136.9 (dd, $J_{P,C}$ = 61.1 Hz, $J_{P,C}$ = 10.3 Hz, $C_{q,Aryl}$), 142.2 (d, $J_{P,C}$ = 3.7 Hz, $C_{q,Aryl}$), 142.5 ($C_{q,Aryl}$), 146.8 ($C_{q,Aryl}$), 147.1 ($C_{q,Aryl}$), 147.7 ($C_{q,Aryl}$), 148.5 (dd, $J_{P,C}$ = 14.9 Hz, $J_{P,C}$ = 7.7 Hz, $C_{q,Aryl}$), 171.3 (d, $J_{P,C}$ = 10.2 Hz, $C_{q,Aryl}$) ppm.
** = overlap with C_6D_6 signal and assigned by ^{1}H/^{13}C HSQC/HMBC
^{31}P{^{1}H} NMR (161 MHz, C_6D_6, 298 K): δ = -95.2 (d, $^1J_{P,P}$ = 343.2 Hz, Sn satellites: $J_{119Sn,P}$ = 539.5 Hz, $J_{117Sn,P}$ = 514.9 Hz, Sn=P), 12.3 (d, $^1J_{P,P}$ = 343.2 Hz, $J_{119Sn,P}$ = 286.4 Hz, $J_{117Sn,P}$ = 274.4 Hz, $H_2CP(CH_3)_2$) ppm.
^{31}P NMR (161 MHz, C_6D_6, 298 K): δ = -95.2 (dt, $^1J_{P,P}$ = 343.3 Hz, $J_{P,H}$ = 18.1 Hz, Sn=P), 12.4 (dhept, $^1J_{P,P}$ = 343.2 Hz, $J_{P,H}$ = 11.9 Hz, $H_2CP(CH_3)_2$) ppm.
^{119}Sn{^{1}H} NMR (149 MHz, C_6D_6, 298 K): δ = (not observed) ppm.
EA: Anal. calcd. for $C_{63}H_{82}P_2Sn$: C, 74.19; H, 8.10; Found: C, 73.16; H, 7.82.

Characteristic $^{31}P\{^1H\}$ NMR signals of [^{Dipp}TerPP(CH_3)$_2$CHSn]$_2$ (**Sn4**):
$^{31}P\{^1H\}$ NMR (161 MHz, C_6D_6, 298 K): δ = -75.3 (d, $^1J_{P,P}$ = 346.2 Hz), 14.4 (d, $^1J_{P,P}$ = 345.7 Hz) ppm.

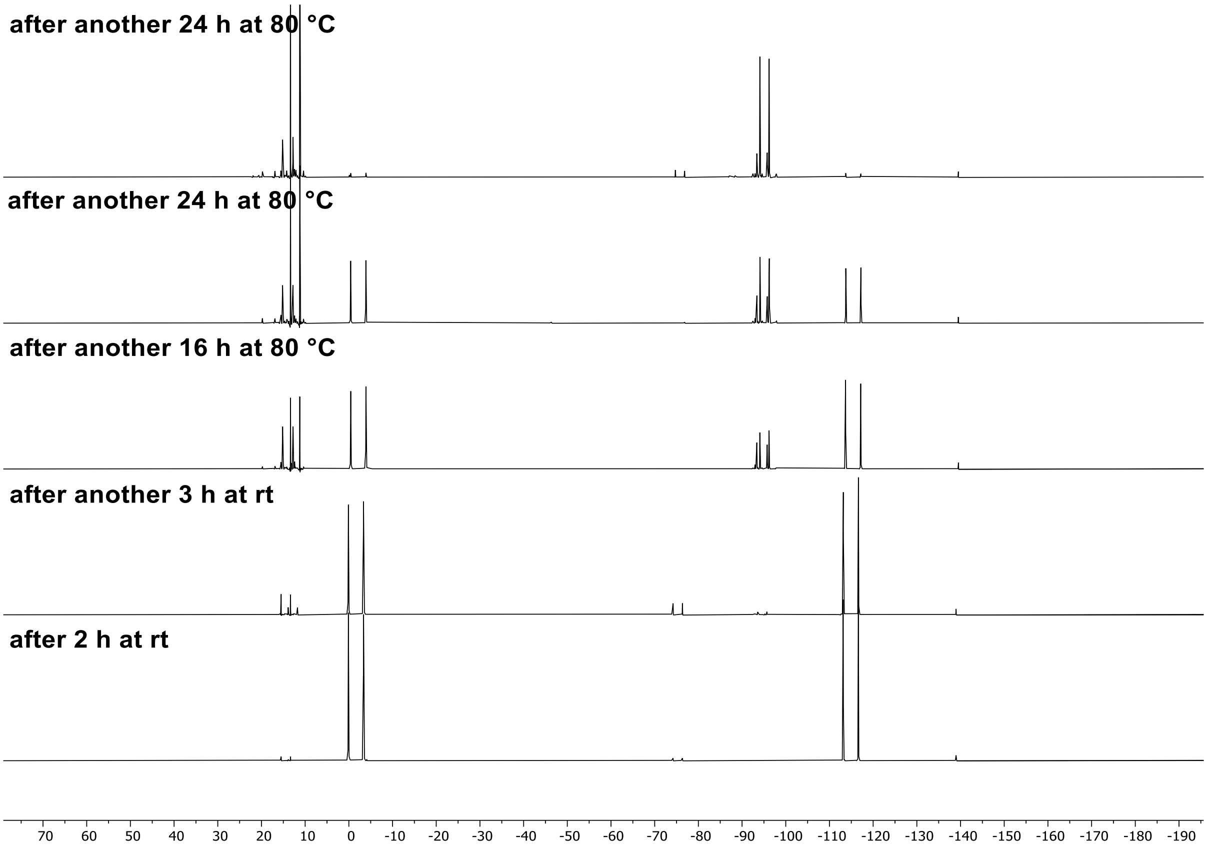

Figure S29. Monitoring of the reaction of ^{Dipp}TerSn(hmds) (**Sn1b**) with ^{Dipp}TerPP(CH_3)$_3$ (**P1c**) via $^{31}P\{^1H\}$ NMR spectroscopy (161 MHz, C_6D_6, 298 K).

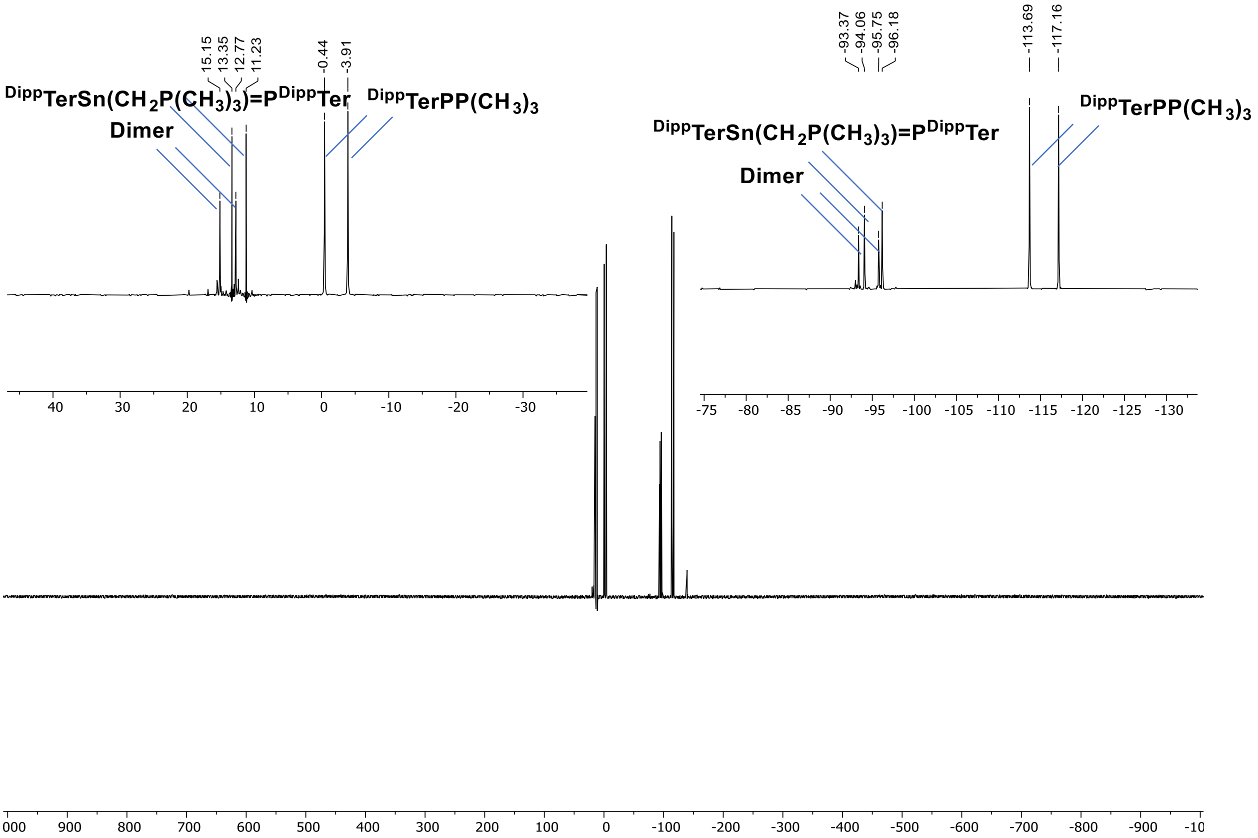

Figure S30. $^{31}P\{^1H\}$ NMR of the reaction of $^{Dipp}TerSn(hmds)$ (**Sn1b**) with $^{Dipp}TerPP(CH_3)_3$ (**P1c**) after 5 h at room temperature and 19 h at 80 °C (161 MHz, C_6D_6, 298 K).

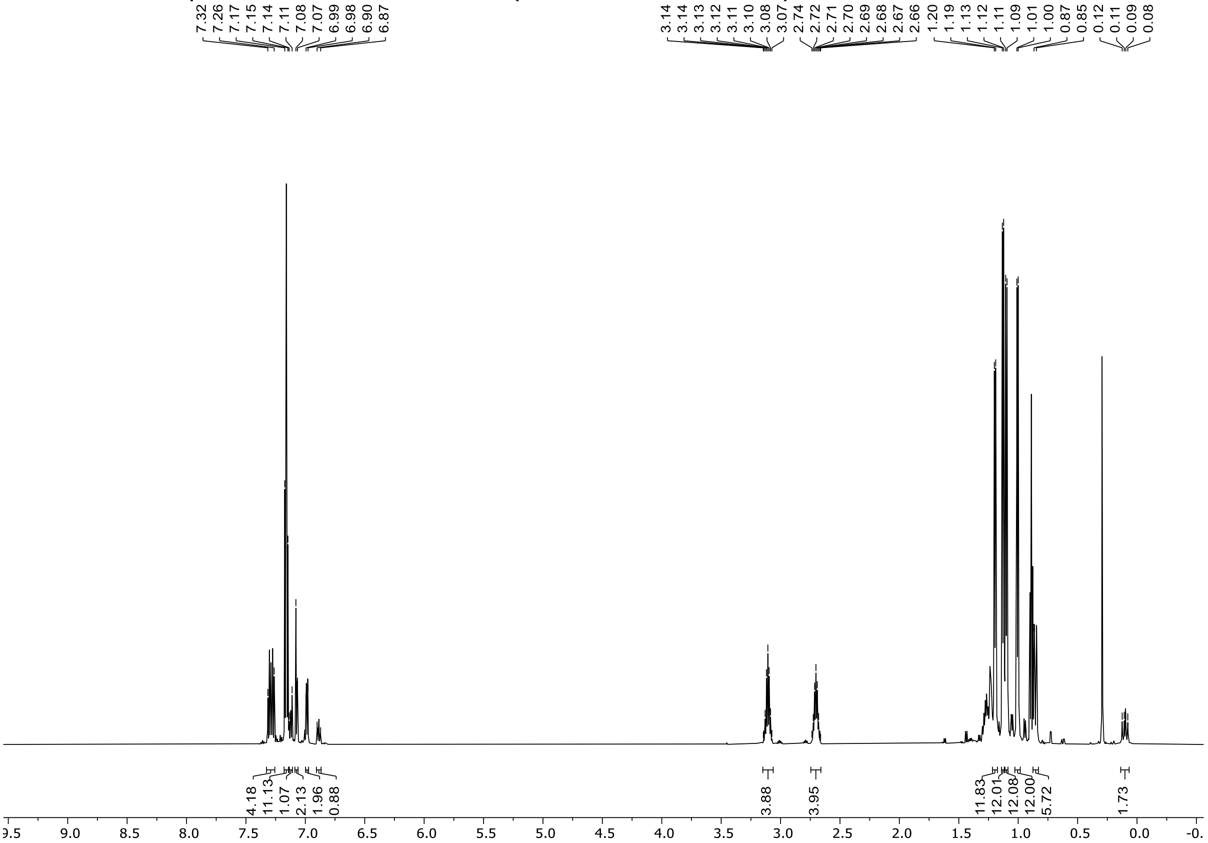

Figure S31. ^1H NMR spectrum of $^{Dipp}TerSn(CH_2P(CH_3)_2)=P^{Dipp}Ter$ (**Sn2d**) / $^{Dipp}TerSn(CH_2P(CH_3)_2)=P^{Dipp}Ter$ (**Sn3**) (400 MHz, C_6D_6, 298 K); 0.89 and 1.24 ppm: *n*-hexane.

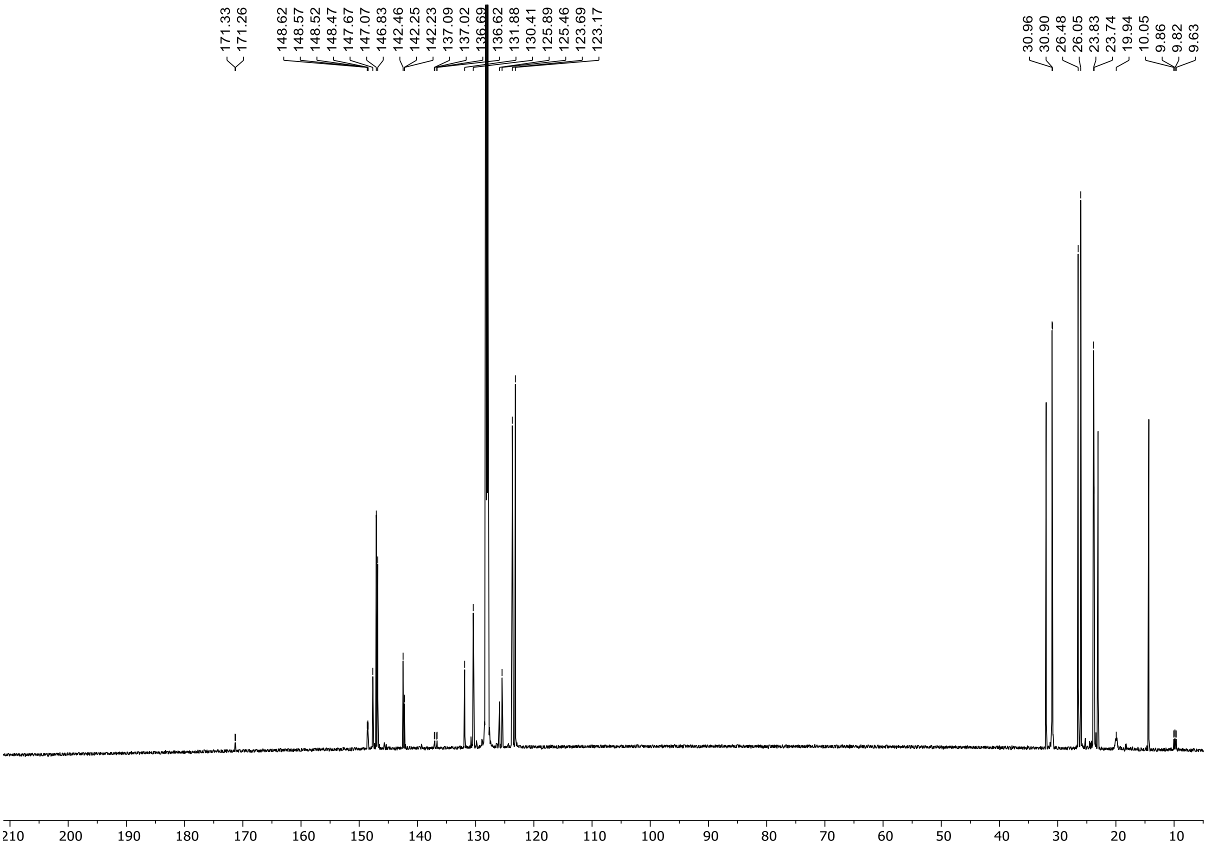

Figure S32. $^{13}C\{^{1}H\}$ NMR spectrum of ^{Dipp}TerSn($CH_2P(CH_3)_2$)=P^{Dipp}Ter (**Sn2d**) / ^{Dipp}TerSn($CH_2P(CH_3)_2$)=P^{Dipp}Ter (**Sn3**) (151 MHz, C_6D_6, 298 K); 14.3, 23.0, and 32.0 ppm: *n*-hexane.

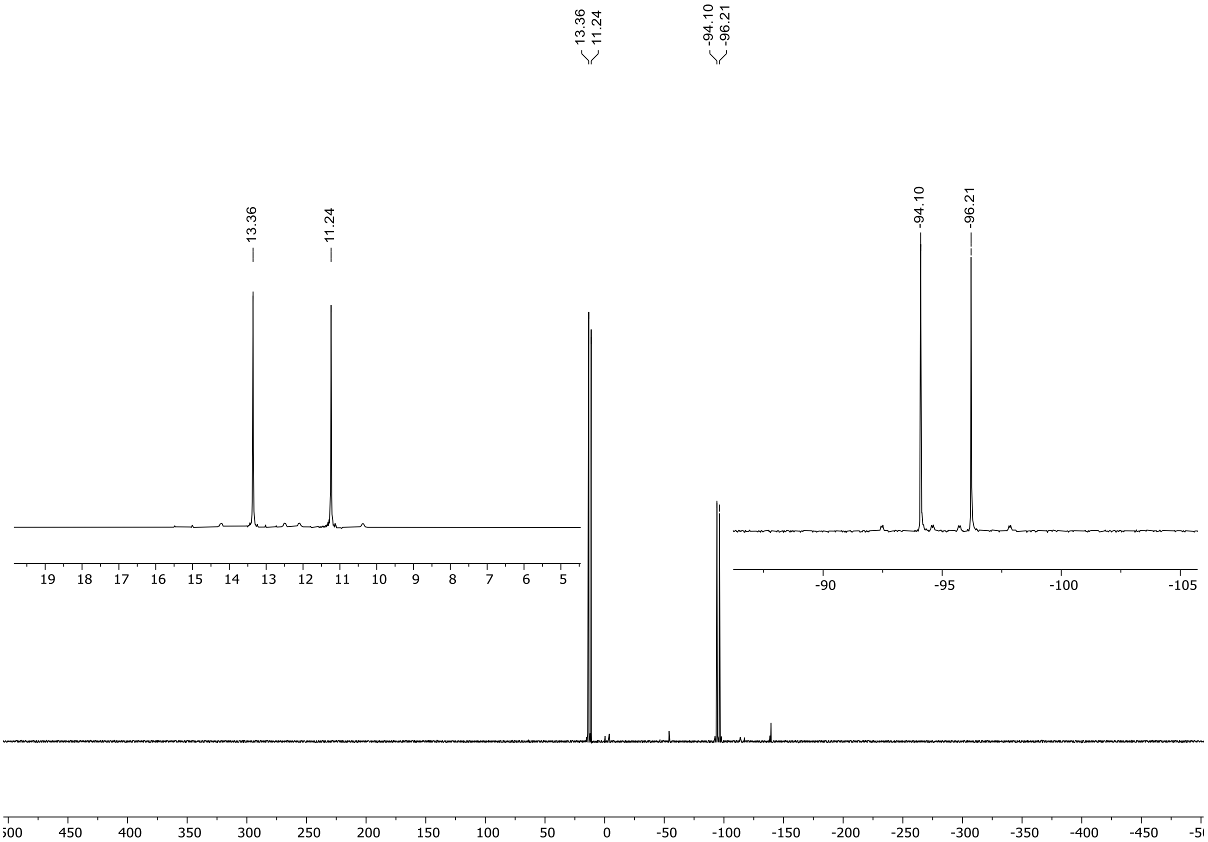

Figure S33. $^{31}P\{^{1}H\}$ NMR spectrum of ^{Dipp}TerSn($CH_2P(CH_3)_2$)=P^{Dipp}Ter (**Sn2d**) / ^{Dipp}TerSn($CH_2P(CH_3)_2$)=P^{Dipp}Ter (**Sn3**) (161 MHz, C_6D_6, 298 K).

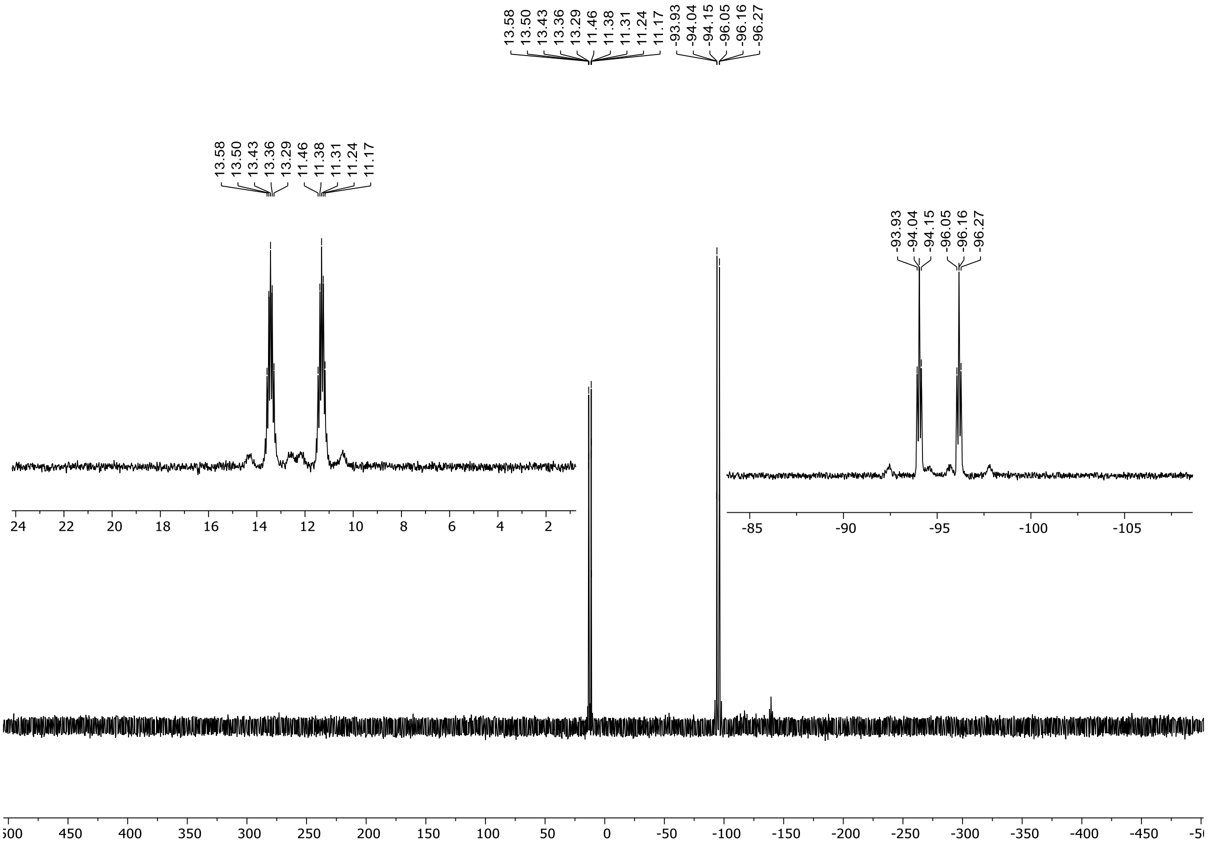

Figure S34. ^{31}P NMR spectrum of ^{Dipp}TerSn(CH$_2$P(CH$_3$)$_2$)=P^{Dipp}Ter (**Sn2d**) / ^{Dipp}TerSn(CH$_2$P(CH$_3$)$_2$)=P^{Dipp}Ter (**Sn3**) (161 MHz, C$_6$D$_6$, 298 K).

Table S1. Selected ^{1}H, ^{13}C, ^{31}P and ^{119}Sn NMR data of **Sn2a-d**.^{a}

	**Sn2a**	**Sn2b**	**Sn2c**	**Sn2d**
δ^1H / δ^{13}C{^{1}H} CH$_2$	0.78-0.92 (m(br) / 6.8 (dd)	0.25-0.31 (m(br)) / 10.2-10.6 (m)	0.30-0.40 (m(br)) / 5.9 (dd)	0.10 (dd) / 9.8 (dd)
δ^{13}C{^{1}H} C$_{q,Aryl}$Sn	169.0	170.6	171.3	171.3
^{31}P{^{1}H}	-91.4 and 19.1	-93.4 and 19.0	-98.6 and 14.3	-95.2 and 12.3
$^1J_{P,P}$	338.3	318.7	312.1	343.2
^{119}Sn{^{1}H}	128.9	311.9	145.9	^{b}
$J_{119Sn,P}$	617.8 and 231.3	656.8 and 249.7	580.7 and 307.3	539.5 and 286.4

^{a} Values are given in ppm and Hz. Measurements were carried out in C$_6$D$_6$ at room temperature.
^{b} Not observed

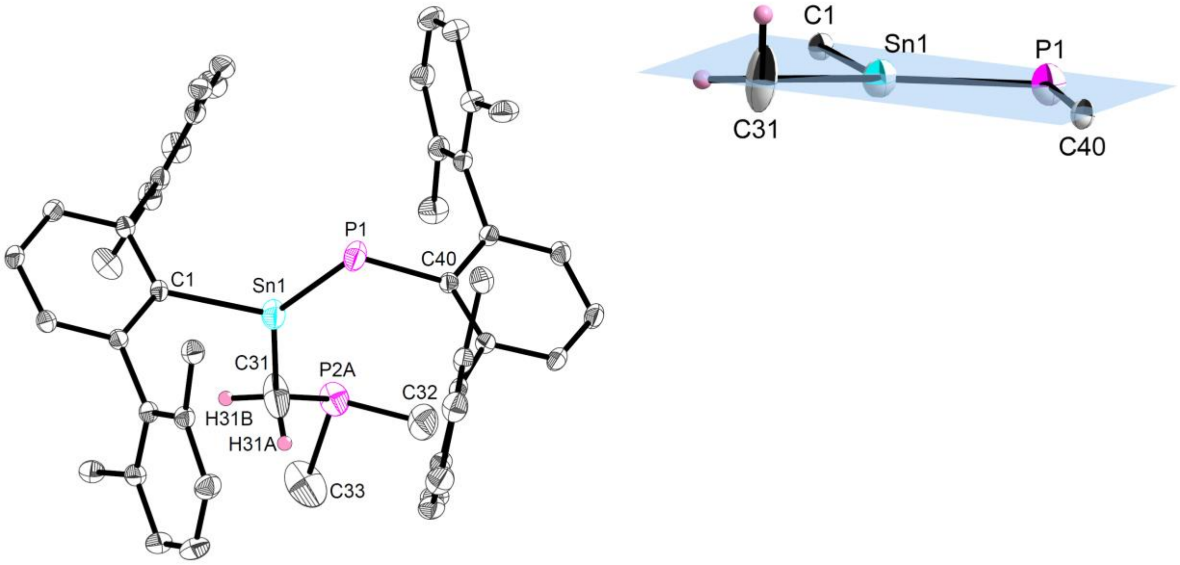

Figure S35. Left: Molecular structure of $^{Dipp}TerSn(CH_2P(CH_3)_2)=P^{Dipp}Ter$ (**Sn3**) in the crystal. Thermal ellipsoids are drawn at the 50% probability level (hydrogen atoms except H31A and H31B have been omitted for clarity). Selected bond lengths (Å) and angles (deg): Sn1–P1 2.3425(4), Sn1–C1 2.1677(16), Sn1–C31 2.145(2), P2A–C31 1.771(3), C1–Sn1–P1 113.75(4), C1–Sn1–C31 107.45(8), C31–Sn1–P1 138.33(8); Right: Excerpt of the molecular structure of **Sn3** in the crystal showing the planarity of the central unit of the stannaphosphene **Sn3**.

Note: The B alert arises from small average C-C distances in the co-crystallized disordered solvent molecule (C70T – C75T; 1.36 Å). Two sites have been refined.

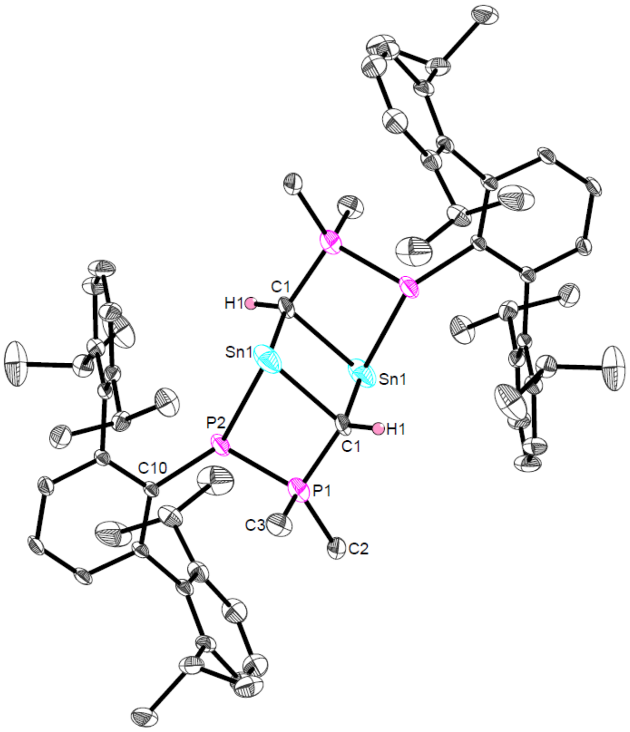

Figure S36. Molecular structure of $[^{Dipp}TerPP(CH_3)_2CHSn]_2$ (**Sn4**) in the crystal. Thermal ellipsoids are drawn at the 50% probability level (hydrogen atoms except H1 have been omitted for clarity). Selected bond lengths (Å) and angles (deg): Sn1–C1 2.296(3), Sn1•••P2 2.7250(6), P1–P2 2.1376(10), P1–C1 1.743(3), P2–C10 1.849(2), Sn1–C1–Sn1 93.54(12), C1–Sn1–C1 86.46(12), C1–Sn1–P2 70.17(7), Sn1–C1–P1 114.77(16).

Synthesis of ^{Mes}TerSn(IMe$_4$)CH$_2$P(CH$_3$)$_2$=P^{Mes}Ter (Sn5a)

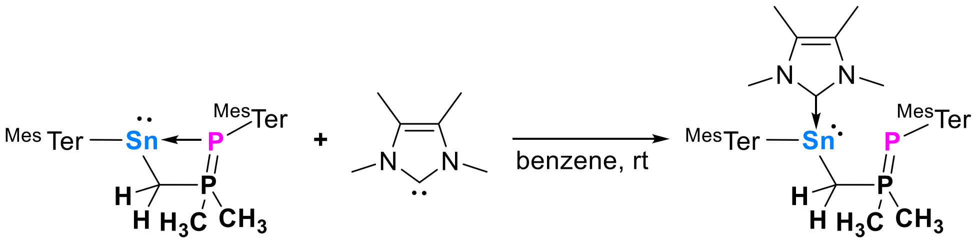

^{Mes}TerSn(hmds) (**Sn1a**) (0.042 g, 0.071 mmol) and ^{Mes}TerPP(CH$_3$)$_3$ (**P1b**) (0.030 g, 0.071 mmol) were dissolved in 0.6 mL of C_6D_6 and heated to 70 °C for 16 h for the *in-situ* generation of ^{Mes}TerSnCH$_2$P(CH$_3$)$_2$=P^{Mes}Ter (**Sn3a**). IMe$_4$ (0.009 g, 0.071 mmol) in 0.1 mL of C_6D_6 was added which results in a slight but observable brightening of the reaction mixture. Subsequent measurement of the ^{1}H and ^{31}P NMR spectra revealed clean conversion to ^{Mes}TerSn(IMe$_4$)CH$_2$P(CH$_3$)$_2$=P^{Mes}Ter (**Sn5a**). NMR spectra were collected at this point. All volatiles were removed under vacuum to obtain **Sn5a** as a bright yellow solid. Crystals suitable for single crystal X-ray diffraction were obtained from a saturated *n*-hexane solution of **Sn5a** at -30 °C, however did not allow for discussion of the structural parameters, thus only confirming the connectivity.

Yield: 0.036 g (0.037 mmol; 52%).
^{1}H NMR (400 MHz, C_6D_6, 298 K): δ = -1.00-(-0.91) (m, 1H, CH$_2$), 0.62-0.67 (m, 1H, CH$_2$), 0.74 (d, $^2J_{P,H}$ = 11.9 Hz, 3H, P(CH$_3$)$_2$), 1.16 (d, $^2J_{P,H}$ = 11.4 Hz, 3H, P(CH$_3$)$_2$), 1.59 (s, 6H, NC$_q$CH$_3$), 1.90 (s, 6H, CH$_3$), 2.21 (s, 6H, CH$_3$), 2.16 (s, 6H, CH$_3$), 2.25 (s, 6H, CH$_3$), 2.28 (s, 6H, CH$_3$), 2.31 (s, 6H, CH$_3$), 2.96 (s, 6H, NCH$_3$), 6.50-6.51 (m, 2H, CH$_{Aryl}$), 6.76-6.78 (m, 4H, CH$_{Aryl}$), 6.83-6.84 (m, 2H, CH$_{Aryl}$), 6.86-6.88 (m, 2H, CH$_{Aryl}$), 6.91-6.92 (m, 2H, CH$_{Aryl}$), 6.98-7.01 (m, 1H, CH$_{Aryl}$), 7.18-7.21 (m, 1H, CH$_{Aryl}$) ppm.
^{13}C{^{1}H} NMR (126 MHz, C_6D_6, 298 K): δ = 8.8 (NC$_q$<u>C</u>H$_3$), 14.7 (dd, $J_{P,C}$ = 42.9 Hz, $J_{P,C}$ = 38.6 Hz, CH$_2$), 18.4 (dd, $J_{P,C}$ = 36.6 Hz, $J_{P,C}$ = 1.8 Hz, P(CH$_3$)$_2$), 21.11 (CH$_3$), 21.13 (CH$_3$), 21.3 (CH$_3$), 21.5 (CH$_3$), 21.57 (CH$_3$), 21.61 (CH$_3$), 22.3 (d, $J_{P,C}$ = 31.6 Hz, P(CH$_3$)$_2$), 35.4 (d, $J_{P,C}$ = 4.8 Hz, NCH$_3$), 124.3 (N<u>C</u>$_q$CH$_3$), 126.2 (CH$_{Aryl}$), 127.7 (CH$_{Aryl}$), 127.8 (CH$_{Aryl}$), 128.2 (CH$_{Aryl}$)*, 128.3 (CH$_{Aryl}$)*, 128.55 (CH$_{Aryl}$), 128.62 (CH$_{Aryl}$), 129.0 (CH$_{Aryl}$), 134.8 (C$_{q,Aryl}$), 135.0 (C$_{q,Aryl}$), 136.2 (C$_{q,Aryl}$), 136.26 (C$_{q,Aryl}$), 136.33 (C$_{q,Aryl}$), 136.5 (C$_{q,Aryl}$), 143.3 (C$_{q,Aryl}$), 144.4 (dd, $^1J_{P,C}$ = 68.1 Hz, $^2J_{P,C}$ = 10.4 Hz, C$_{q,Aryl}$P), 147.8 (dd, $J_{P,C}$ = 13.5 Hz, $J_{P,C}$ = 9.5 Hz, C$_{q,Aryl}$), 149.3 (C$_{q,Aryl}$), 161.3 (dd, $J_{P,C}$ = 8.8 Hz, $J_{P,C}$ = 4.3 Hz, C$_{q,Aryl}$), 172.2 (d, $J_{P,C}$ = 5.0 Hz, C$_{q,IMe4}$) ppm.
* = overlap with C_6D_6 signal and assigned by ^{1}H/^{13}C HSQC/HMBC
^{31}P{^{1}H} NMR (161 MHz, C_6D_6, 298 K): δ = -108.6 (d, $^1J_{P,P}$ = 537.5 Hz, Sn satellites: $J_{119/117Sn,P}$ = approx. 99.9 Hz, P^{Mes}Ter), 13.8 (d, $^1J_{P,P}$ = 537.5 Hz, Sn satellites: $J_{119/117Sn,P}$ = approx. 148.0 Hz, H$_2$CP(CH$_3$)$_2$) ppm.
^{31}P NMR (161 MHz, C_6D_6, 298 K): δ = -108.7 (dd, $^1J_{P,P}$ = 537.3 Hz, $J_{P,H}$ = 30.8 Hz, P^{Mes}Ter), 13.8 (dhept, $^1J_{P,P}$ = 537.3 Hz, $J_{P,H}$ = 11.8 Hz, H$_2$CP(CH$_3$)$_2$) ppm.
^{119}Sn{^{1}H} NMR (149 MHz, C_6D_6, 298 K): δ = -190.5 (dd, $J_{119Sn,P}$ = 151.3 Hz, $J_{119Sn,P}$ = 102.4 Hz) ppm.
EA: Anal. calcd. for $C_{58}H_{70}N_2P_2Sn$: C, 71.39; H, 7.23: N, 2.87; Found: C, 71.50; H, 7.63; N, 2.89.

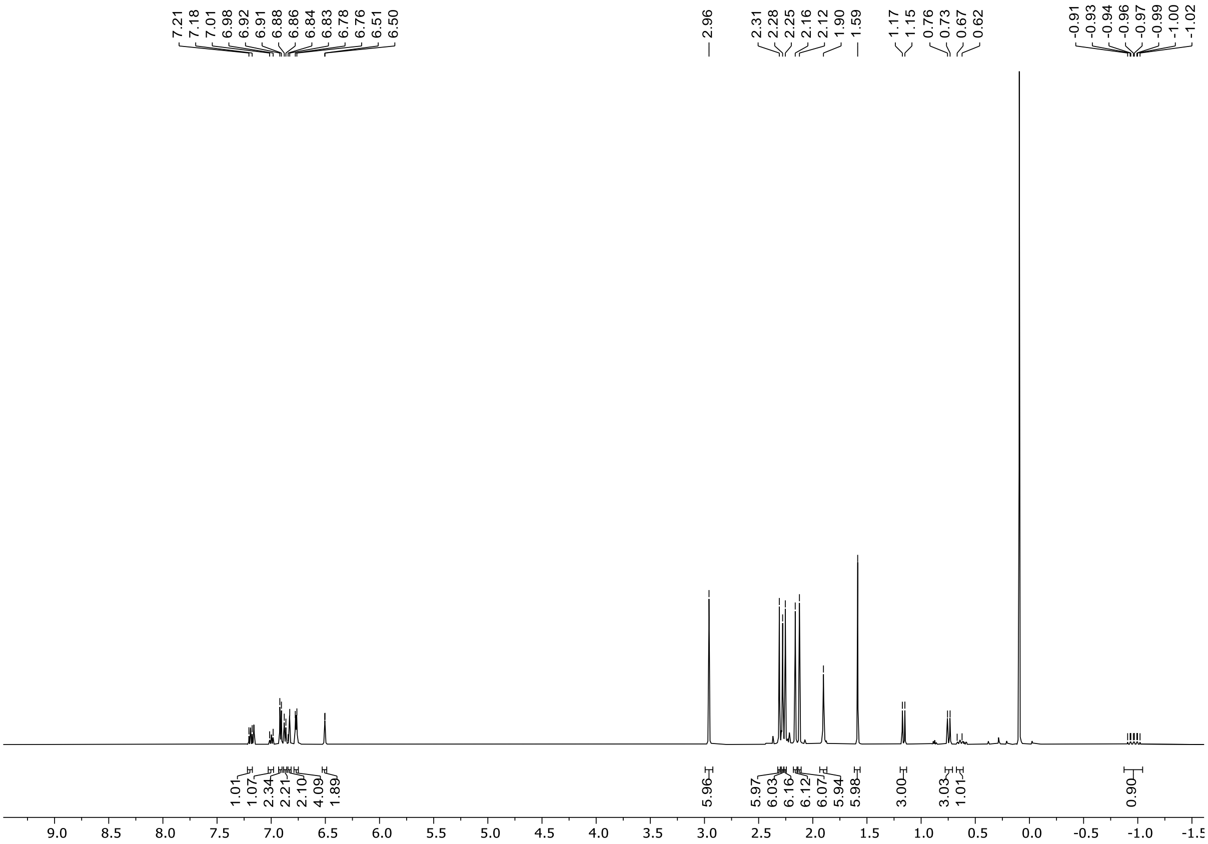

Figure S37. ^{1}H NMR spectrum of ^{Mes}TerSn(IMe$_4$)CH$_2$P(CH$_3$)$_2$=P^{Mes}Ter (**Sn5a**) (400 MHz, C_6D_6, 298 K); 0.10 ppm: hmds.

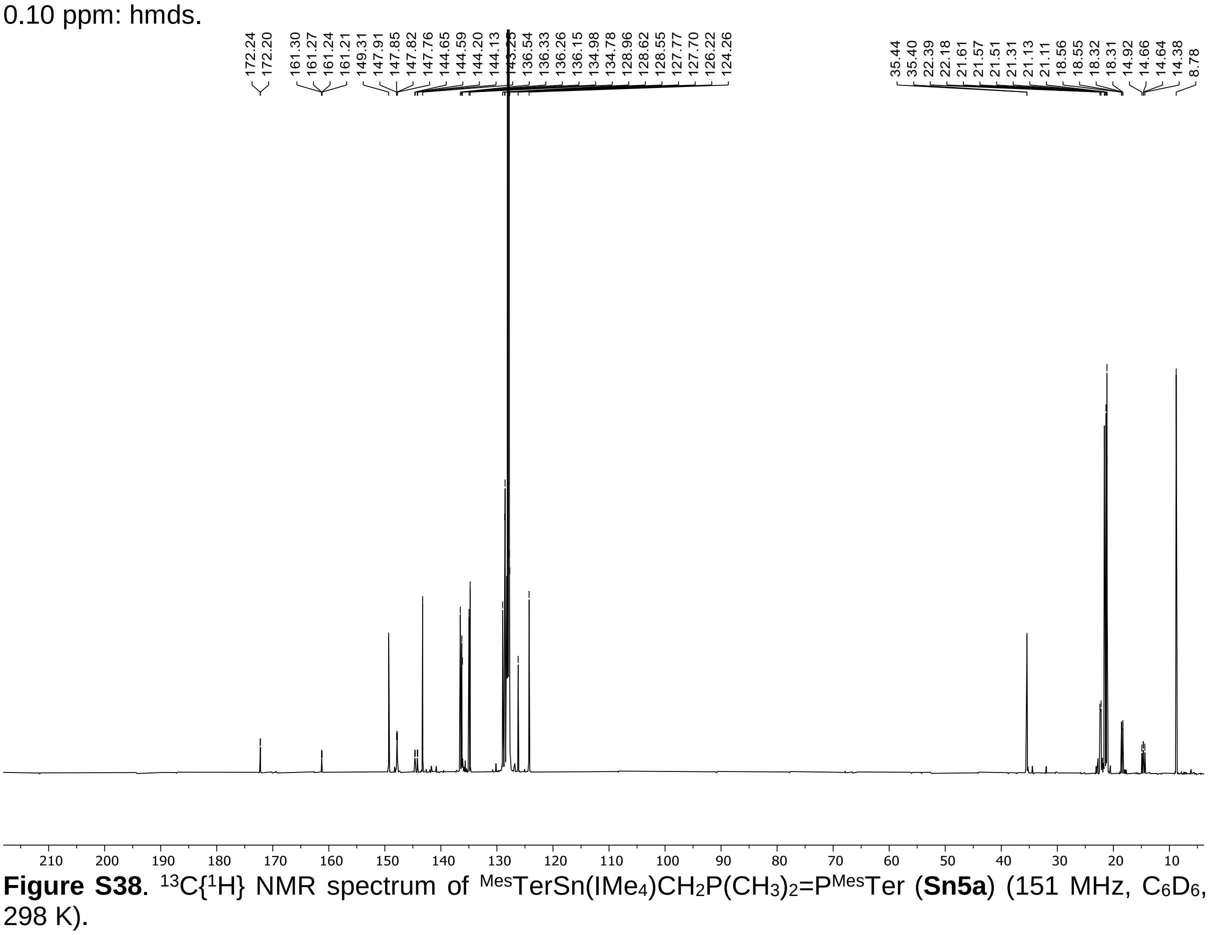

Figure S38. ^{13}C{^{1}H} NMR spectrum of ^{Mes}TerSn(IMe$_4$)CH$_2$P(CH$_3$)$_2$=P^{Mes}Ter (**Sn5a**) (151 MHz, C_6D_6, 298 K).

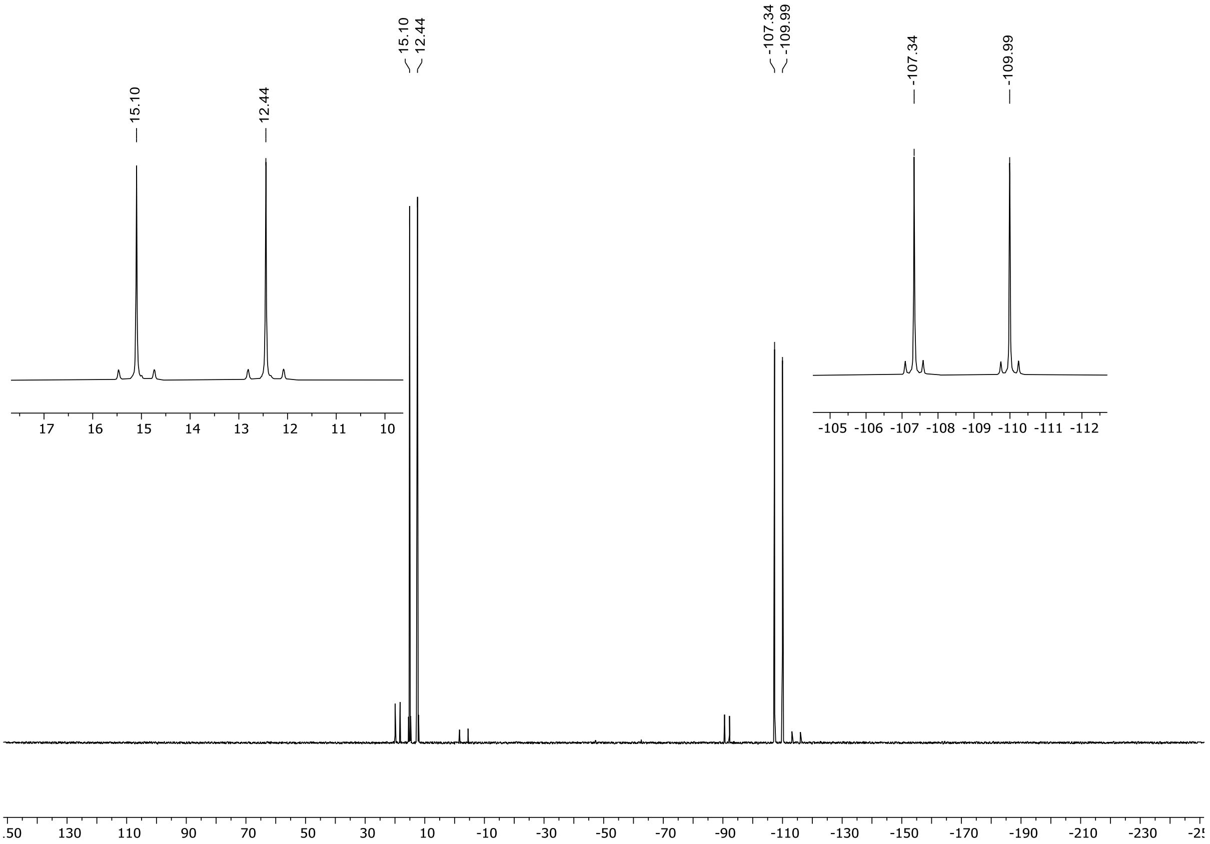

Figure S39. $^{31}P\{^{1}H\}$ NMR spectrum of $^{Mes}TerSn(IMe_4)CH_2P(CH_3)_2{=}P^{Mes}Ter$ (**Sn5a**) (161 MHz, C_6D_6, 298 K).

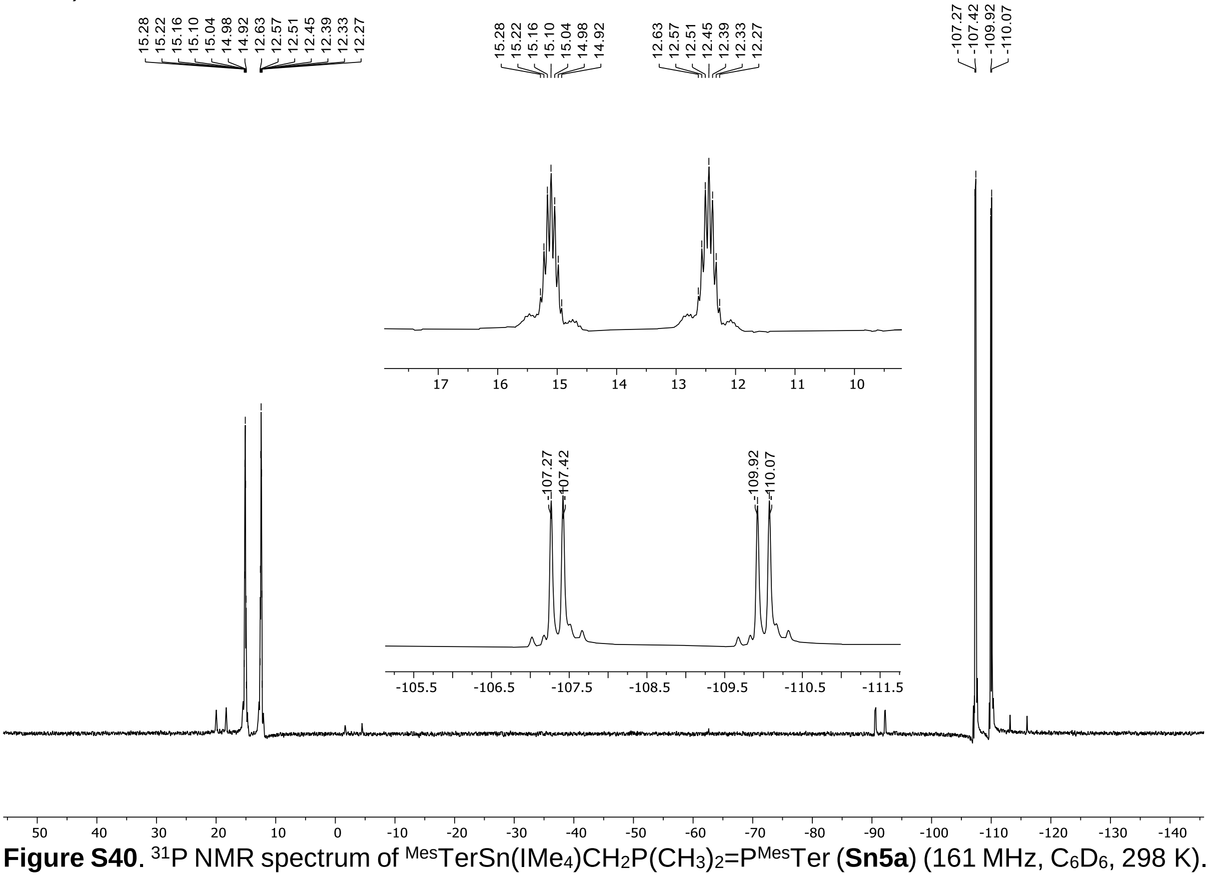

Figure S40. ^{31}P NMR spectrum of $^{Mes}TerSn(IMe_4)CH_2P(CH_3)_2{=}P^{Mes}Ter$ (**Sn5a**) (161 MHz, C_6D_6, 298 K).

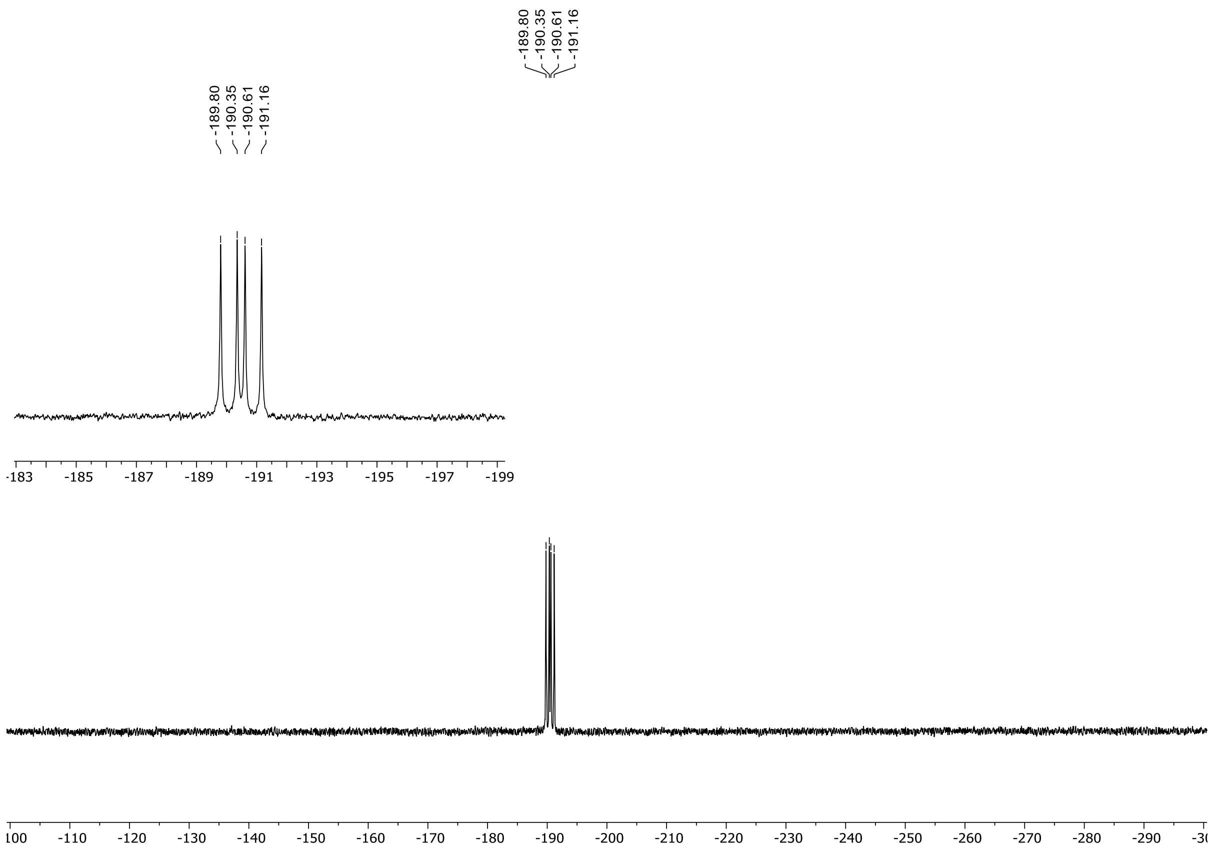

Figure S41. $^{119}Sn\{^{1}H\}$ NMR spectrum of $^{Mes}TerSn(IMe_4)CH_2P(CH_3)_2{=}P^{Mes}Ter$ (**Sn5a**) (149 MHz, C_6D_6, 298 K).

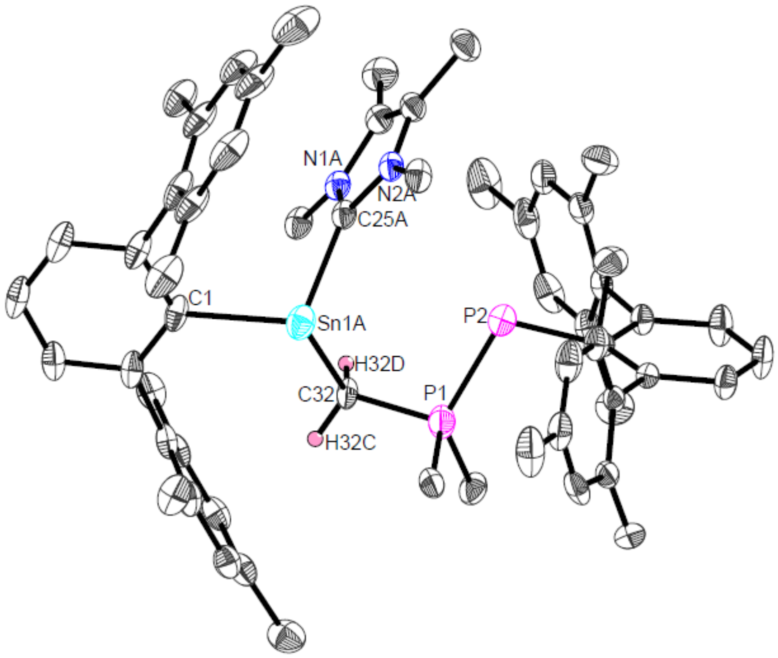

Figure S42. Molecular structure of $^{Mes}TerSn(IMe_4)CH_2P(CH_3)_2P{=}P^{Mes}Ter$ (**Sn5a**) in the crystal. Thermal ellipsoids are drawn at the 50% probability level (hydrogen atoms except H32C and H32D have been omitted for clarity).

Note: This structure is modulated. Some first order satellites are observable, however they have no meaningful intensities. The main molecule is modulated and the solvent disordered. While the modulation function for Sn can be readily modelled, the modulation functions for some of the mesityl groups are complicated. Unfortunately, it was not possible to find a satisfactory physically sensible solution. Here attempts were made to model the modulation as disorder to provide proof of connectivity only. Poor ellipsoid shapes are the results of the untreated modulation.

Synthesis of ^{Dipp}TerSn(IMe$_4$)CH$_2$P(CH$_3$)$_2$=PMes* (Sn5b)

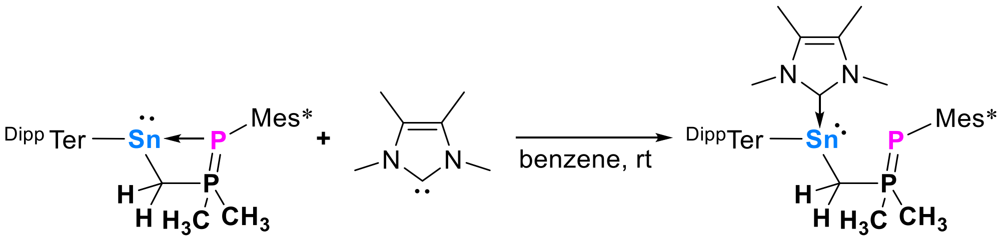

^{Dipp}TerSnCH$_2$P(CH$_3$)$_2$=PMes* (**Sn3b**) (0.035 g, 0.040 mmol) was dissolved in 0.5 mL of benzene followed by addition of a solution of IMe$_4$ (0.005 g, 0.040 mmol) in 0.1 mL of benzene. All volatiles were removed under vacuum, the yellow solid was subsequently dissolved in 0.6 ml of C_6D_6, and analysed by NMR spectroscopy which verified the formation of ^{Dipp}TerSn(IMe$_4$)CH$_2$P(CH$_3$)$_2$=PMes* (**Sn5b**). Listed below is the characteristic NMR data.

^{1}H NMR (400 MHz, C_6D_6, 298 K): δ = -1.00-(-0.91) (m, 1H, CH$_2$), 0.62-0.67 (m, 1H, CH$_2$), 0.74 (d, $^{2}J_{P,H}$ = 11.9 Hz, 3H, P(CH$_3$)$_2$), 1.16 (d, $^{2}J_{P,H}$ = 11.4 Hz, 3H, P(CH$_3$)$_2$), 1.59 (s, 6H, NC$_q$CH$_3$), 1.90 (s, 6H, CH$_3$), 2.21 (s, 6H, CH$_3$), 2.16 (s, 6H, CH$_3$), 2.25 (s, 6H, CH$_3$), 2.28 (s, 6H, CH$_3$), 2.31 (s, 6H, CH$_3$), 2.96 (s, 6H, NCH$_3$), 6.50-6.51 (m, 2H, CH$_{Aryl}$), 6.76-6.78 (m, 4H, CH$_{Aryl}$), 6.83-6.84 (m, 2H, CH$_{Aryl}$), 6.86-6.88 (m, 2H, CH$_{Aryl}$), 6.91-6.92 (m, 2H, CH$_{Aryl}$), 6.98-7.01 (m, 1H, CH$_{Aryl}$), 7.18-7.21 (m, 1H, CH$_{Aryl}$) ppm.
^{31}P{^{1}H} NMR (161 MHz, C_6D_6, 298 K): δ = -127.4 (d, $^{1}J_{P,P}$ = 537.7 Hz, Sn satellites: $J_{119/117Sn,P}$ = approx. 118.8 Hz, PMes*), 19.3 (d, $^{1}J_{P,P}$ = 537.3 Hz, Sn satellites: $J_{119Sn,P}$ = 149.5 Hz, $J_{117Sn,P}$ = 148.5 Hz, H$_2$CP(CH$_3$)$_2$) ppm.
^{31}P NMR (161 MHz, C_6D_6, 298 K): δ = -127.4 (dd, $^{1}J_{P,P}$ = 536.9 Hz, $J_{P,H}$ = 24.3 Hz, PMes*), 19.3 (dm, $^{1}J_{P,P}$ = 537.3 Hz, H$_2$CP(CH$_3$)$_2$) ppm.
^{119}Sn{^{1}H} NMR (149 MHz, C_6D_6, 298 K): δ = -181.4 (dd, $J_{119Sn,P}$ = 149.7 Hz, $J_{119Sn,P}$ = 121.7 Hz) ppm.

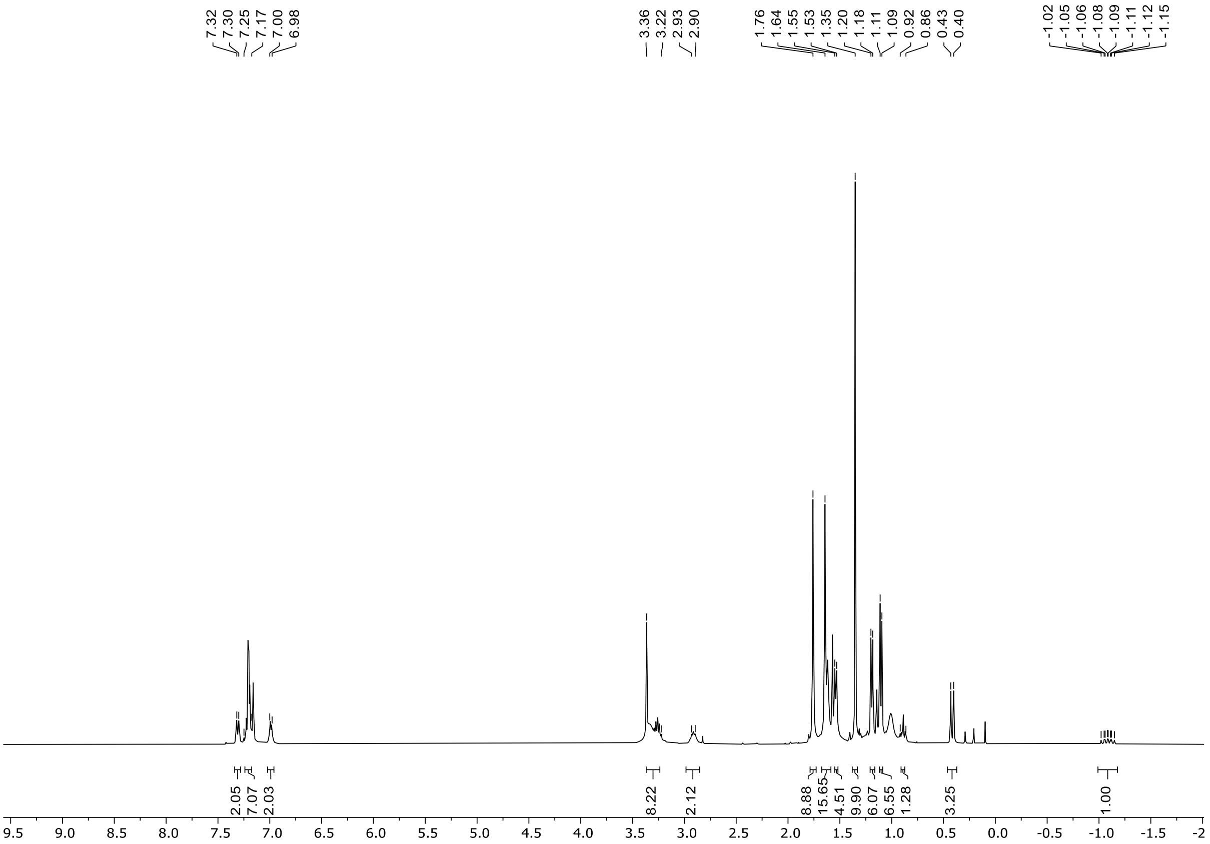

Figure S43. ^{1}H NMR spectrum of $^{Dipp}TerSn(IMe_4)CH_2P(CH_3)_2{=}PMes^*$ (**Sn5b**) (400 MHz, C_6D_6, 298 K).

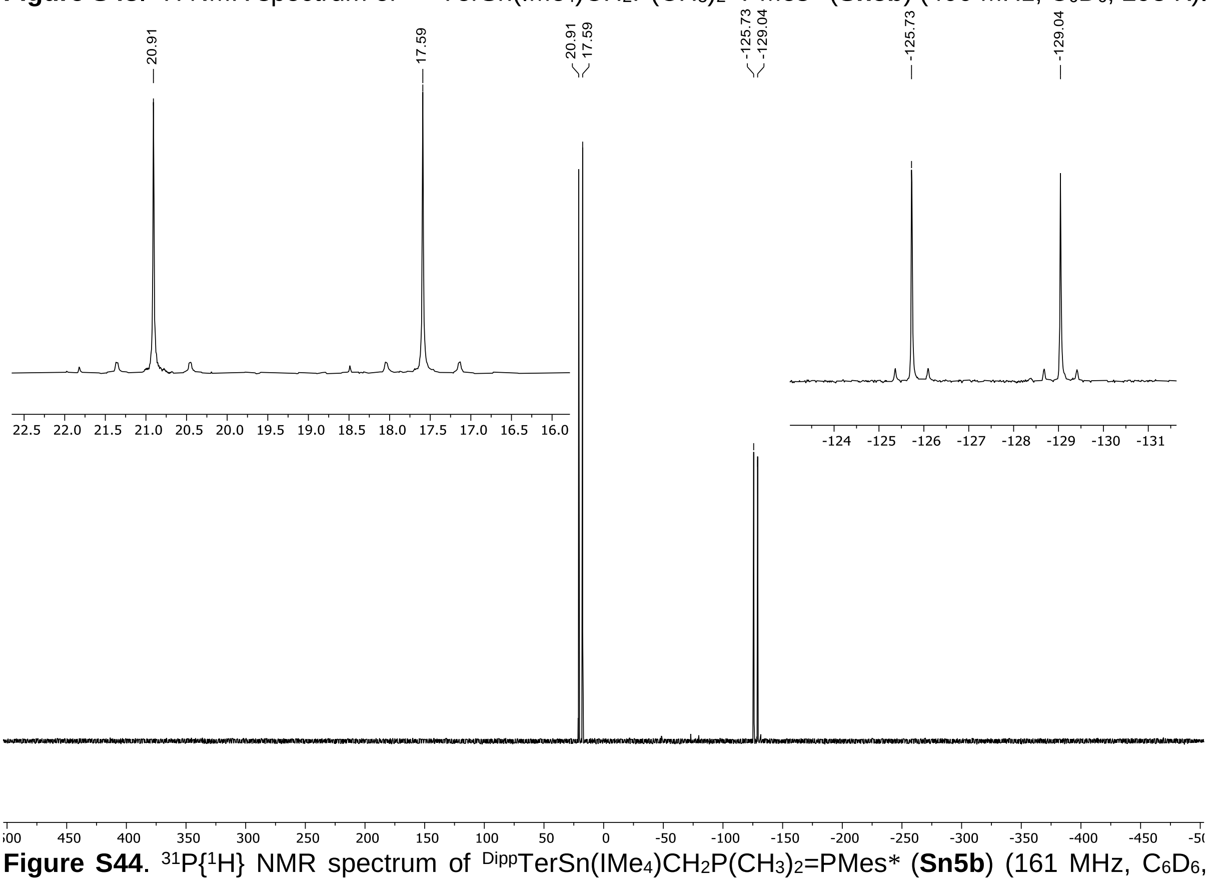

Figure S44. $^{31}P\{^{1}H\}$ NMR spectrum of $^{Dipp}TerSn(IMe_4)CH_2P(CH_3)_2{=}PMes^*$ (**Sn5b**) (161 MHz, C_6D_6, 298 K).

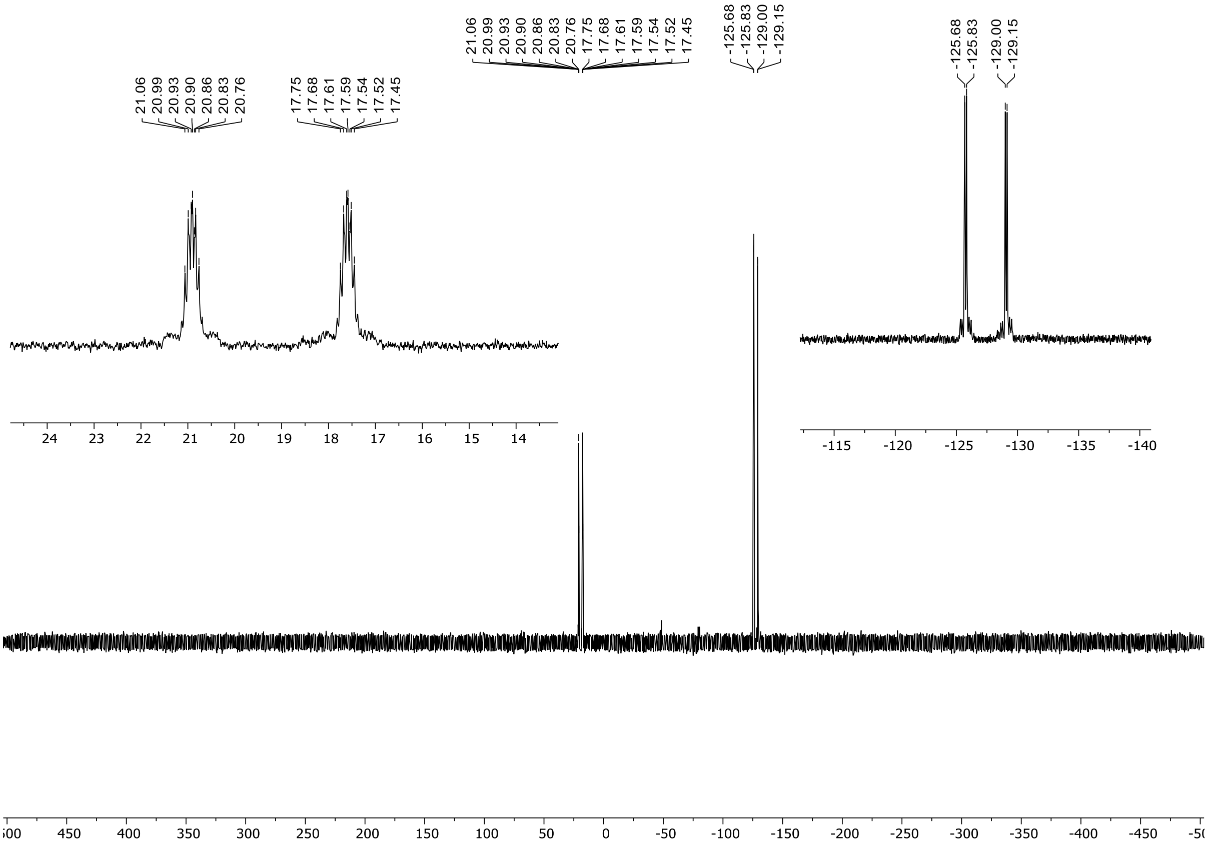

Figure S45. ^{31}P NMR spectrum of $^{Dipp}TerSn(IMe_4)CH_2P(CH_3)_2=PMes*$ (**Sn5b**) (161 MHz, C_6D_6, 298 K).

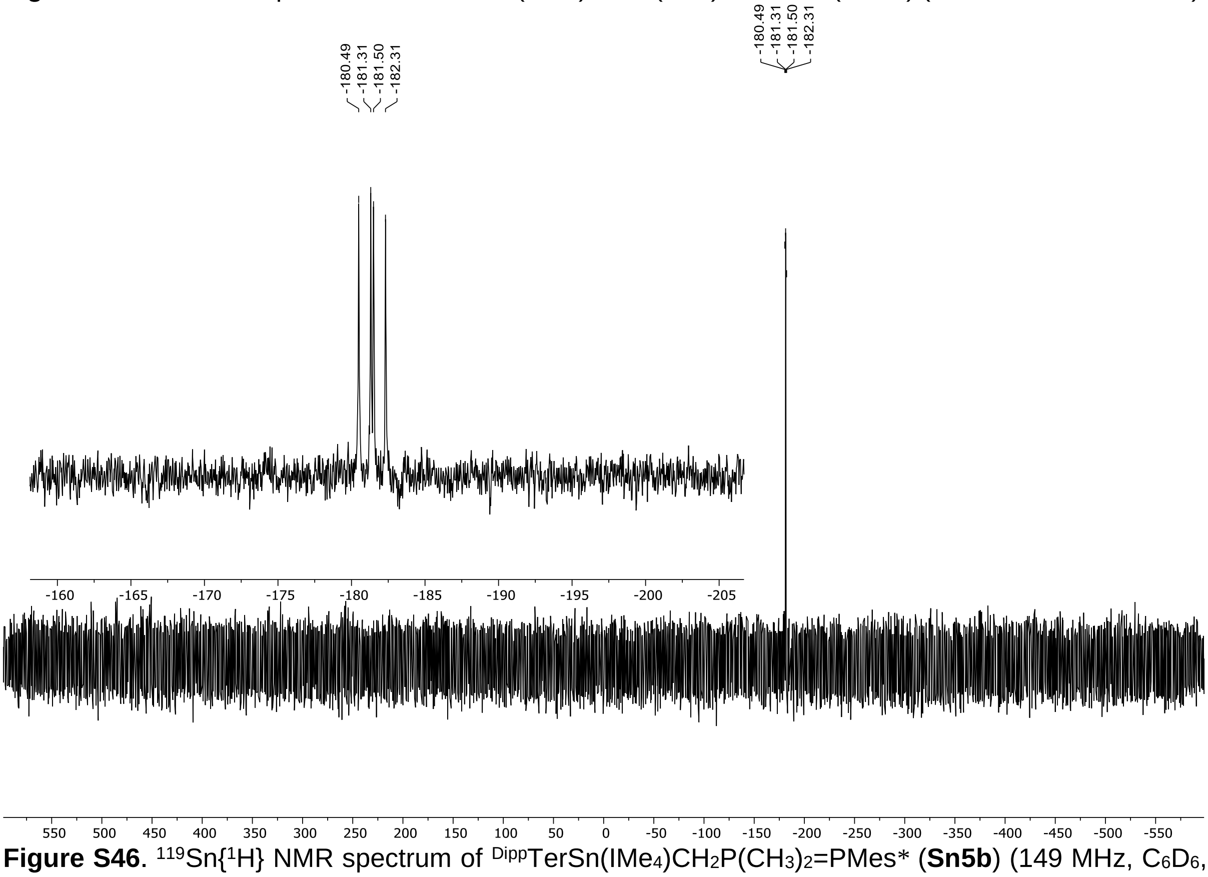

Figure S46. $^{119}Sn\{^1H\}$ NMR spectrum of $^{Dipp}TerSn(IMe_4)CH_2P(CH_3)_2=PMes*$ (**Sn5b**) (149 MHz, C_6D_6, 298 K).

Synthesis of $^{Dipp}TerSn(IMe_4)CH_2P(CH_3)_2{=}P^{Mes}Ter$ (Sn5c)

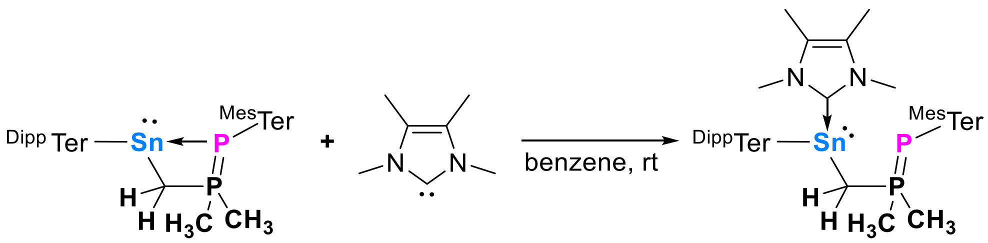

$^{Dipp}TerSn(hmds)$ (**Sn1b**) (0.040 g, 0.053 mmol) and $^{Mes}TerPP(CH_3)_3$ (**P1b**) (0.022 g, 0.053 mmol) were dissolved in 0.6 mL of C_6D_6 and heated to 60 °C for 16 h for the in-situ generation of $^{Dipp}TerSnCH_2P(CH_3)_2{=}P^{Mes}Ter$ (**Sn3c**). IMe_4 (0.007 g, 0.053 mmol) in 0.1 mL of C_6D_6 was added which results in a slight but observable brightening of the reaction mixture. The NMR data was collected at this point and the characteristic NMR data is listed below.
Crystals of **Sn5c** suitable for single crystal X-ray diffraction were obtained after removal of all volatiles under vacuum, addition of approx. 1 mL of *n*-pentane, subsequent filtration and final storage of the saturated solution at 4 °C.

^{1}H NMR (400 MHz, C_6D_6, 298 K): δ = -1.16-(-1.02) (m, 1H, CH_2), 0.26-0.37 (m(br), 6H, $CH(CH_3)_2$), 0.57-0.63 (m, $^2J_{P,H}$ = 11.4 Hz, 4H, $P(CH_3)_2$, CH_2), 0.97 (d, $^2J_{P,H}$ = 11.6 Hz, 3H, $P(CH_3)_2$), 1.06 (d, $^3J_{H,H}$ = 6.7 Hz, 6H, $CH(CH_3)_2$), 1.15 (d, $^3J_{H,H}$ = 6.7 Hz, 6H, $CH(CH_3)_2$), 1.45 (d, $^3J_{H,H}$ = 6.8 Hz, 6H, $CH(CH_3)_2$), 1.57 (s, 6H, NC_qCH_3), 2.21 (s, 6H, CH_3), 2.27 (s, 12H, CH_3), 2.80 (hept, $^3J_{H,H}$ = 6.7 Hz, 2H, $CH(CH_3)_2$), 2.99 (s, 6H, NCH_3), 3.15 (hept, $^3J_{H,H}$ = 6.8 Hz, 2H, $CH(CH_3)_2$), 6.77-6.84 (m, 6H, CH_{Aryl}), 6.87-6.90 (m, 2H, CH_{Aryl}), 6.93-6.95 (m, 2H, CH_{Aryl}), 6.98-7.02 (m, 1H, CH_{Aryl}), 7.12-7.20 (m, 5H, CH_{Aryl})* ppm.
* = overlapt with C_6D_5H signal
^{31}P{^{1}H} NMR (161 MHz, C_6D_6, 298 K): δ = -108.1 (d, $^1J_{P,P}$ = 539.8 Hz, Sn satellites: $J_{119/117Sn,P}$ = approx. 50.5 Hz, $P^{Mes}Ter$), 15.0 (d, $^1J_{P,P}$ = 539.9 Hz, Sn satellites: $J_{119/117Sn,P}$ = approx. 104.9 Hz, $H_2CP(CH_3)_2$) ppm.
^{31}P NMR (161 MHz, C_6D_6, 298 K): δ = -108.1 (dd, $^1J_{P,P}$ = 539.8 Hz, $J_{P,H}$ = 26.2 Hz, $P^{Mes}Ter$), 19.3 (dhept, $^1J_{P,P}$ = 539.9 Hz, $J_{P,H}$ = 11.1 Hz, $H_2CP(CH_3)_2$) ppm.
^{119}Sn{^{1}H} NMR (149 MHz, C_6D_6, 298 K): δ = -185.6 (dd, $J_{119Sn,P}$ = 107.6 Hz, $J_{119Sn,P}$ = 52.5 Hz) ppm.

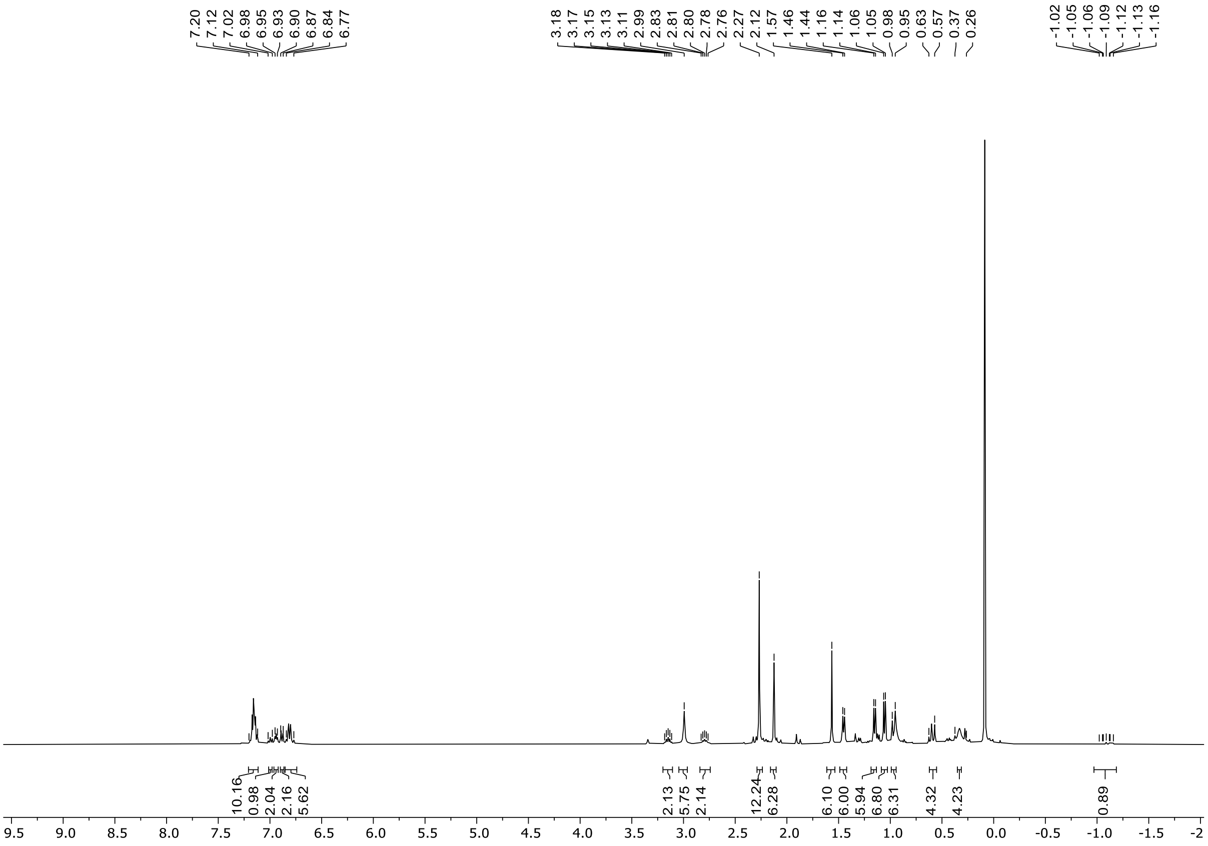

Figure S47. ^{1}H NMR spectrum of $^{Dipp}TerSn(IMe_4)CH_2P(CH_3)_2{=}P^{Mes}Ter$ (**Sn5c**) (400 MHz, C_6D_6, 298 K); 0.10 ppm: Hhmds.

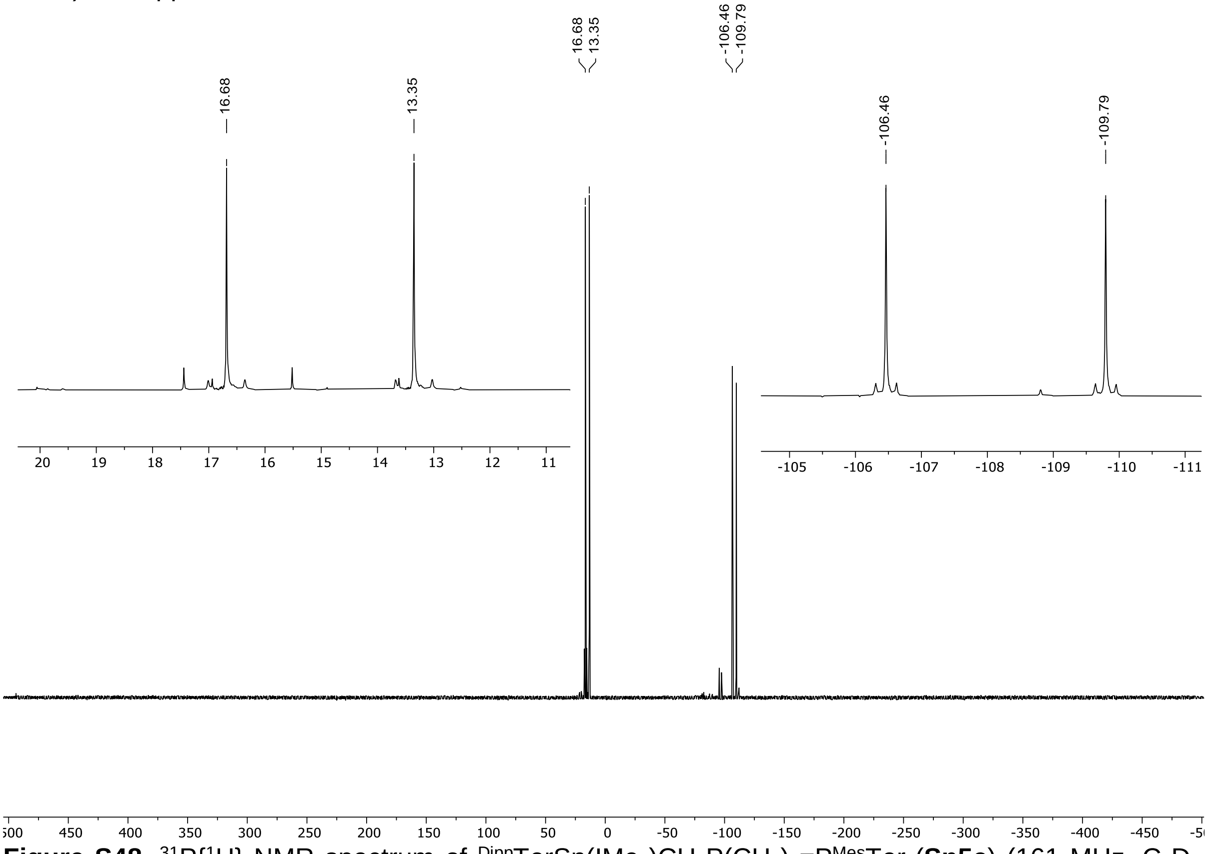

Figure S48. $^{31}P\{^1H\}$ NMR spectrum of $^{Dipp}TerSn(IMe_4)CH_2P(CH_3)_2{=}P^{Mes}Ter$ (**Sn5c**) (161 MHz, C_6D_6, 298 K).

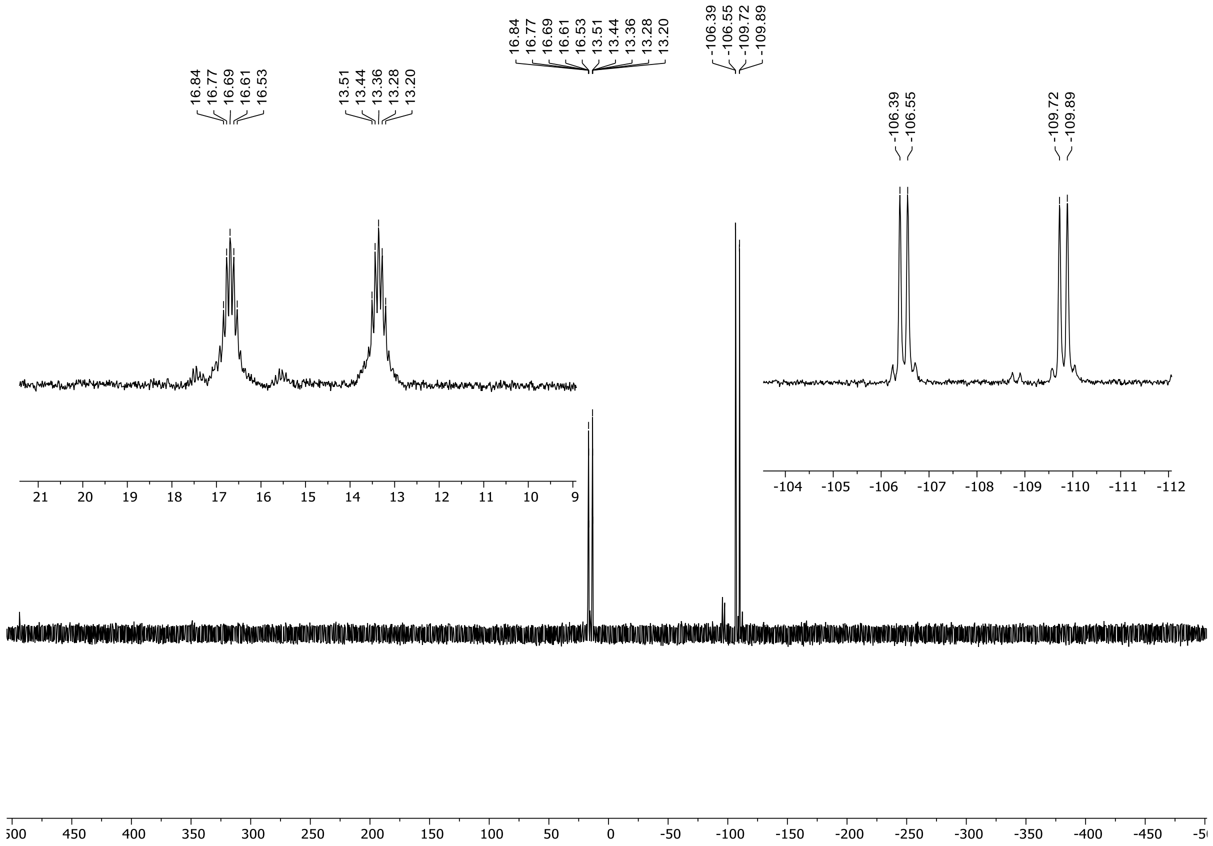

Figure S49. ^{31}P NMR spectrum of $^{Dipp}TerSn(IMe_4)CH_2P(CH_3)_2{=}P^{Mes}Ter$ (**Sn5c**) (161 MHz, C_6D_6, 298 K).

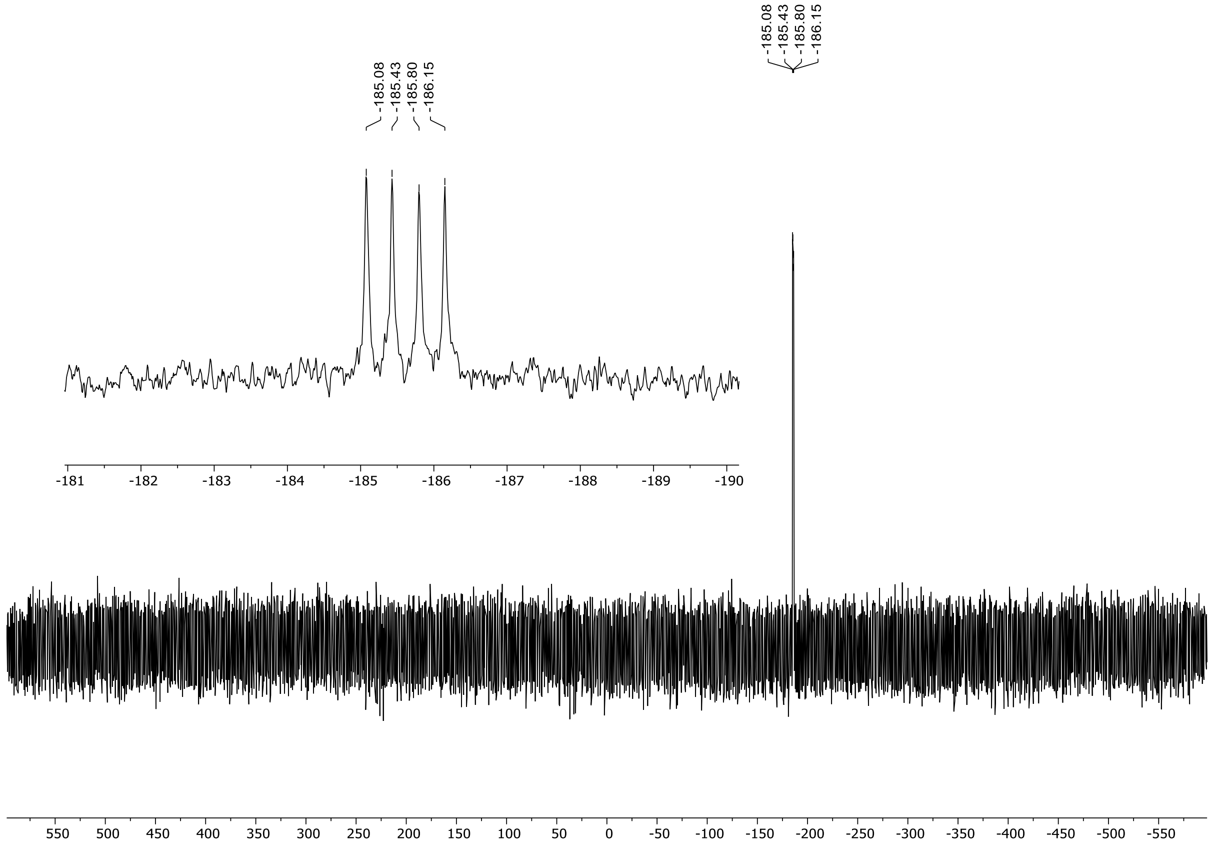

Figure S50. $^{119}Sn\{^1H\}$ NMR spectrum of $^{Dipp}TerSn(IMe_4)CH_2P(CH_3)_2{=}P^{Mes}Ter$ (**Sn5c**) (149 MHz, C_6D_6, 298 K).

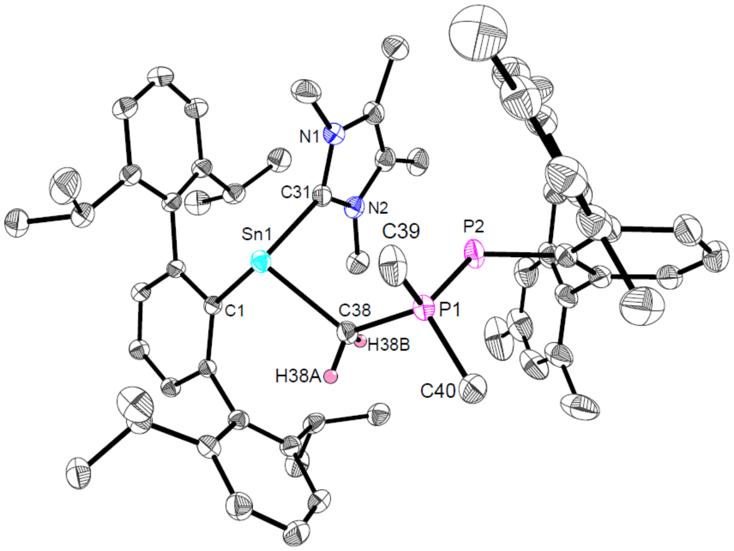

Figure S51. Molecular structure of ^{Dipp}TerSn(IMe$_4$)CH$_2$P(CH$_3$)$_2$=P^{Mes}Ter (**Sn5c**) in the crystal. Thermal ellipsoids are drawn at the 50% probability level (hydrogen atoms except H38A and H38B have been omitted for clarity). Selected bond lengths (Å) and angles (deg): Sn1•••P2 >4.7, Sn1–C1 2.254(3), Sn1–C38 2.264(3), Sn1–C31 2.274(3), P1–P2 2.1022(13), P1–C38 1.788(3), C1–Sn1–C38 101.40(10), C1–Sn1–C31 97.83(11).

Synthesis of ^{Dipp}TerSn(IMe$_4$)CH$_2$P(CH$_3$)$_2$=P^{Dipp}Ter (Sn5d)

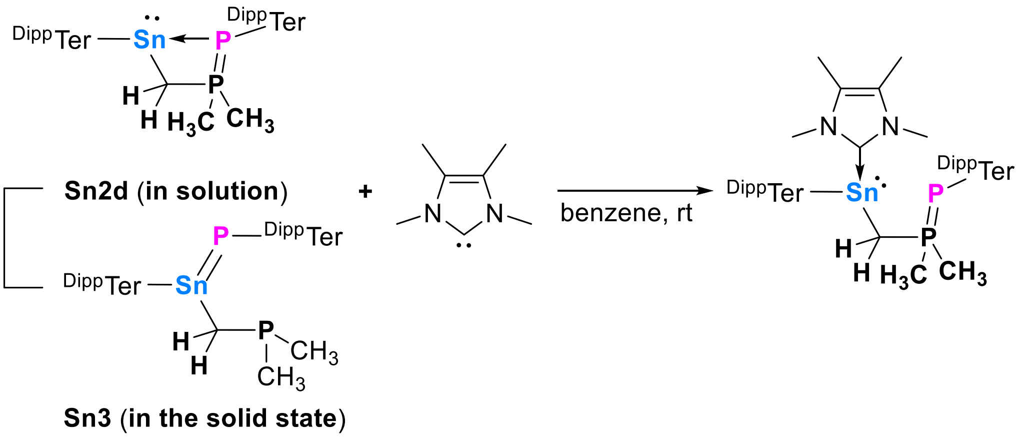

^{Dipp}TerSn(hmds) (**Sn1b**) (0.020 g, 0.026 mmol) and ^{Dipp}TerPP(CH$_3$)$_3$ (**P1c**) (0.013 g, 0.026 mmol) were dissolved in 0.6 mL of C_6D_6 and heated to 80 °C for a prolonged time for the in-situ generation of **Sn2d** / **Sn3**. IMe$_4$ (0.003 g, 0.026 mmol) in 0.1 mL of C_6D_6 was added which results in an immediate colour change of the reaction mixture from orange to bright yellow. All volatile components were removed under vacuum and the residue was recrystallized from *n*-hexane to give a bright yellow solid which was dissolved in 0.6 mL of C_6D_6. The NMR data was collected at this point and the characteristic NMR data is listed below.

^{1}H NMR (400 MHz, C_6D_6, 298 K): δ = -1.01-(-0.89) (m, 1H, CH$_2$), 0.58 (d, $^2J_{P,H}$ = 12.0 Hz, 3H, P(CH$_3$)$_2$), 0.93-0.93 (m, 3H, P(CH$_3$)$_2$), 1.01-1.02 (d, $^3J_{H,H}$ = 6.7 Hz, 6H, CH(C*H*$_3$)$_2$), 1.07-1.10 (m, 6H, CH(C*H*$_3$)$_2$), 1.12-1.17 (m, 21H, CH(C*H*$_3$)$_2$), 1.39 (d, $^3J_{H,H}$ = 6.9 Hz, 6H, CH(C*H*$_3$)$_2$), 1.43-1.47 (m, 9H, CH(C*H*$_3$)$_2$), 1.54 (s, 6H, NC$_q$CH$_3$), 2.76 (hept, $^3J_{H,H}$ = 6.7 Hz, 2H, C*H*(CH$_3$)$_2$), 2.90 (hept, $^3J_{H,H}$ = 6.9 Hz, 2H, C*H*(CH$_3$)$_2$), 3.06 (s, 6H, NCH$_3$), 3.19 (hept, $^3J_{H,H}$ = 6.8 Hz, 2H, C*H*(CH$_3$)$_2$), 3.35 (hept, $^3J_{H,H}$ = 6.8 Hz, 2H, C*H*(CH$_3$)$_2$), 6.87-6.89 (m, 1H, CH$_{Aryl}$), 7.01-7.02 (m, 2H, CH$_{Aryl}$), 7.06-7.12 (m, 6H, CH$_{Aryl}$), 7.13-7.14 (m, 3H, CH$_{Aryl}$), 7.17-7.26 (m, 6H, CH$_{Aryl}$) ppm.
^{31}P{^{1}H} NMR (161 MHz, C_6D_6, 298 K): δ = -109.3 (d, $^1J_{P,P}$ = 522.4 Hz, Sn satellites: $J_{119/117Sn,P}$ = approx. 71.8 Hz, P^{Dipp}Ter), 13.8 (d, $^1J_{P,P}$ = 522.3 Hz, Sn satellites: $J_{119/117Sn,P}$ = approx. 154.6 Hz, H$_2$CP(CH$_3$)$_2$) ppm.
^{31}P NMR (161 MHz, C_6D_6, 298 K): δ = -108.1 (dd, $^1J_{P,P}$ = 521.9 Hz, $J_{P,H}$ = 28.9 Hz, P^{Dipp}Ter), 13.8 (dm, $^1J_{P,P}$ = 522.0 Hz, H$_2$CP(CH$_3$)$_2$) ppm.
^{119}Sn{^{1}H} NMR (149 MHz, C_6D_6, 298 K): δ = -188.3 (dd, $J_{119Sn,P}$ = 159.2 Hz, $J_{119Sn,P}$ = 74.6 Hz) ppm.

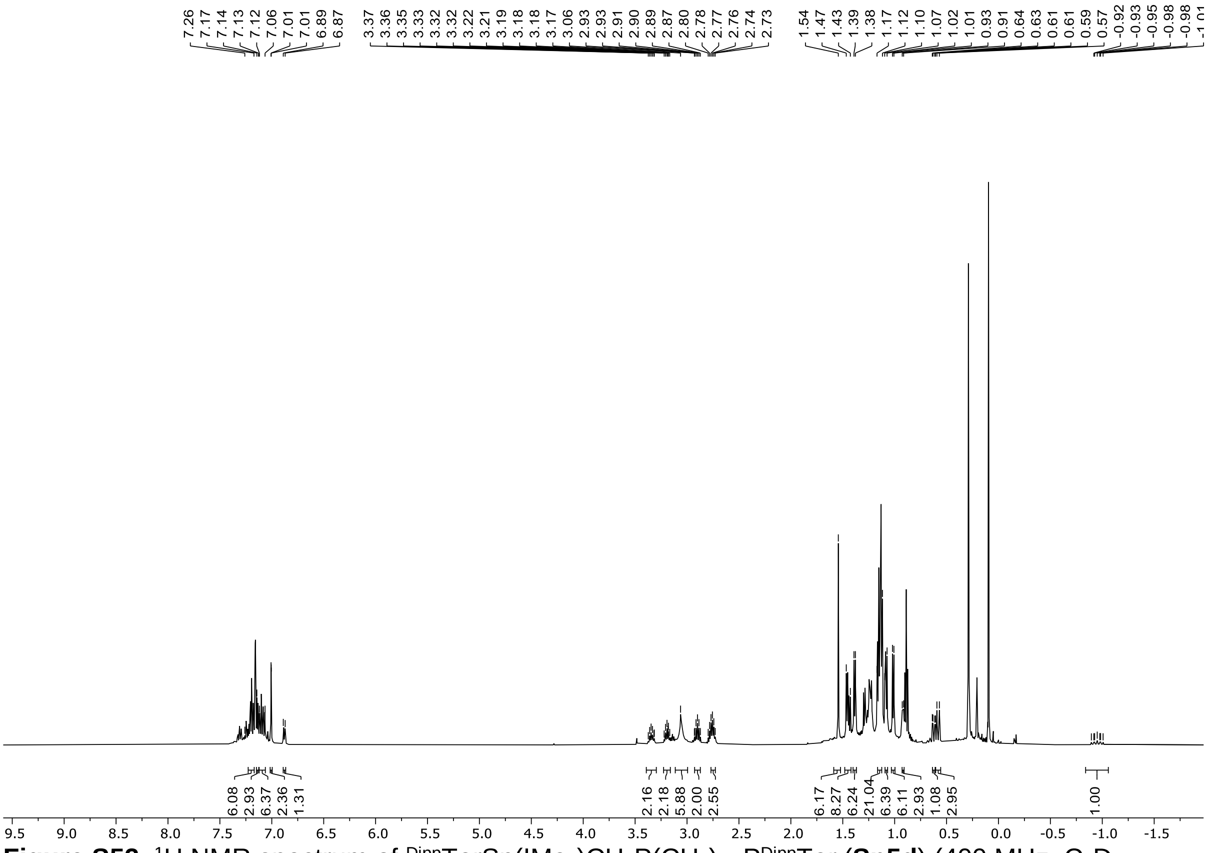

Figure S52. ^{1}H NMR spectrum of $^{Dipp}TerSn(IMe_4)CH_2P(CH_3)_2{=}P^{Dipp}Ter$ (**Sn5d**) (400 MHz, C_6D_6, 298 K); 0.10 ppm: Hhmds; 0.29 ppm: silicon grease.

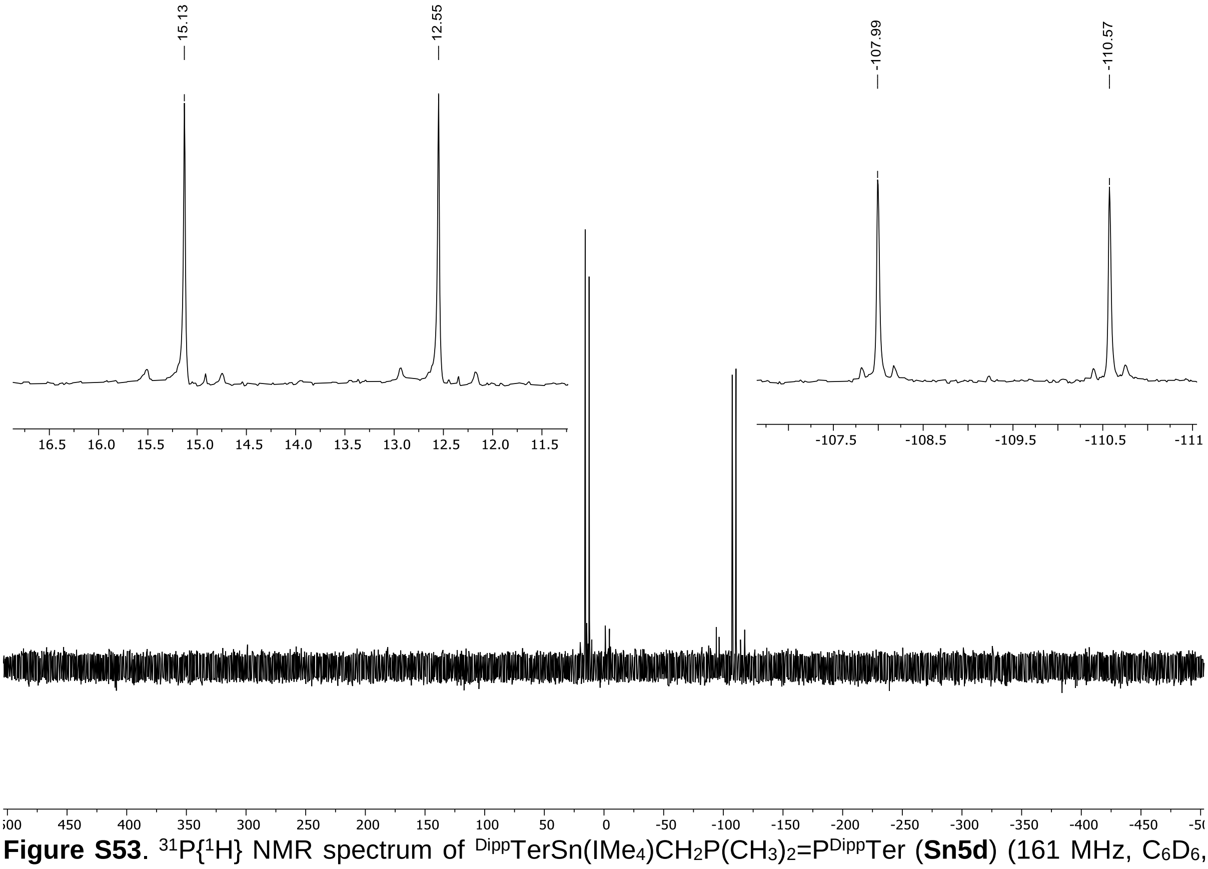

Figure S53. $^{31}P\{^{1}H\}$ NMR spectrum of $^{Dipp}TerSn(IMe_4)CH_2P(CH_3)_2{=}P^{Dipp}Ter$ (**Sn5d**) (161 MHz, C_6D_6, 298 K).

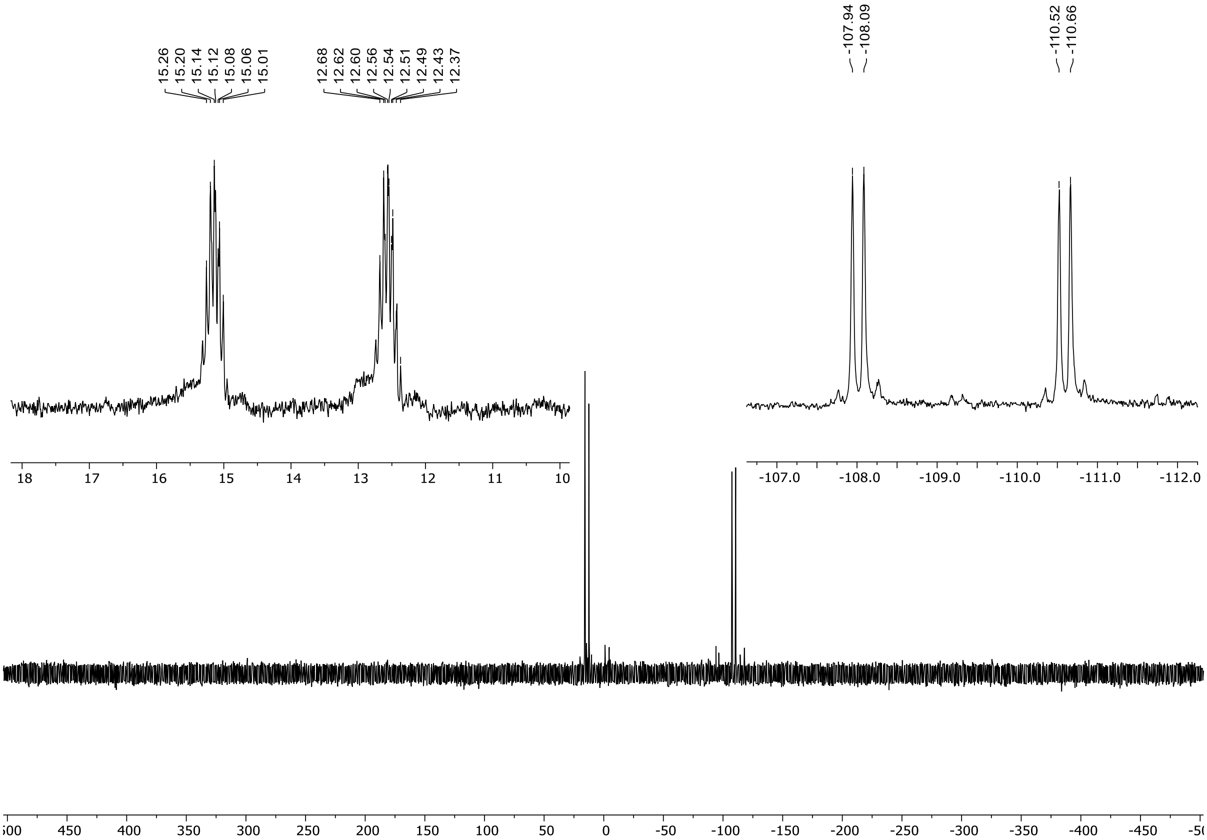

Figure S54. ^{31}P NMR spectrum of ^{Dipp}TerSn(IMe$_4$)CH$_2$P(CH$_3$)$_2$=P^{Dipp}Ter (**Sn5d**) (161 MHz, C_6D_6, 298 K).

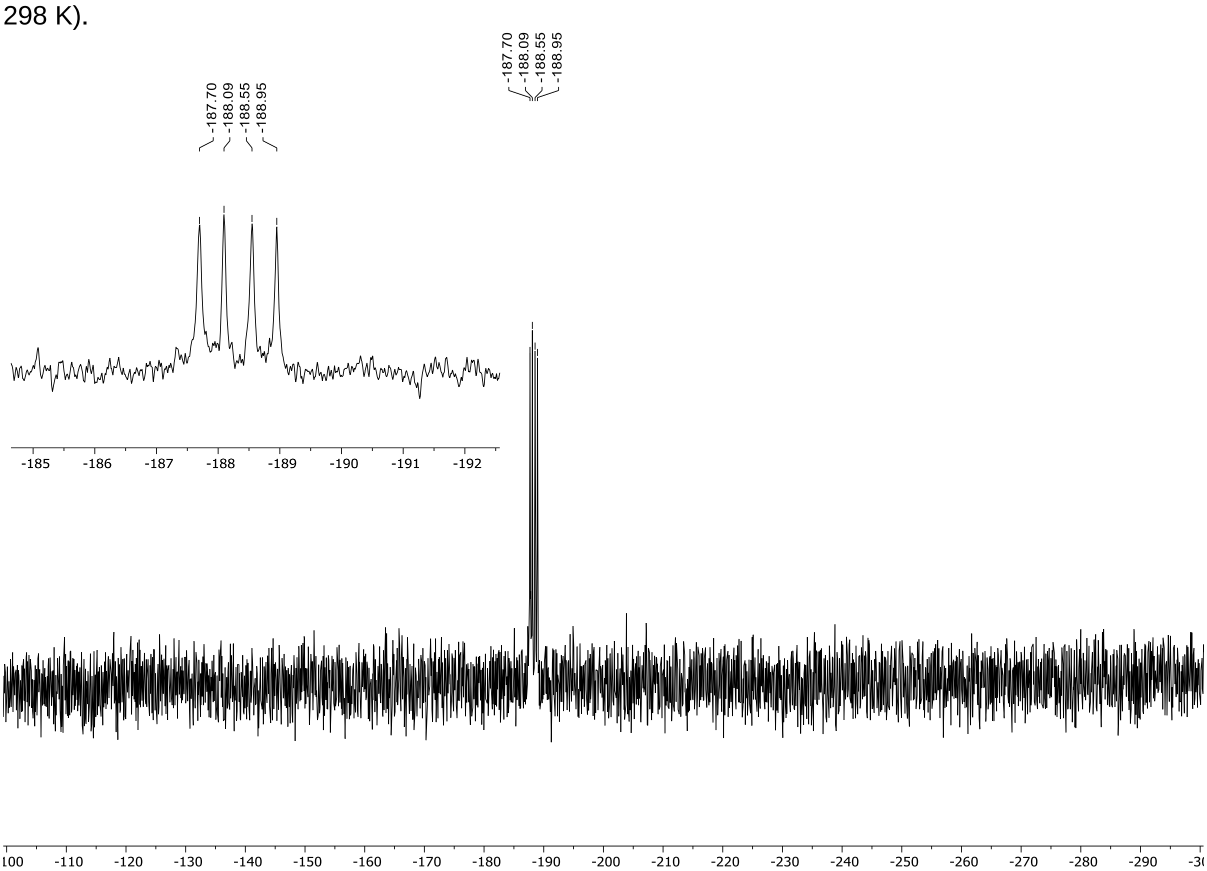

Figure S55. ^{119}Sn{^{1}H} NMR spectrum of ^{Dipp}TerSn(IMe$_4$)CH$_2$P(CH$_3$)$_2$=P^{Dipp}Ter (**Sn5d**) (149 MHz, C_6D_6, 298 K).

Reaction of Sn2d / Sn3 with H_2

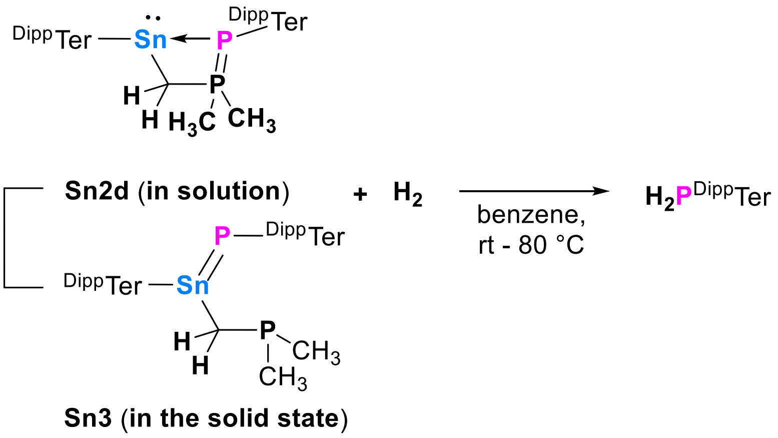

Sn2d / Sn3 (0.025 g, 0.025 mmol) was dissolved in 0.6 mL of C_6D_6 and transferred into a Young NMR tube. The solution was freeze-pump-thaw degassed three times and subsequently 1 bar of H_2 was added. The reaction was monitored by $^{31}P\{^1H\}$ NMR spectroscopy which revealed no reaction after 16 h at room temperature. The reaction mixture was slowly heated to 60 °C resulting in the slow formation of $H_2P^{Dipp}Ter$ and unidentified byproducts. The temperature was increased to 80 °C until all starting material was consumed. The NMR data of $H_2{}^{Dipp}Ter$ are in accordance to the literature.[S4]

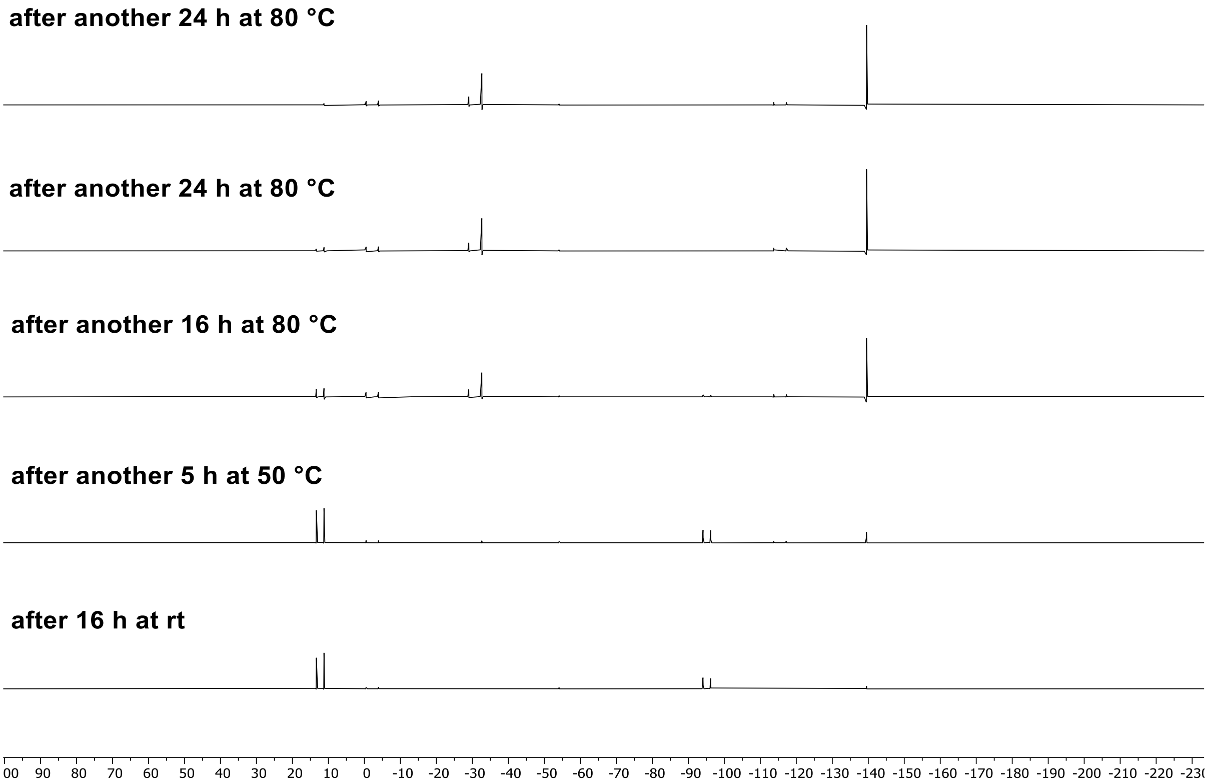

Figure S56. Monitoring of the reaction of $^{Dipp}TerSn(CH_2P(CH_3)_2)=P^{Dipp}Ter$ (**Sn2d**) / $^{Dipp}TerSn(CH_2P(CH_3)_2)=P^{Dipp}Ter$ (**Sn3**) with H_2 via $^{31}P\{^1H\}$ NMR spectroscopy (161 MHz, C_6D_6, 298 K).

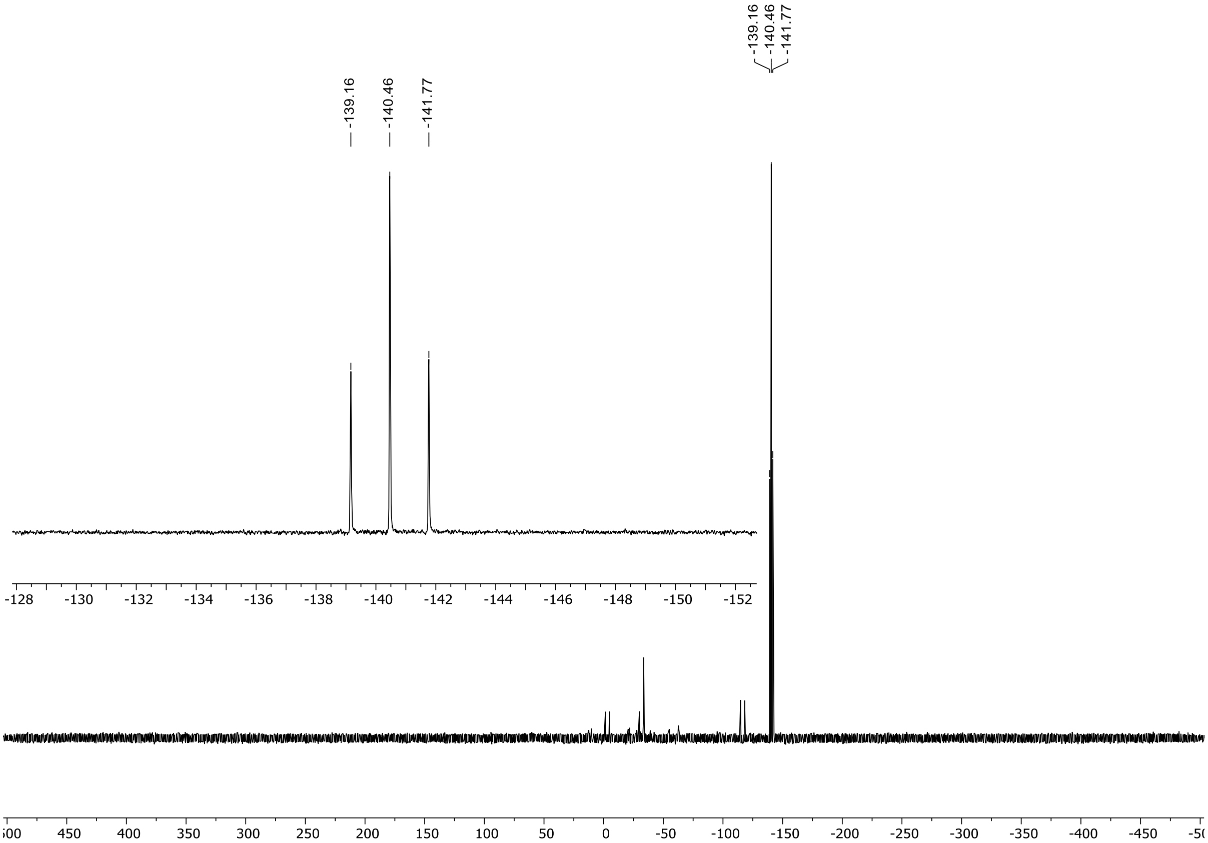

Figure S57. ^{31}P NMR spectrum after the reaction of ^{Dipp}TerSn($CH_2P(CH_3)_2$)=P^{Dipp}Ter (**Sn2d**) / ^{Dipp}TerSn($CH_2P(CH_3)_2$)=P^{Dipp}Ter (**Sn3**) with H_2 (161 MHz, C_6D_6, 298 K).

Reaction of ^{Dipp}TerSn($CH_2P(CH_3)_2$)=PMes* (Sn2b) with H_2

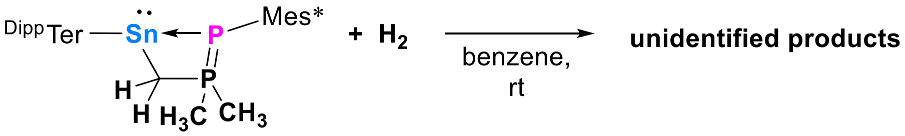

^{Dipp}TerSn($CH_2P(CH_3)_2$)=PMes* (**Sn2b**) (0.030 g, 0.035 mmol) was dissolved in 0.6 mL of C_6D_6 and transferred into a Young NMR tube. The solution was freeze-pump-thaw degassed three times and subsequently 1 bar of H_2 was added showing the formation of several unidentified products according to both ^{1}H and $^{31}P\{1H\}$ NMR spectroscopy). The corresponding spectra are shown below.

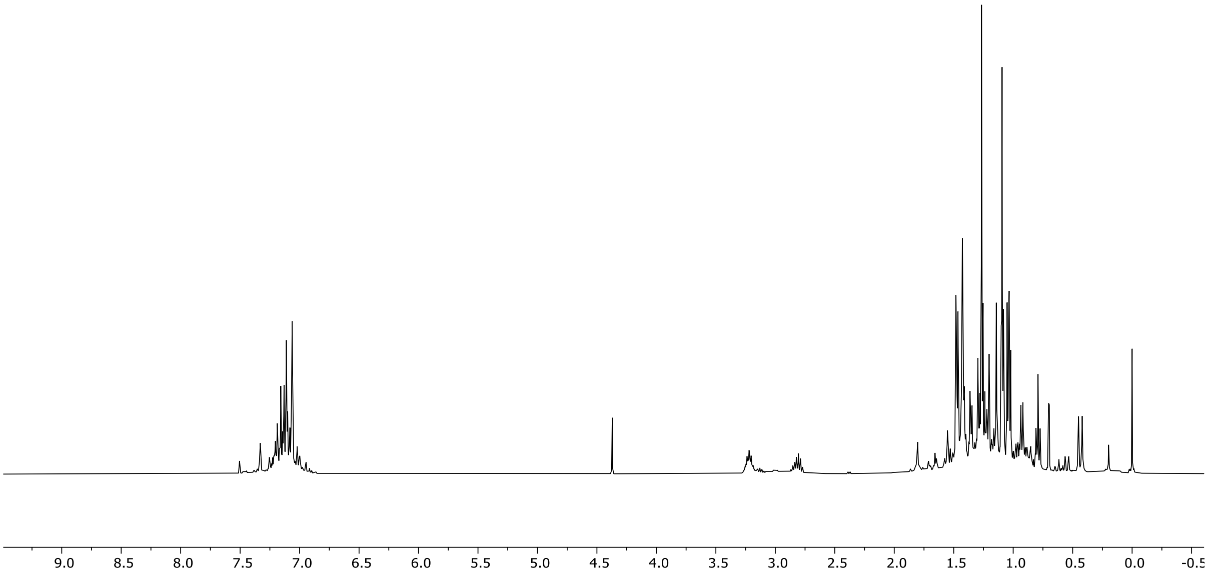

Figure S58. ^{1}H NMR spectrum after the addition of H_2 to ^{Dipp}TerSnCH$_2$P(CH_3)$_2$=PMes* (**Sn2b**) (400 MHz, C_6D_6, 298 K).

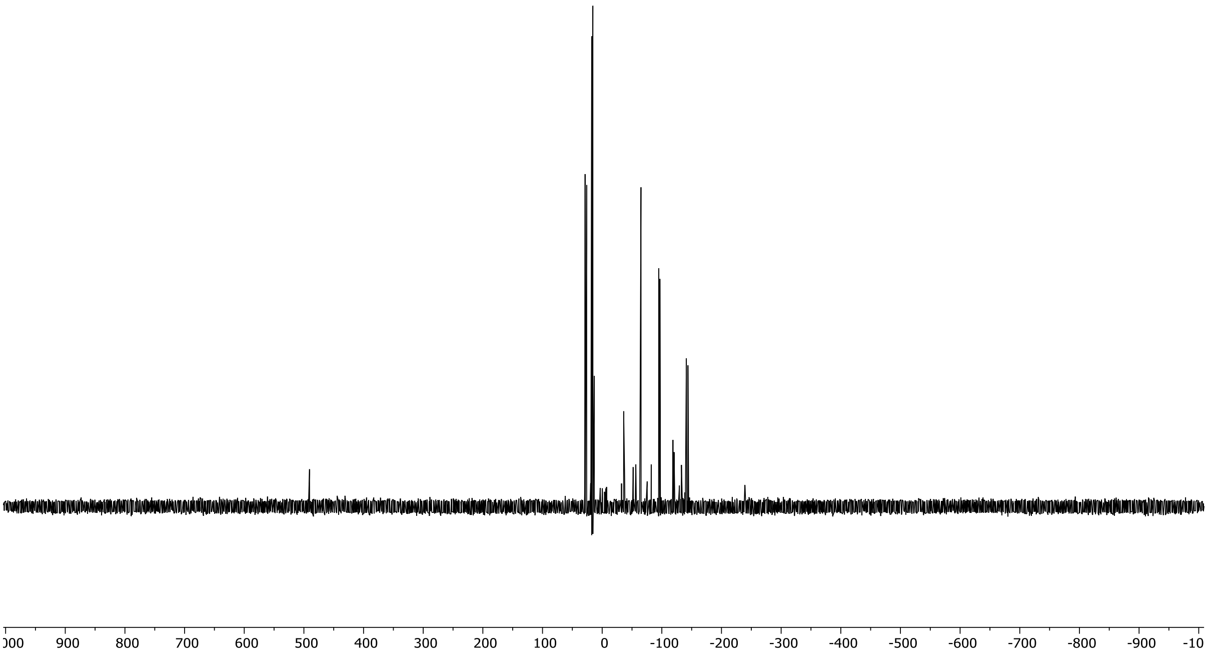

Figure S59. $^{31}P\{^{1}H\}$ NMR spectrum after the addition of H_2 to $^{Dipp}TerSnCH_2P(CH_3)_2{=}PMes^*$ (**Sn2b**) (161 MHz, C_6D_6, 298 K).

Reaction of $^{Dipp}TerSn(CH_2P(CH_3)_2)=P^{Dipp}Ter$ (Sn2d) / $^{Dipp}TerSn(CH_2P(CH_3)_2)=P^{Dipp}Ter$ (Sn3) with HCCPh

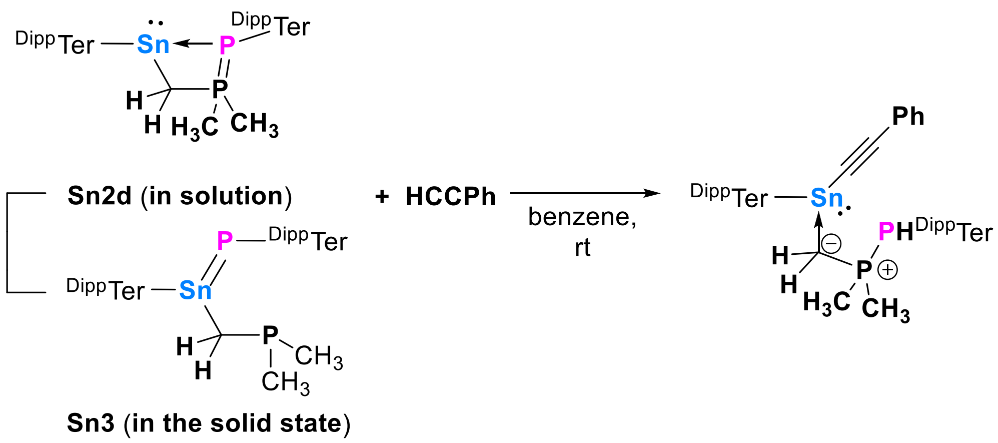

$^{Dipp}TerSn(CH_2P(CH_3)_2)=P^{Dipp}Ter$ (**Sn2d**) / $^{Dipp}TerSn(CH_2P(CH_3)_2)=P^{Dipp}Ter$ (**Sn3**) (0.040 g, 0.039 mmol) was dissolved in 0.6 mL of C_6D_6 followed by addition of phenylacetylene (0.004 g, 0.039 mmol) in 0.1 mL of C_6D_6.The reaction progress was monitored by $^{31}P\{^1H\}$ NMR spectroscopy until **Sn2d** / **Sn3** was completely consumed (Figure S60). Four new doublets are the main new signals. All volatiles were removed under vacuum. The remaining solid was suspended in 1 mL of *n*-hexane, filtered and stored at 4 °C which results in the formation of colourless uniform crystals which were suitable for single crystal X-ray diffraction revealing the formation of $^{Dipp}TerSn(CCPh)CH_2P(CH_3)_2P(H)^{Dipp}Ter$ (**Sn6**). The material was isolated and dissolved in C_6D_6. The corresponding characteristic NMR spectra and the most diagnostic signals are shown and listed below. Repeatedly, the ratio of the two new species in solution is approximately 1.0 (A): 0.6 (B) which we assign to the existence of diasteroisomers. **Note**: **Sn6** is not stable in aromatic hydrocarbons and decomposes over time.

Yield: 0.024 g (0.021 mmol, 54%).
^{1}H NMR (400 MHz, C_6D_6, 298 K): δ = -1.10-(-1.05) (m, 1H, CH_2^B), -0.63-(-0.57) (m, 1H, CH_2^A), 3.88 (dd, $^1J_{P,P}$ = 248.5 Hz, $^3J_{H,H}$ = 6.1 Hz, PH^B), 3.90 (dd, $^1J_{P,P}$ = 251.1 Hz, $^3J_{H,H}$ = 6.4 Hz, PH^A) ppm.
$^{31}P\{^1H\}$ NMR (161 MHz, C_6D_6, 298 K): δ = -73.4 (d, $^1J_{P,P}$ = 278.7 Hz, Sn satellites: $J_{119/117Sn,P}$ = approx. 106.2 Hz, HP^B), -72.8 (d, $^1J_{P,P}$ = 276.3 Hz, Sn satellites: $J_{119/117Sn,P}$ = approx. 61.8 Hz, HP^A), 21.7 (d, $^1J_{P,P}$ = 278.8 Hz, Sn satellites: $J_{119/117Sn,P}$ = approx. 33.9 Hz, HP^B), 23.2 (d, $^1J_{P,P}$ = 276.3 Hz, Sn satellites: $J_{119/117Sn,P}$ = approx. 95.6 Hz, HP^A) ppm.
^{31}P NMR (161 MHz, C_6D_6, 298 K): δ = -73.4 (dd, $^1J_{P,P}$ = 281.0 Hz, $^1J_{P,H}$ = 248.7 Hz, HP^B), -72.8 (dd, $^1J_{P,P}$ = 276.3 Hz, $^1J_{P,H}$ = 250.6 Hz, HP^A), 21.7 (dm, $^1J_{P,P}$ = 278.8 Hz, HP^B), 23.2 (dm, $^1J_{P,P}$ = 276.3 Hz, HP^A) ppm.
$^{119}Sn\{^1H\}$ NMR (149 MHz, C_6D_6, 298 K): δ = -206.5 (dd, $^2J_{Sn,P}$ = 98.5 Hz, $^3J_{Sn,P}$ = 63.2 Hz), -202.0 (dd, $^2J_{Sn,P}$ = 107.2 Hz, $^3J_{Sn,P}$ = 35.1 Hz) ppm.

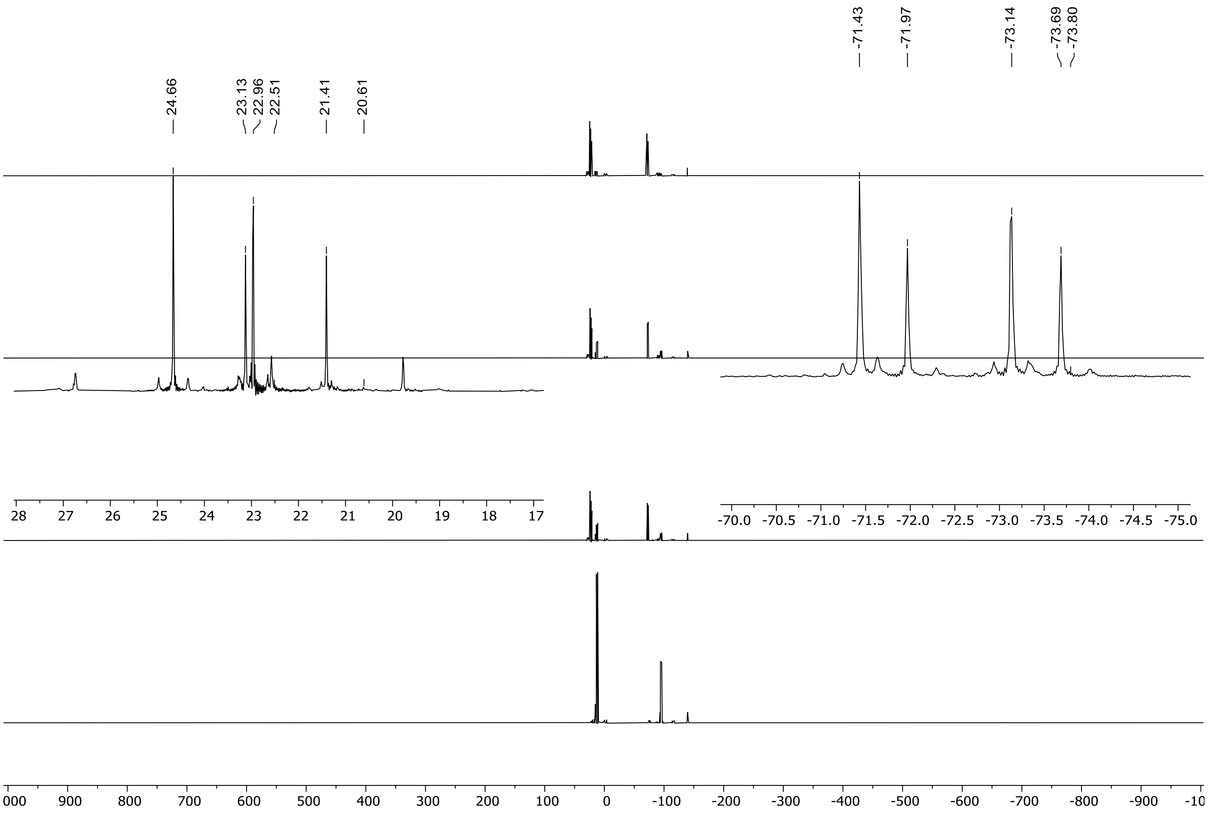

Figure S60. Monitoring of the reaction of ^{Dipp}TerSn(CH$_2$P(CH$_3$)$_2$)=P^{Dipp}Ter (**Sn2d**) / ^{Dipp}TerSn(CH$_2$P(CH$_3$)$_2$)=P^{Dipp}Ter (**Sn3**) with HCCPh via ^{31}P{^{1}H} NMR spectroscopy (161 MHz, C_6D_6, 298 K).

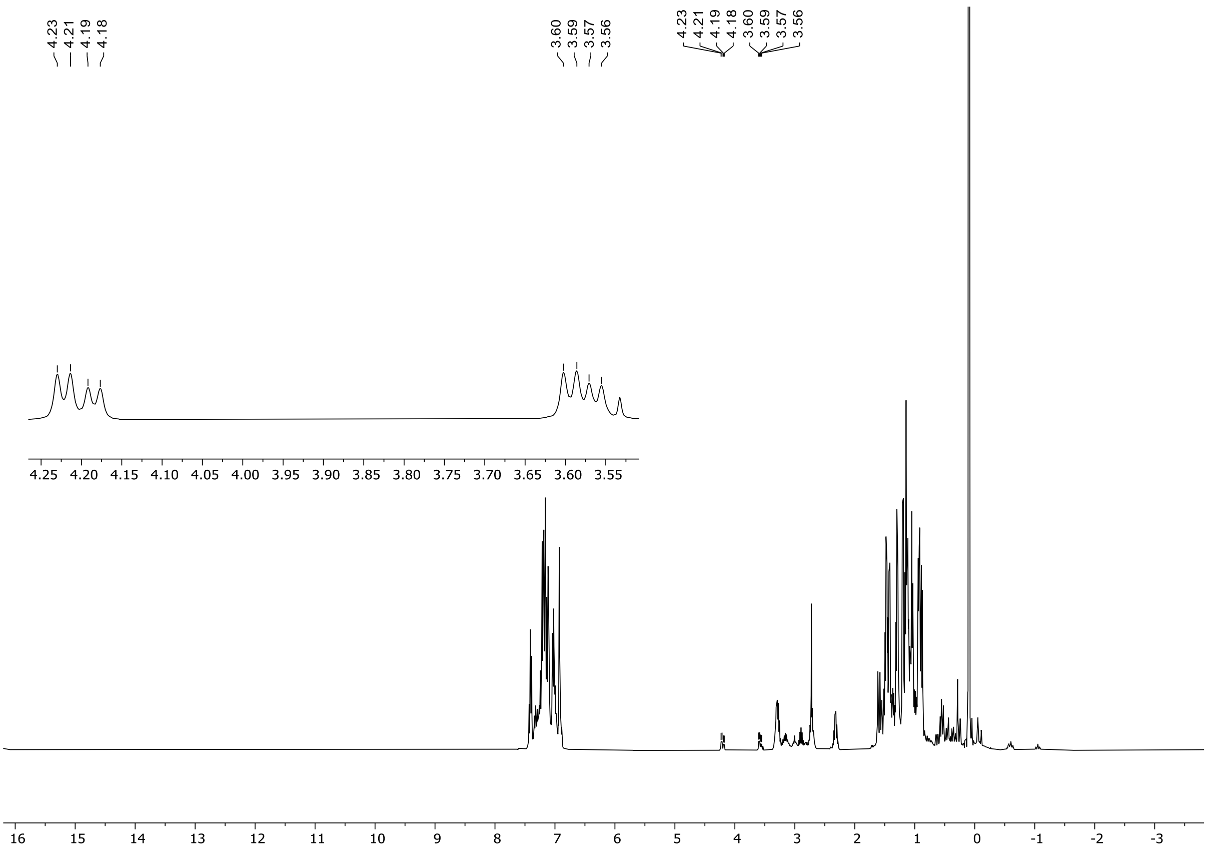

Figure S61. ^{1}H NMR spectrum of the crystalline material after the reaction of ^{Dipp}TerSn(CH$_2$P(CH$_3$)$_2$)=P^{Dipp}Ter (**Sn2d**) / ^{Dipp}TerSn(CH$_2$P(CH$_3$)$_2$)=P^{Dipp}Ter (**Sn3**) with HCCPh (400 MHz, C$_6$D$_6$, 298 K) and excerpt of the PH region.

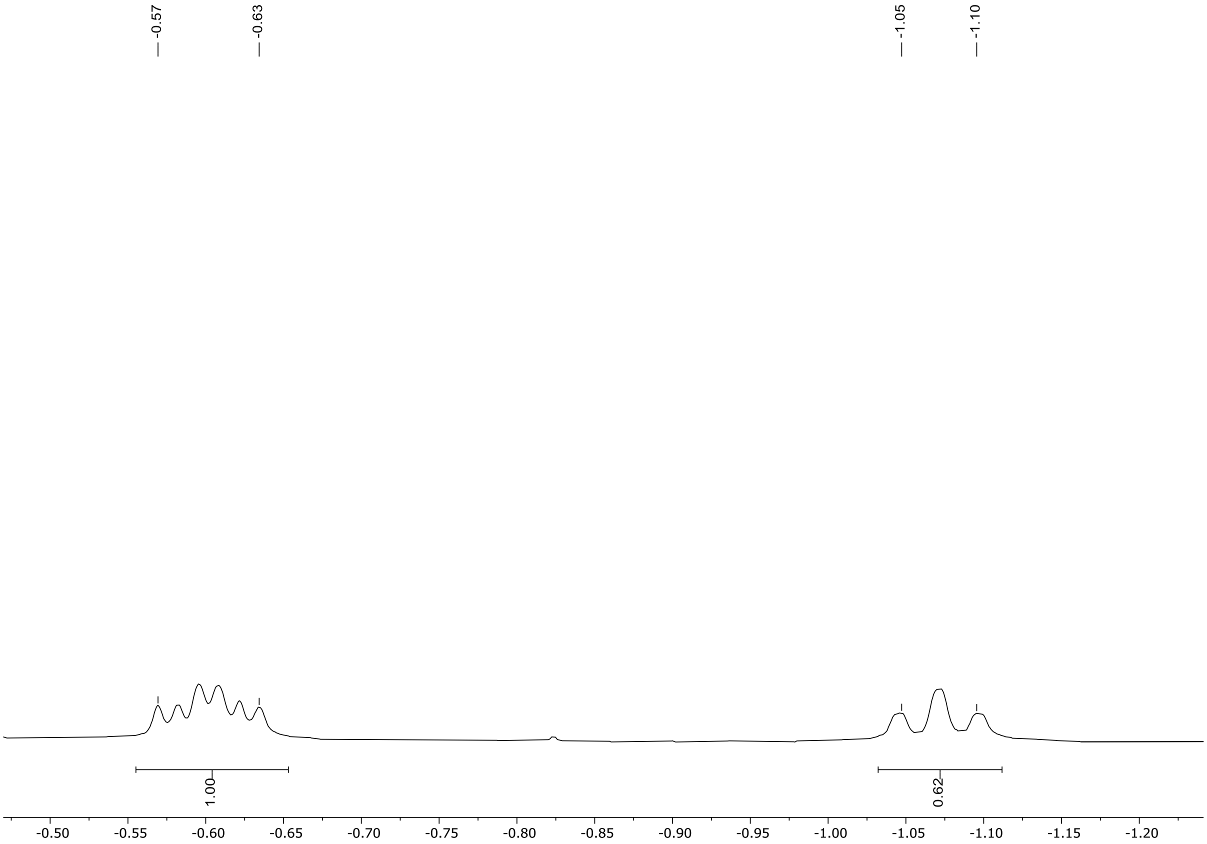

Figure S62. Excerpt of the ^{1}H NMR spectrum of the crystalline material after the reaction of $^{Dipp}TerSn(CH_2P(CH_3)_2)=P^{Dipp}Ter$ (**Sn2d**) / $^{Dipp}TerSn(CH_2P(CH_3)_2)=P^{Dipp}Ter$ (**Sn3**) with HCCPh showing the highfield shifted CH_2 signals (400 MHz, C_6D_6, 298 K).

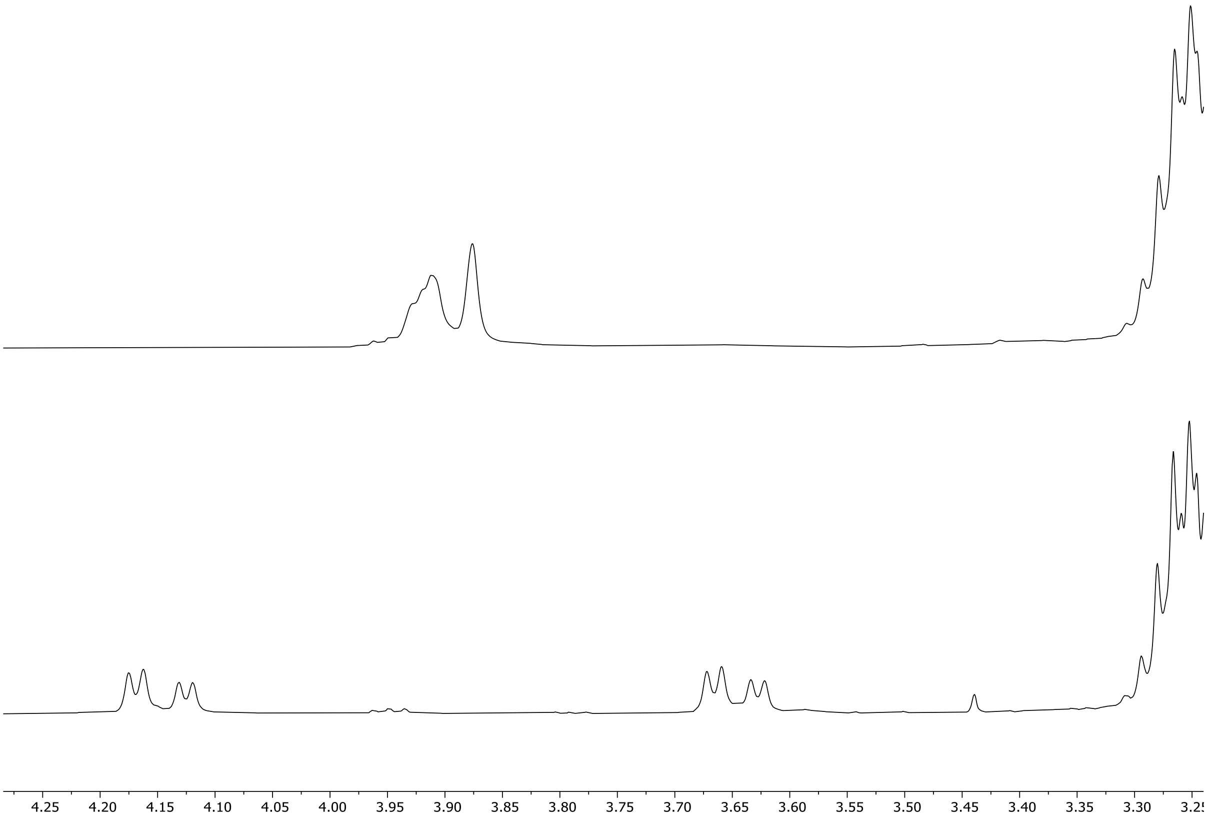

Figure S63. Excerpt of the ^{1}H NMR spectrum (bottom) and excerpt of the $^{1}H\{^{31}P\}$ NMR spectrum (top) of the PH region (400 MHz, C_6D_6, 298 K).

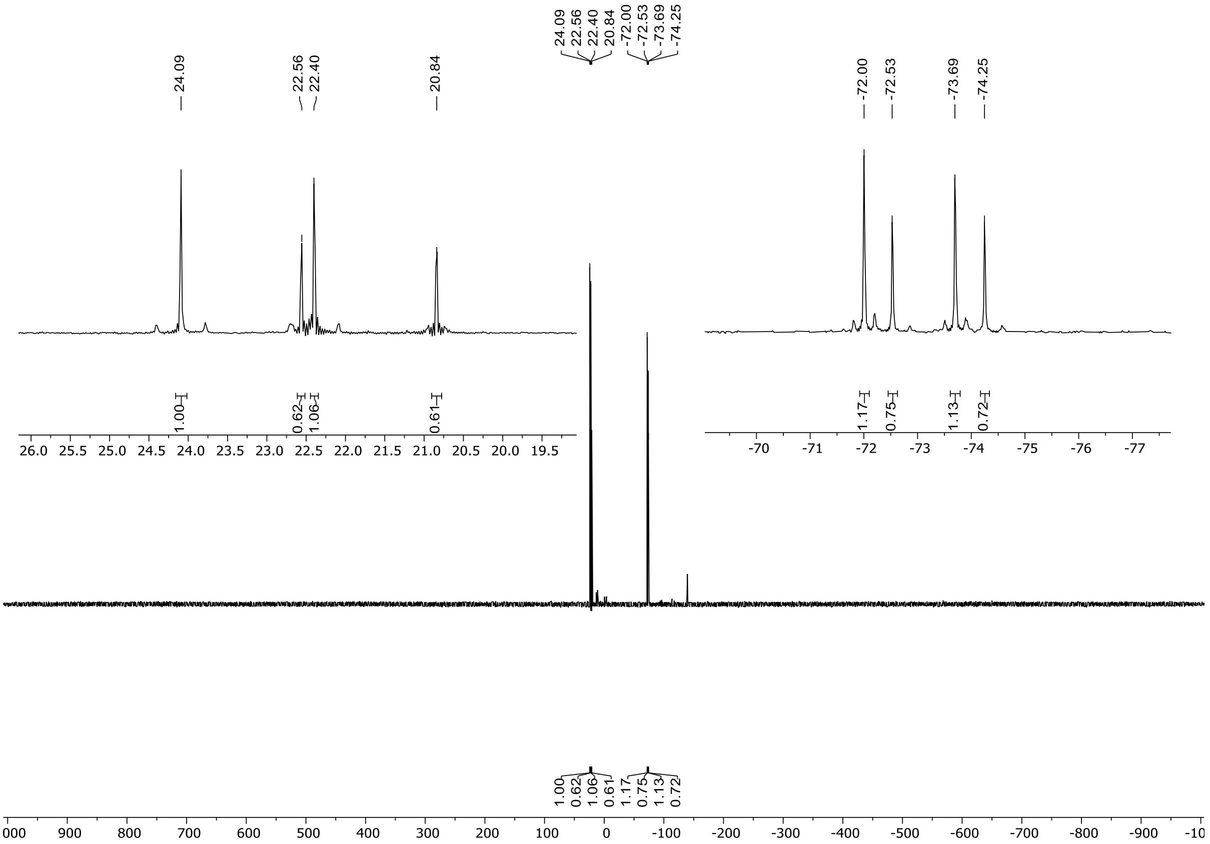

Figure S64. $^{31}P\{^{1}H\}$ NMR spectrum of the crystalline material after the reaction of $^{Dipp}TerSn(CH_2P(CH_3)_2)=P^{Dipp}Ter$ (**Sn2d**) / $^{Dipp}TerSn(CH_2P(CH_3)_2)=P^{Dipp}Ter$ (**Sn3**) with HCCPh (161 MHz, C_6D_6, 298 K).

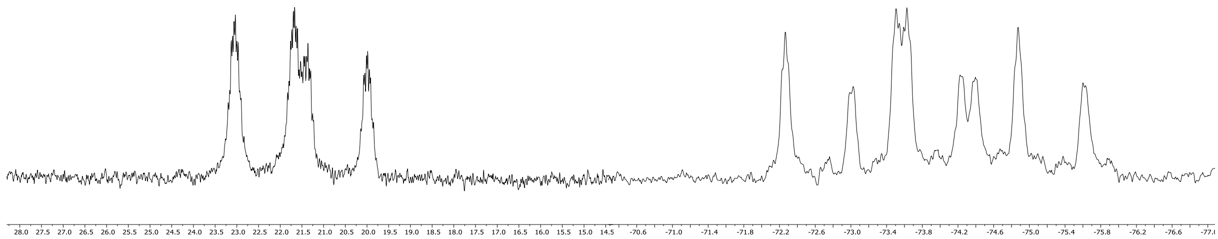

Figure S65. Excerpts of the ^{31}P NMR spectrum of the crystalline material after the reaction of $^{Dipp}TerSn(CH_2P(CH_3)_2)=P^{Dipp}Ter$ (**Sn2d**) / $^{Dipp}TerSn(CH_2P(CH_3)_2)=P^{Dipp}Ter$ (**Sn3**) with HCCPh (161 MHz, C_6D_6, 298 K).

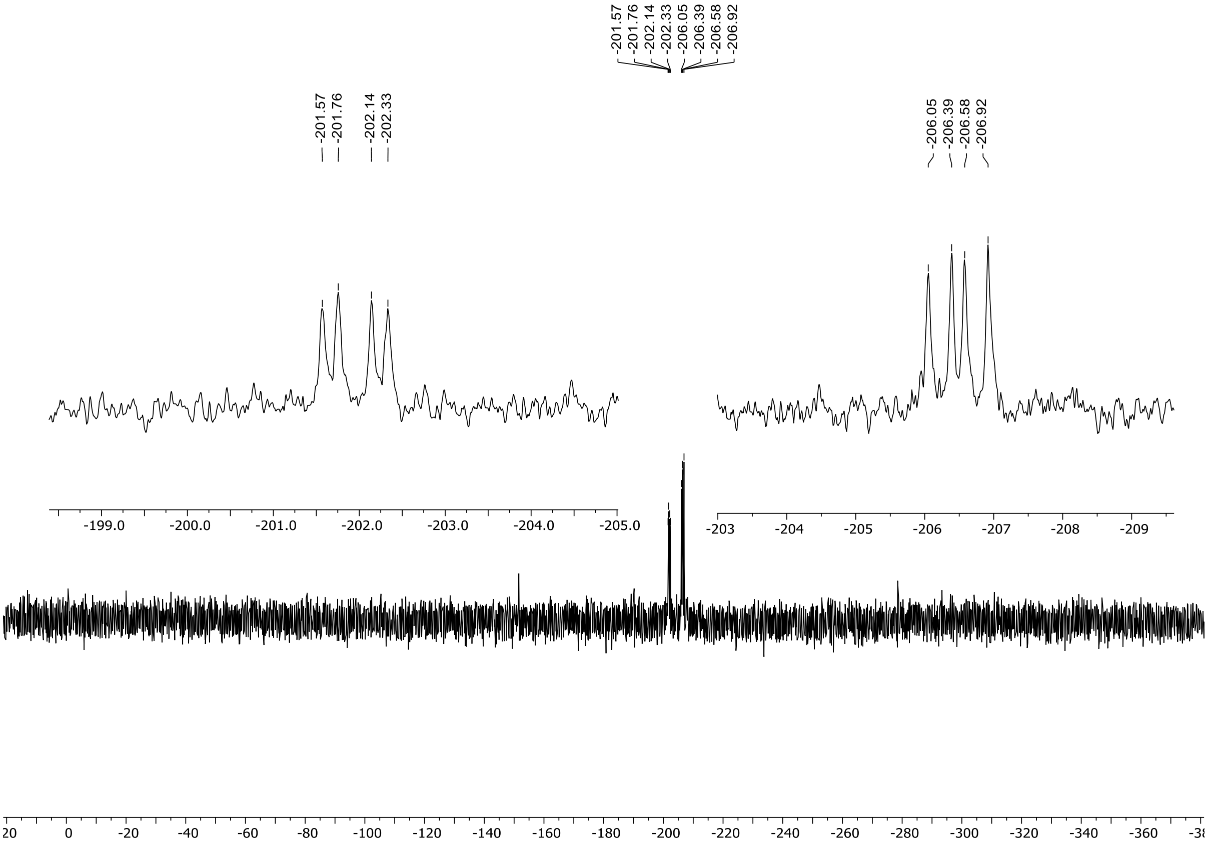

Figure S66. ^{119}Sn{^{1}H} NMR spectrum of the crystalline material after the reaction of ^{Dipp}TerSn($CH_2P(CH_3)_2$)=P^{Dipp}Ter (**Sn2d**) / ^{Dipp}TerSn($CH_2P(CH_3)_2$)=P^{Dipp}Ter (**Sn3**) with HCCPh (149 MHz, C_6D_6, 298 K).

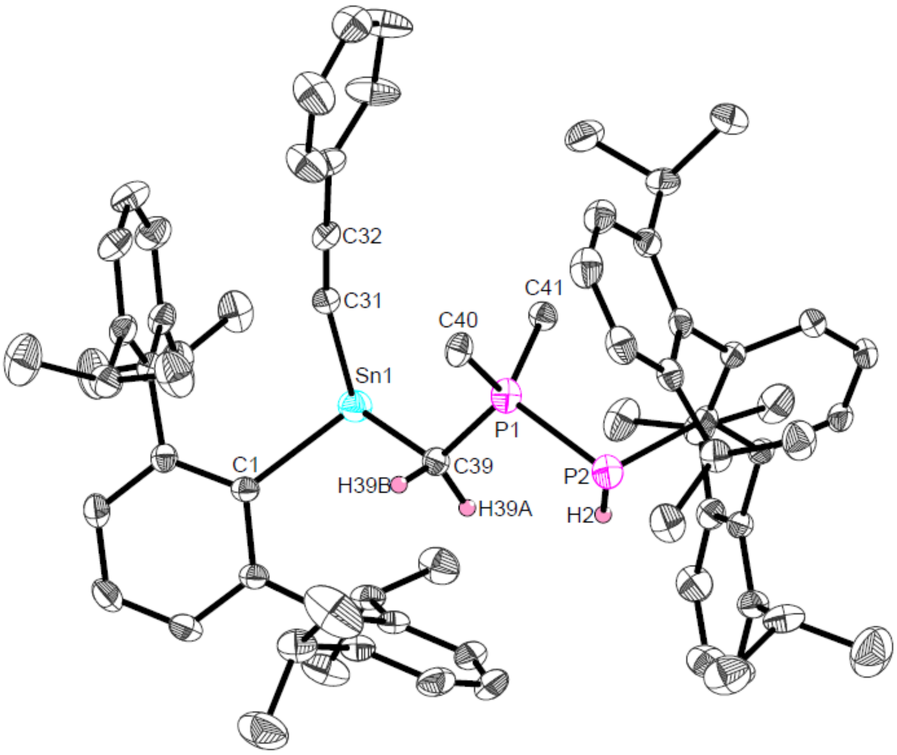

Figure S67. Left: Molecular structure of ^{Dipp}TerSn(CCPh)$CH_2P(CH_3)_2$P(H)^{Dipp}Ter (**Sn6**) in the crystal. Thermal ellipsoids are drawn at the 50% probability level (hydrogen atoms except H2, H39A and H39B have been omitted for clarity). Selected bond lengths (Å) and angles (deg): Sn1–C1 2.250(4), Sn1–C31 2.203(4), Sn1–C39 2.301(4), P1–C39 1.756(4), P1–P2 2.1997(13), C1–Sn1–C31 103.39(14), C1–Sn1–C39 97.52(14), C31–Sn1–C39 89.81(15).

Reaction of ^{Dipp}TerSn(CH$_2$P(CH$_3$)$_2$)=PMes* (Sn2b) with HCCPh

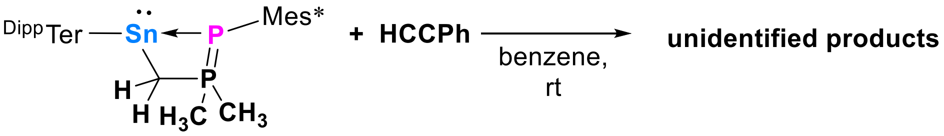

^{Dipp}TerSn(CH$_2$P(CH$_3$)$_2$)=PMes* (**Sn2b**) (0.020 g, 0.035 mmol) was dissolved in 0.6 mL of C_6D_6 and transferred into a Young NMR tube followed by addition of 2 drops of phenylacetylene. The reaction progress was monitored by ^{1}H and ^{31}P{^{1}H} NMR spectroscopy revealing no reaction after 16 h at room temperature and the formation of several unidentified products after another 16 h at 60 °C.

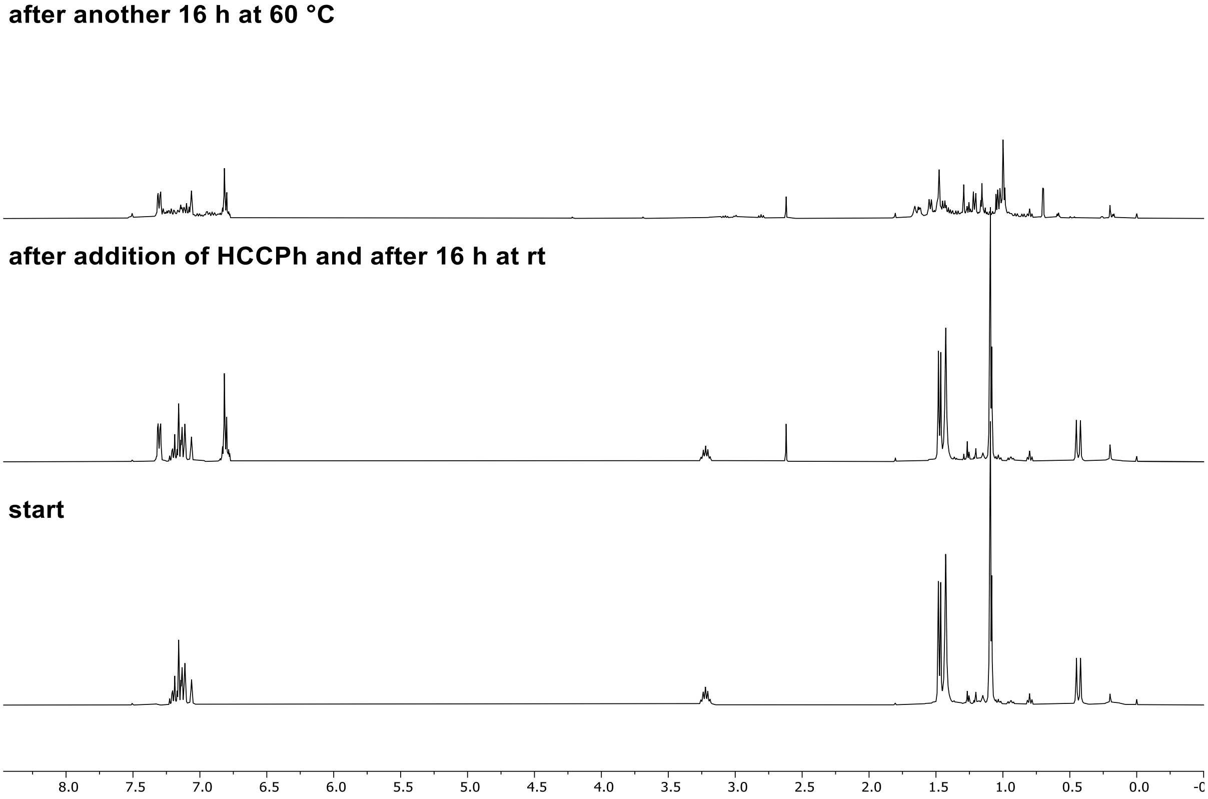

Figure S68. Monitoring of the reaction of ^{Dipp}TerSnCH$_2$P(CH$_3$)$_2$=PMes* (**Sn2b**) with HCCPh via ^{1}H NMR spectroscopy (400 MHz, C_6D_6, 298 K).

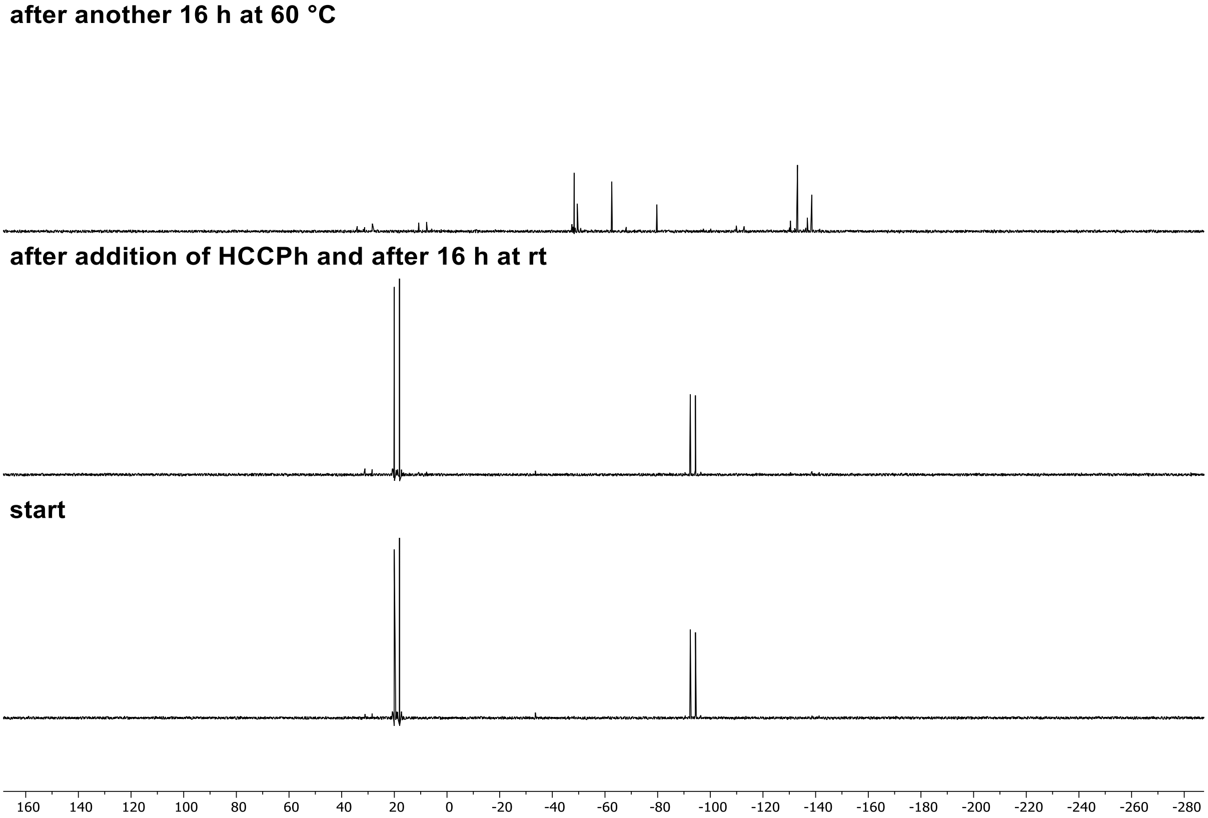

Figure S69. Monitoring of the reaction of ^{Dipp}TerSnCH$_2$P(CH$_3$)$_2$=PMes* (**Sn2b**) with HCCPh via $^{31}P\{^1H\}$ NMR spectroscopy (161 MHz, C_6D_6, 298 K).

Crystallographic Details

Single crystal X-ray diffraction data for al compounds were collected at 150 K on an Oxford Diffraction/Agilent SuperNova diffractometer using Cu-K$_\alpha$ radiation (λ = 1.54184 Å) or Mo-K$_\alpha$ radiation (λ = 0.71073 Å), and equipped with a nitrogen gas Oxford Cryosystems cooling unit.[S5] Raw frame data were reduced using CrysAlisPro.[S6] The structures were solved using SHELXT[S7] and refined to convergence on F^2 by full-matrix least-squares using SHELXL[S8] in combination with OLEX2.[S9] Distances and angles were calculated using the full covariance matrix. Restraints were used to maintain sensible geometries for the disordered groups and approximate the displacement parameters to typical values. Selected crystallographic data are summarized in tables S2-S4 and full details are given in the supplementary deposited CIF files (CCDC 2157153-2157161). These data can be obtained free of charge from the Cambridge Crystallographic Data Centre via https://www.ccdc.cam.ac.uk/data_request/cif.

Table S2. Crystal structure data for compounds **Sn1a**, **Sn1b** and **Sn2b**

	**Sn1a**	**Sn1b**	**Sn2b**
CCDC	2157157	2157161	2157160
empirical formula	$C_{30}H_{43}NSi_2Sn$	$C_{36}H_{55}NSi_2Sn$	$C_{51}H_{74}P_2Sn(0.5\ C_7H_{16})$
fw	592.52	676.68	917.83
colour	orange	orange	yellow
habit	block	block	plate
cryst dimens, mm	0.20 x 0.10 x 0.10	0.33 x 0.17 x 0.13	0.43 x 0.24 x 0.03
cryst syst	triclinic	orthorhombic	monoclinic
space group	P1	$P2_12_12_1$	$P2_1/n$
a, Å	12.2933(5)	11.89030(10)	10.4553(4)
b, Å	14.6704(6)	17.3025(2)	30.8618(14)
c, Å	18.4462(4)	18.1060(2)	17.2386(6)
α, deg	97.376(2)	90	90
β, deg	91.256(3)	90	106.817(4)
γ, deg	113.744(4)	90	90
V, Å^3	3010.0(2)	3724.98(7)	5324.5(4)
Z	4	4	4
D_{calc}, g•cm^{-3}	1.308	1.207	1.145
μ, mm^{-1}	7.633	6.227	0.572
T, K	150.01(10)	150.01(10)	150.01(10)
θ range, deg	3.917 – 76.315	3.5980 – 76.2450	2.7670 – 29.2680
no. of rflns collected	12465	7769	14613
no. of indep rflns	10833	7605	9918
R(int)	0.0590	0.0344	0.0573
max, min transmission	1.00000 and 0.39344	1.00000 and 0.53027	1.000 and 0.618
[I>2σ(I)]	R1 = 0.0585<br>wR2 = 0.1422	R1 = 0.0196<br>wR2 = 0.0463	R1 = 0.0515<br>wR2 = 0.1153
R indices (all data)	R1 = 0.0648<br>wR2 = 0.1511	R1 = 0.0205<br>wR2 = 0.0468	R1 = 0.0928<br>wR2 = 0.1336
GOF on F^2	1.052	1.041	1.033

Table S3. Crystal structure data for compounds **Sn2c**, **Sn3** and **Sn4**

	**Sn2c**	**Sn3**	**Sn4**
CCDC	2157153	2157159	2157156
empirical formula	$C_{57}H_{70}P_2Sn(C_5H_{12})$	$C_{63}H_{82}P_2Sn(2\ C_6H_6)$	$C_{66}H_{88}P_4Sn_2\ (2\ C_6H_{14})$
fw	1007.90	1176.13	1414.96
colour	yellow	light red	clear orange
habit	block	block	needle
cryst dimens, mm	0.15 x 0.10 x 0.10	0.21 x 0.15 x 0.11	0.32 x 0.12 x 0.08
cryst syst	triclinic	monoclinic	triclinic
space group	P1	C2/c	P1
a, Å	11.7528(3)	21.77860(9)	11.8585(5)
b, Å	13.5480(3)	14.92177(6)	12.1490(5)
c, Å	18.0165(5)	40.57072(16)	15.1202(7)
α, deg	95.407(2)	90	67.273(4)
β, deg	90.443(2)	98.2268(4)	84.337(4)
γ, deg	98.089(2)	90	72.140(4)
V, Å^3	2826.89(12)	13048.81(9)	1911.90(16)
Z	2	8	1
D_{calc}, g•cm^{-3}	1.184	1.197	1.229
μ, mm^{-1}	4.386	3.872	6.266
T, K	150.01(10)	150.01(10)	150.00(10)
θ range, deg	4.2920 – 76.2570	3.603 – 76.204	3.917 – 76.355
no. of rflns collected	11687	13584	11475
no. of indep rflns	10232	12886	9578
R(int)	0.0450	0.0305	0.0364
max, min transmission	1.00000 and 0.84644	1.000 and 0.534	0.695 and 0.333
[I>2σ(I)]	R1 = 0.0428	R1 = 0.0290	R1 = 0.0670
	wR2 = 0.1058	wR2 = 0.0756	wR2 = 0.1765
R indices (all data)	R1 = 0.0509	R1 = 0.0307	R1 = 0.0756
	wR2 = 0.1115	wR2 = 0.0766	wR2 = 0.1825
GOF on F^2	1.034	1.061	1.025

Table S4. Crystal structure data for compounds **Sn5a**, **Sn5c** and **Sn6**

	**Sn5a**	**Sn5c**	**Sn6**
CCDC	2157155	2157158	2157154
empirical formula	$C_{58}H_{70}N_2P_2Sn(0.5\ C_6H_{14})$	$C_{64}H_{82}N_2P_2Sn(2\ C_5H_{12})$	$C_{71}H_{88}P_2Sn$
fw	1018.87	1204.23	1294.38
colour	colourless	yellow	light yellow
habit	block	block	plate
cryst dimens, mm	0.20 x 0.15 x 0.11	0.10 x 0.10 x 0.05	0.23 x 0.15 x 0.05
cryst syst	triclinic	triclinic	monoclinic
space group	P1	P1	$P2_1/c$
a, Å	11.7804(4)	16.3835(4)	13.67135(13)
b, Å	13.9681(3)	18.7699(4)	16.7658(2)
c, Å	18.7515(4)	23.3674(5)	34.7241(3)
α, deg	75.943(2)	77.990(2)	90
β, deg	79.799(2)	83.726(2)	97.4377(9)
γ, deg	69.893(3)	89.933(2)	90
V, $Å^3$	2795.85(14)	6984.8(3)	7892.19(14)
Z	2	4	4
D_{calc}, g•cm^{-3}	1.210	1.145	1.089
μ, mm^{-1}	4.452	3.632	3.239
T, K	150.01(17)	150.01(10)	150.01(10)
θ range, deg	3.437 – 76.218	3.512 – 76.382	3.680 – 76.356
no. of rflns collected	11586	28848	16454
no. of indep rflns	9649	21649	13560
R(int)	0.0417	0.0582	0.0807
max, min transmission	1.000 and 0.548	1.00000 and 0.91783	1.000 and 0.513
$[I>2\sigma(I)]$	R1 = 0.0432<br>wR2 = 0.1104	R1 = 0.0460<br>wR2 = 0.1087	R1 = 0.0568<br>wR2 = 0.1321
R indices (all data)	R1 = 0.0539<br>wR2 = 0.1186	R1 = 0.0703<br>wR2 = 0.1226	R1 = 0.0736<br>wR2 = 0.1431
GOF on F^2	1.028	1.009	1.176

Computational Details

All computations were carried out using the Gaussian 16 software package or the ORCA 5.0 software package.[S10,S11] Gas phase optimizations were carried out using the M06-2X functional[S12] and the def2-SVP basis set.[S13] Single point energy calculations and frequency analyses were then conducted on the optimized structures at the more accurate M06-2X/def2-TZVP[S12,S13] level of theory with the reaction solvent (benzene) modelled using a polarizable continuum model.[S14] Each system was treated with a Grimme (D3)[S15] dispersion correction. The optimized structures were confirmed to minima on the potential energy surface by the absence of imaginary frequencies. Natural bonding orbital (NBO) analyses were carried out using the NBO 7.0 program.[S16]

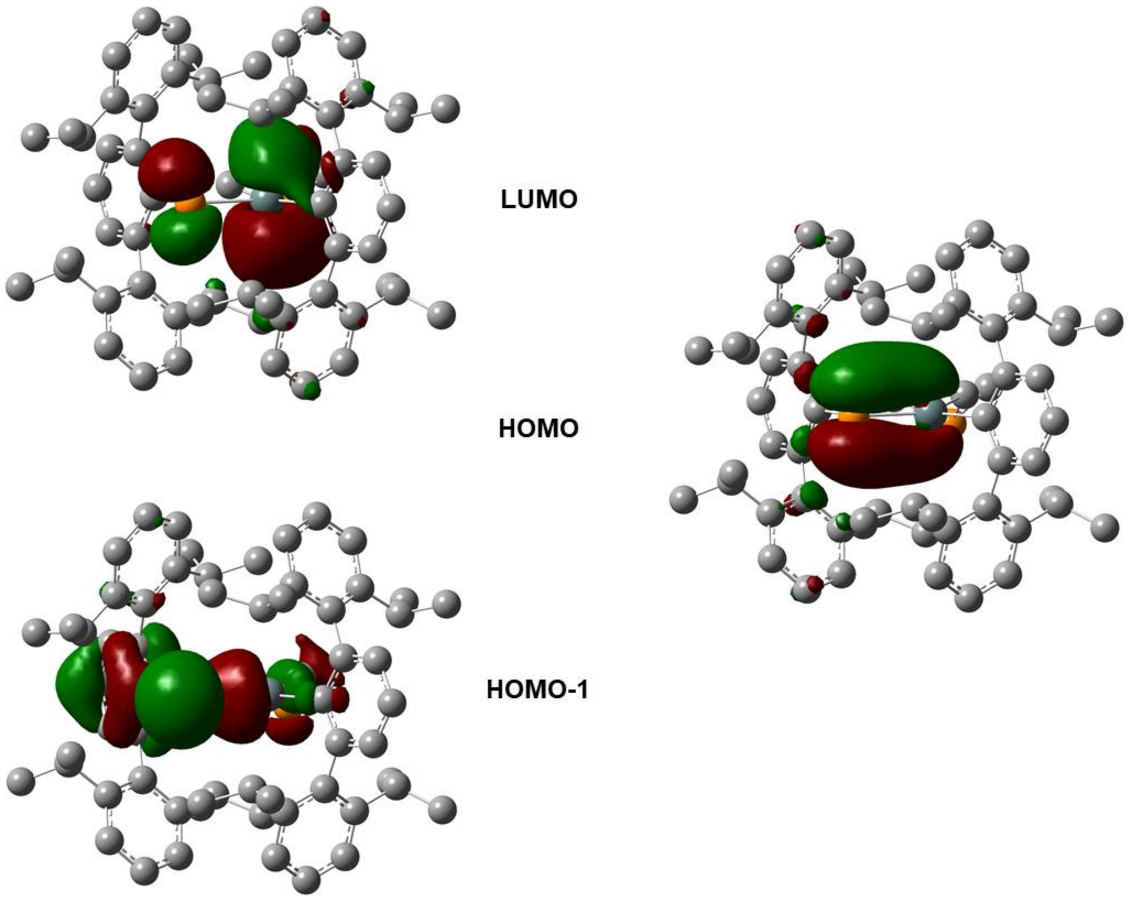

Figure S70. Selected molecular orbitals of the optimized structure of **Sn3** revealing the major bonding within the Sn=P moiety.

Table S5. Selected NBOs illustrating the major bonding interactions between tin and phosphorus in **Sn3**. Hydrogen atoms were omitted for clarity.

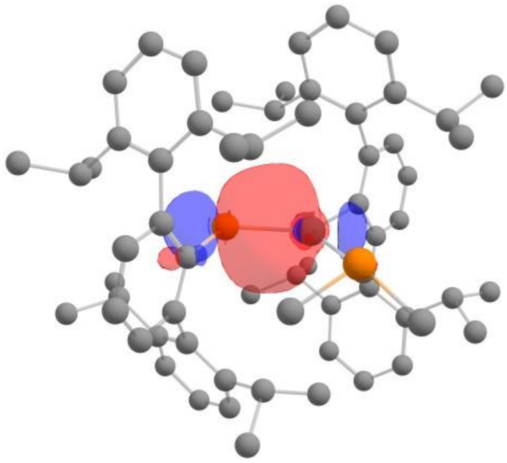

NBO 1 [SnP (σ)]

Occupancy: 1.79735e

31% Sn [**s** (37%), **p** (63%)]

69% P [**s** (13%), **p** (86%)]

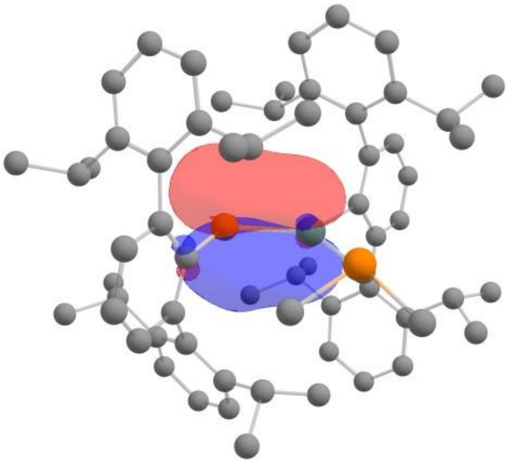

NBO 2 [SnP (π)]

Occupancy: 1.91275e

22% Sn [**p** (100%)]

78% P [**p** (99 %)]

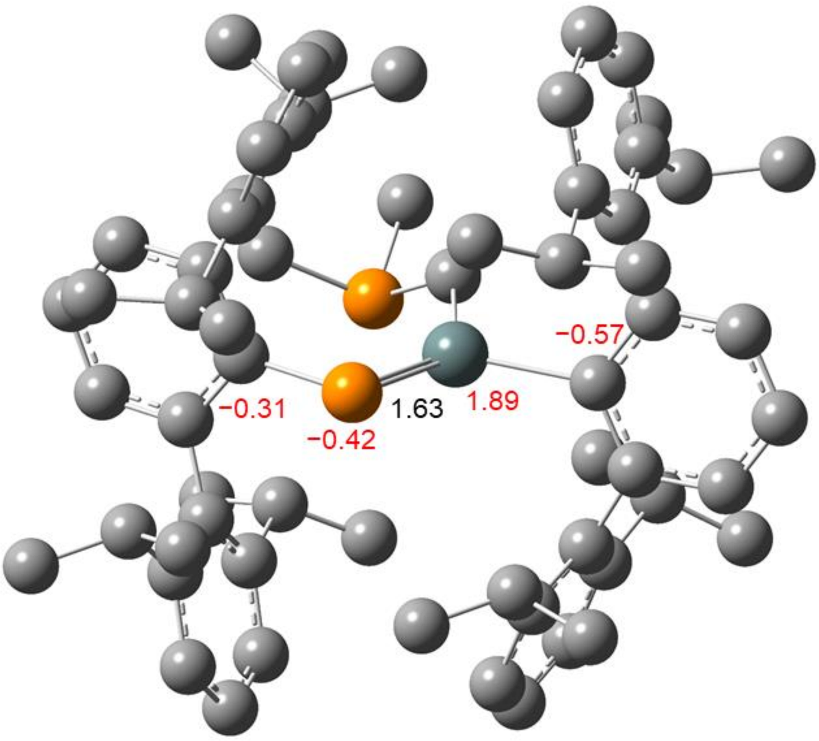

Figure S71. Optimized structure of **Sn3** with the tin-phosphorus Wiberg bond index (shown in black) and selected natural atomic charges (shown in red).

References

[S1] P. Gupta, J.-E. Siewert, T. Wellnitz, M. Fischer, W. Baumann, T. Beweries, C. Hering-Junghans, *Dalton Trans.* **2021**, *50*, 1838-1844.

[S2] M. Fischer, S. Nees, T. Kupfer, J. T. Goettel, H. Braunschweig, C. Hering-Junghans, *J. Am. Chem. Soc.* **2021**, *143*, 4106-4111.

[S3] P. J. Davidson, D. H. Harries, M. F. Lappert, *J. Chem. Soc., Dalton Trans.* **1976**, 2268-2274.

[S4] W. A. Merrill, E. Rivard, J. S. DeRopp, X. Wang, B. D. Ellis, J. C. Fettinger, B. Wrackmeyer, P. P. Power, *Inorg. Chem.* **2010**, *49*, 8481-8486.

[S5] J. Cosier, A. M. Glazer, *J. Appl. Cryst.* **1986**, *19*, 105-107.

[S6] CrystAlisPro, Oxford Diffraction/Agilent Technologies UK Ltd, Yarnton, UK.

[S7] G. Sheldrick, *Acta Cryst. C* **2015**, *71*, 3-8.

[S8] G. Sheldrick, *Acta Cryst. A* **2008**, *64*, 112-122.

[S9] O. V. Dolomanov, L. J. Bourhis, R. J. Gildea, J. A. K. Howard, H. Puschmann, *J. Appl. Cryst.* **2009**, *42*, 339-341.

[S10] Gaussian 16, Revision C.01, M. J. Frisch, G. W. Trucks, H. B. Schlegel, G. E. Scuseria, M. A. Robb, J. R. Cheeseman, G. Scalmani, V. Barone, G. A. Petersson, H. Nakatsuji, X. Li, M. Caricato, A. V. Marenich, J. Bloino, B. G. Janesko, R. Gomperts, B. Mennucci, H. P. Hratchian, J. V. Ortiz, A. F. IzmaylovJ. J. L. Sonnenberg, D. Williams-Young, F. Ding, F. Lipparini, F. Egidi, J. Goings, B. Peng, A. Petrone, T. Henderson, D. Ranasinghe, V. G. Zakrzewski, J. Gao, N. Rega, G. Zheng, W. Liang, M. Hada, M. Ehara, K. Toyota, R. Fukuda, J. Hasegawa, M. Ishida, T. Nakajima, Y. Honda, O. Kitao, H. Nakai, T. Vreven, K. Throssell, J. A. Montgomery, Jr., J. E. Peralta, F. Ogliaro, M. J. Bearpark, J. J. Heyd, E. N. Brothers, K. N. Kudin, V. N. Staroverov, T. A. Keith, R. Kobayashi, J. Normand, K. Raghavachari, A. P. Rendell, J. C. Burant, S. S. Iyengar, J. Tomasi, M. Cossi, J. M. Millam, M. Klene, C. Adamo, R. Cammi, J. W. Ochterski, R. L. Martin, K. Morokuma, O. Farkas, J. B. Foresman, and D. J. Fox, Gaussian, Inc., Wallingford CT, 2016.

[S11] F. Neese, F. Wennmohs, U. Becker, C. Riplinger, *J. Chem. Phys.* **2020**, *152*, 224108.

[S12] Y. Zhao and D. G. Truhlar, *Theor. Chem. Acc.* **2008**, *120*, 215-241.

[S13] F. Weigend, R. Ahlrichs, *Phys. Chem. Chem. Phys.* **2005**, *7*, 3297-3305.

[S14] J. Tomasi, B. Mennucci, R. Cammi, *Chem. Rev.* **2005**, *105*, 2999-3094.

[S15] S. Grimme, J. Antony, S. Ehrlich, H. Krieg, *J. Chem. Phys.*, **2010**, *132*, 154104.

[S16] NBO 7.0, E. D. Glendening, J, K. Badenhoop, A. E. Reed, J. E. Carpenter, J. A. Bohmann, C. M. Morales, P. Karafilogou, C. R. Landis, F. Weinhold, Theoretical Chemistry Institute, University of Wisconsin, Madison (2018).
